# Identification of *Sympetrum depressiusculum* Sélys, 1841 in South Korea (Odonata: Libellulidae) According to Morphology and Genetic Markers

**DOI:** 10.3390/insects14090733

**Published:** 2023-08-30

**Authors:** Jee-Young Pyo, Sung-Soo Kim, Jeong Sun Park, Jong-Moon Kim, Yang-Keun Song, Iksoo Kim

**Affiliations:** 1 Department of Applied Biology, College of Agriculture & Life Sciences, Chonnam National University, Gwangju 61186, Republic of Korea; knh5368@naver.com (J.-Y.P.); jungsun5009@naver.com (J.S.P.); 2 Research Institute for East Asian Environment and Biology, Seoul 05207, Republic of Korea

**Keywords:** Odonata, dragonfly, *Sympetrum depressiusculum*, *Sympetrum frequens*, *COI*, *16S rRNA*, ITS, Korean Peninsula, clinal variation

## Abstract

**Simple Summary:**

Two species of *Sympetrum*, namely *S. depressiusculum* and *S. frequens*, have been documented in South Korea. However, the distinction between these two species and their identity within South Korean populations has been a longstanding point of contention. To solve this issue, morphology, two mitochondrial genes, and one nuclear region were analyzed for *S. depressiusculum* samples from The Netherlands and Russia and *S. frequens* samples from Japan, as well as samples of *Sympetrum* from South Korea. Further, available public sequence data for the two species were included. Morphology, sequence divergence, and phylogenetic results all consistently suggest that South Korean populations form a single species. Analyses of haplotype network and gene pool distribution patterns in a nuclear region conducted to better explain the current taxonomic implications indicated changes in the dominant gene pool from The Netherlands and Russia to South Korea and Japan. However, such divergence and subdivision could be explained within the context of within-species diversification patterns, suggesting that South Korean populations constitute one species, *S. depressiusculum*, by applying the senior name.

**Abstract:**

In South Korea, both *Sympetrum depressiusculum* Sélys, 1841 (Odonata: Libellulidae), which is distributed throughout Europe and from Russia to the Korean Peninsula, and *Sympetrum frequens* Sélys, 1883, which is endemic to Japan, are recorded. However, the identity of South Korean populations and the validity of listing the two species have not yet been settled. In this study, we collected seventy-four individuals of *Sympetrum* species from South Korea (five localities), Russia, The Netherlands, and Japan. These samples were examined for morphology and sequenced for partial *COI*, *16S rRNA*, and a nuclear internal spacer (ITS) region, after which these molecular data were combined with available public data from Russia, Japan, and The Netherlands. Major morphological characters that have been used to distinguish the two species and phylogenetic, network, and structure analyses all consistently suggest that South Korean populations form a single species. Consequently, it could be valid to treat South Korean populations as one species, *S. depressiusculum*, by applying the senior name. Nevertheless, the validity of maintaining each as an independent species in other countries may need additional study considering that our samples were focused more on South Korea and limited for Europe, Russia, and Japan.

## 1. Introduction

The genus *Sympetrum* Newman, 1833, consists of ~60 species of darters that are distributed in temperate zones of the Northern Hemisphere, except for Australia [1]. With a relatively small body length (≤40 mm), most mature male *Sympetrum* are bright red in part or all of their bodies [1]. Members of this genus occur in small ponds, wetlands, and rice paddies, where the water current is stopped or slow [2]. Previous phylogenetic analysis of *Sympetrum* using mitochondrial and nuclear sequences along with morphological characters showed that dispersal, enabled by their strong flight capability, and historical vicariance events are responsible for the current distribution of *Sympetrum* [3].

Among *Sympetrum* species, *Sympetrum frequens* Sélys, 1883, which was originally described using type locality samples from Japan, is distributed in Japan, whereas *Sympetrum depressiusculum* Sélys, 1841, which was originally described using type locality samples from Eurasia, is distributed throughout Europe, Far East Russia, and the Korean Peninsula [4,5]. In Europe, *S. depressiusculum* is ranked as vulnerable on the European Red List [6,7]. In contrast, the species is more numerous in Central Asia [8] and is one of the most abundant species of Odonata in South Korea (Korean name: Daeryukgochujomjamjari). In Japan, *S. frequens* is also a particularly abundant species [9,10,11]. *S. depressiusculum* prefers small, temporary aquatic habitats, particularly those found in alluvial regions of rivers and lakes within water-logged meadows in Europe. However, this species is also known to inhabit anthropogenic habitats, including winter-dry rice fields [12,13]. On the other hand, *S. frequens* thrives in various lowland areas across Japan, typically close to rice fields and cooler mountainous regions, with its habitat range extending to the sea coast [14].

There have been several conflicting opinions on the taxonomic status and distribution of these two species. *S. frequens*, endemic to Japan, has been considered to be an independent insular vicariant species of *S. depressiusculum*, distinguishable from *S. depressiusculum* based on a subtle but recognizable difference in genitalia and shape of pterothoracic black stripe [9]. This understanding is further supported by the observation that *S. depressiusculum* individuals arriving to Japan, which dispersed infrequently from the Korean Peninsula and Asian mainland, are distinguishable from the *S. frequens* dwelling in Japan [9,15]. On the other hand, some have proposed an alternative claim that *S. frequens* individuals should be included in the species *S. depressiusculum*, particularly because the morphology of the male vesica spermalis of *S. frequens* is very similar to that of *S. depressiusculum* [4,5,16,17]. Karyotyping results supported this notion in that chromosome numbers and karyotype morphology of both species are essentially similar [18].

Populations of the Korean Peninsula and Far East Russia have been recognized to exhibit intermediate morphological features between *Sympetrum* species of Europe and Japan, but these former populations have been proposed to belong to *S. depressiusculum* rather than *S. frequens* [4,5]. Moreover, Sawabe et al. [19] collected *S. depressiusculum* in several localities throughout South Korea and, among the majority of obvious *S. depressiusculum* samples, the authors found several *S. depressiusculum* individuals with a thick black stripe on the thorax, making them morphologically similar to *S. frequens*. However, mitochondrial *16S rRNA* sequence analysis of the *S. frequens*-like *S. depressiusculum*, obvious *S. depressiusculum*, and Japanese *S. frequens* showed no sequence divergence among these samples; rather, these samples formed a single large group, suggesting that *S. frequens* and *S. depressiusculum* should be assigned to a single species with the senior name *S. depressiusculum*. On the other hand, some have proposed that *S. depressiusculum* in South Korea should belong to *S. frequens* instead of *S. depressiusculum* [20,21]. Furthermore, current records of insects occurring in South Korea list both *S. depressiusculum* and *S. frequens* [22], reflecting the previous finding that adult *S. frequens* and *S. depressiusculum* can be distinguished by the shape of female genitalia, although no discernable difference was found in male genitalia [20]. Thus, it is necessary to clarify the identity and validity of these two species in South Korea. However, it is essential to compare the South Korean populations to neighboring populations, particularly European and Russian populations, which have never been compared with those in South Korea, to better interpret the status of South Korean populations.

In this study, a total of 74 adult *Sympetrum* specimens were collected from diverse locations including South Korea, Russia, The Netherlands, and Japan. Next, we examined the primary morphological characteristics that have frequently been employed to differentiate between the two species. Additionally, we sequenced the mitochondrial *COI*, *16S rRNA*, and a nuclear internal spacer (ITS) region. These sequences were then combined with existing public data to facilitate the analysis of sequence divergence, phylogenetic connections, and the population’s genetic structure. These analyses were conducted to clarify the identity of South Korean populations and to validate the recognition of two distinct *Sympetrum* species within the region.

## 2. Materials and Methods

### 2.1. Samples

**Figure 1:**
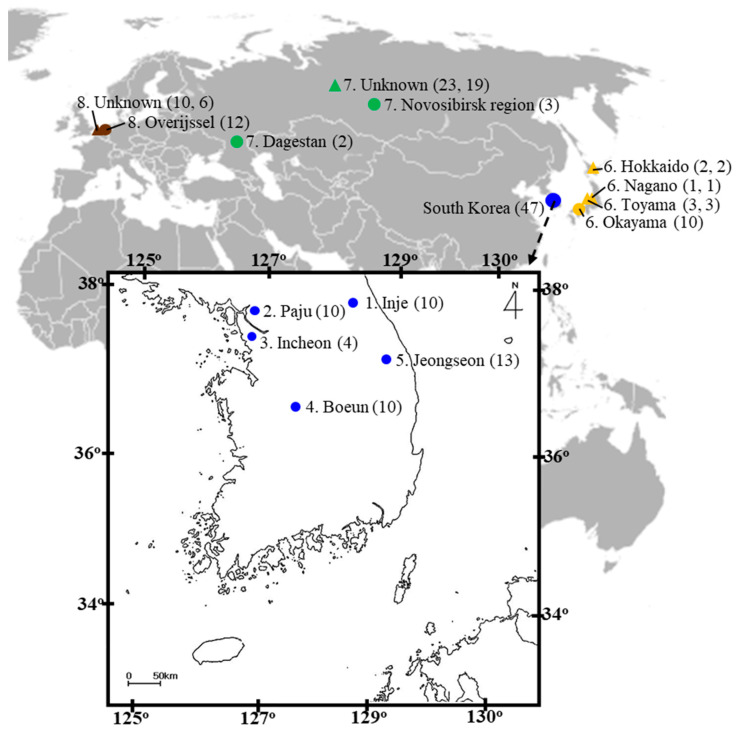
Sampling localities. 1–5 (blue), South Korea; 6 (yellow), Japan; 7 (green), Russia; 8 (brown), The Netherlands. Circles represent samples collected in the current study, while triangles represent samples from public data. The numbers in parentheses indicate the number of individuals sequenced in this study or collected from public data. In the case of public data, the first and second values are the number of sequences for *COI* + *16S rRNA* and the ITS region, respectively. See Table S1 for site details.

A total of 74 adult *Sympetrum* belonging either to *S. depressiusculum* or *S. frequens* were collected from five localities in South Korea, two localities in Russia, and one locality each in Japan and The Netherlands (Figure 1). Site details are presented in Table S1. Due to the small sample size in each Russian locality (five samples in total) two locality samples were treated as a single population in subsequent analyses. Once collected, individuals were examined for morphological characteristics, such as body color, adult genitalia of both sexes, body length, basifrontal black stripe, and pterothoracic black stripe, that are usually helpful in distinguishing the two species, according to previous studies [9,23].

### 2.2. DNA Extraction, Amplification, and Sequencing

Total DNA was extracted from one or two hind legs using a Wizard Genomic DNA Purification Kit (Promega, Madison, WI, USA), following the manufacturer’s instructions. To amplify segments of each 451 bp fragment of mitochondrial *COI*, 276 bp fragment of *16S rRNA*, and 805 bp fragment of the nuclear ITS region, consisting of *18S rRNA*, ITS1, *5.8S rRNA*, ITS2, and *28S rRNA*, primers were selected from previous studies (Table S2) [24,25,26,27,28,29]. PCR amplification was performed using the following conditions: an initial denaturation step at 94 °C for 7 min, 35 amplification cycles (denaturation at 94 °C for 1 min, annealing at 50–57 °C for 1 min, and extension at 72 °C for 1 min), and a final extension step at 72 °C for 7 min using AccuPower^®^ PCR PreMix (Bioneer, Daejeon, Republic of Korea). Electrophoresis was performed in 0.5× Tris-acetate EDTA buffer on 1% agarose gels to confirm successful DNA amplification. PCR products were then purified using a PCR purification kit (Bioneer). All PCR products were directly sequenced in both directions (Macrogen Co., Seoul, Republic of Korea) on an ABI 3730xl automated DNA sequencer (PE Applied Biosystems, Foster City, CA, USA). However, several individuals provided dubious sequences in at least one position in the ITS region. These amplicons were cloned using a T-Blunt^TM^ PCR cloning kit (SolGent, Daejeon, Republic of Korea) and HITTM DH5α High 108 competent cells (Real Biotech Co., Banqiao City, Taiwan). The resultant plasmid DNA was isolated using a Plasmid Mini Extraction Kit (Bioneer, Daejeon, Republic of Korea).

### 2.3. Sequence Analysis

*COI*, *16S rRNA*, and the ITS region were directly sequenced for the 74 individuals, but 16 individuals (9 individuals from South Korea, 1 from Russia, 5 from The Netherlands, and 1 from Japan) showed a dimorphic (di-allelic) pattern of amplification in at least 1 site in the ITS region. For these samples, five clones per individual were sequenced (Table S3). The sequences were aligned using MAFFT version 7 [30]. The nucleotide sequences of the *COI* gene were translated using the genetic code for invertebrate mitochondrial DNA to detect the potential presence of pseudogene sequences. However, none of the sequences exhibited any indication of such pseudogene occurrences. Furthermore, to verify the accuracy of each sequence, a BLAST search (http://blast.ncbi.nlm.nih.gov/Blast.cgi, accessed on 15 October 2022) was performed.

### 2.4. Public Data

*COI*, *16S rRNA*, and ITS region sequences for *S. depressiusculum* and *S. frequens* from previous studies were downloaded from GenBank [31,32,33,34,35] (Futahashi et al., unpublished data). The compiled sequences consisted of 6 sequences each from *COI*, *16S rRNA*, and ITS sourced from Japan; 23 sequences from both *COI* and *16S rRNA*, as well as 19 ITS sequences from Russia; and 10 sequences both from *COI* and *16S rRNA*, in addition to 6 ITS sequences from The Netherlands (Table S3). The sequences from Japan had specified origins, but this information was unavailable for the sequences from Russia and The Netherlands. GenBank-registered ITS region sequences were available for 746 bp, so our 805 bp-long sequences were trimmed to 746 bp when GenBank data were used with our own data. Sequences from Sawabe et al. [19] were not available, so they were not included in the current analysis. We also excluded a few sequences that were substantially shorter than our own, so as not to lose sequence information for subsequent analyses.

### 2.5. Haplotype Designation

Individual sequences differing by one or more nucleotides in *COI*, *16S rRNA*, and the concatenated sequences of *COI* and *16S rRNA* or with insertions/deletions (indels) in the ITS region were designated as different haplotypes by performing unordered pairwise comparisons among sequences using PAUP version 4.0b [36]. Haplotype designations were applied to new sequences as they were discovered (i.e., SCOI01, SCOI02, SCOI03, and so forth for *COI*; and S16S01, S16S02, S16S03, and so forth for *16S rRNA*; and SMT01, SMT02, SMT03, and so forth for the concatenated sequences of the two mitochondrial genes). For our own 805 bp-long ITS haplotype, names were assigned as SITS01, SITS02, SITS03, and so forth, but for our trimmed 746 bp-long sequences, new haplotype names were created along with public data. However, original haplotype numbers were maintained by keeping the original number after the dash for reference in the sample list in Table S3 (e.g., SITS12 for 805 bp and SITS01-12 for 746 bp).

### 2.6. Phylogenetic and Network Analyses

To understand the degree of haplotype divergence, we calculated the unrooted pairwise distances between haplotypes for each mitochondrial gene, the concatenated sequences of both mitochondrial genes, and the ITS region. These calculations were conducted using PAUP version 4.0b [36]. The alignment of each gene (or region for ITS) included *Sympetrum infuscatum* as an outgroup for subsequent phylogenetic analyses (GenBank accession numbers LC366870 for *COI*, LC366573 for *16S rRNA*, and LC366276 for ITS region) [33]. This alignment was conducted using MAFFT version 7 [30] and was further adjusted to align with GenBank data lengths using Gblock 0.91b [37], which resulted in a reduction of the ITS region alignment from 805 to 746 bp. The *COI* and *16S rRNA* sequences were combined into a unified alignment using SequenceMatrix version 1.9 [38] to match file formats suitable for phylogenetic and network analyses. Clustal X version 1.83 [39] was employed to alter the file format for phylogenetic analysis, whereas network analysis was conducted using DnaSP version 6.12.03 (Universitat de Barcelona, Barcelona, Spain) [40].

Phylogenetic analyses were performed for the concatenated sequences of the two mitochondrial genes and ITS using the Bayesian inference (BI) and maximum-likelihood (ML) methods, respectively. The HKY+G for *COI*, HKY+I for *16S rRNA*, and K2P for the ITS region were selected as the best substitution models using Modeltest version 3.7 [41] within the IQ-TREE web server [42]. BI analysis was performed using MrBayes version 3.2.7 [43], which is incorporated into the CIPRES Portal version 3.1 [44]. Here, we conducted two separate runs of an incrementally heated Markov Chain Monte Carlo (MCMC), consisting of four chains (one cold chain and three hot chains). These runs were carried out simultaneously for ten million generations to analyze the concatenated sequences of the two mitochondrial genes, and thirty million generations for the ITS region, with sampling performed every 1000 generations. Trace plots and convergence diagnostics were examined using MrBayes and Tracer version 1.7 [45] to ensure that the Markov chains achieved stationarity and converged on parameter estimates and tree topology. This assessment included the verification of the standard deviation of split frequencies (<0.01) and the effective sample size (>200) after the burn-in phase, which was set at 25%. The confidence values are expressed as Bayesian posterior probabilities (BPPs) in percentages for the BI tree. For ML analysis, we employed IQ-TREE [42]. Branch support was assessed using 1000 replicates of the Ultrafast Bootstrap (UFBoot) [46,47] and 1000 replicates of the Shimodaira–Hasegawa approximate likelihood ratio test (SH-aLRT) [48]. The generated trees were viewed using FigTree version 1.4.4 (http://tree.bio.ed.ac.uk/software/figtree). To further scrutinize haplotype relationships, we utilized the median-joining algorithm integrated into PopArt version 1.7 [49].

### 2.7. Gene Pool Analysis

The genetic structure of *S. depressiusculum* and *S. frequens* populations (country-wide for Japan, Russia, and The Netherlands, but for five individual localities for South Korea) was analyzed using Bayesian Analysis of Population Structure (BAPS) version 6.0 [50]. Analysis was performed using clustering, with a linked locus option and an independent model. In this process, population clusters were estimated for *K* values ranging from 1 to 20. 

## 3. Results

### 3.1. Morphological Characteristics of South Korean Populations

**Figure 2:**
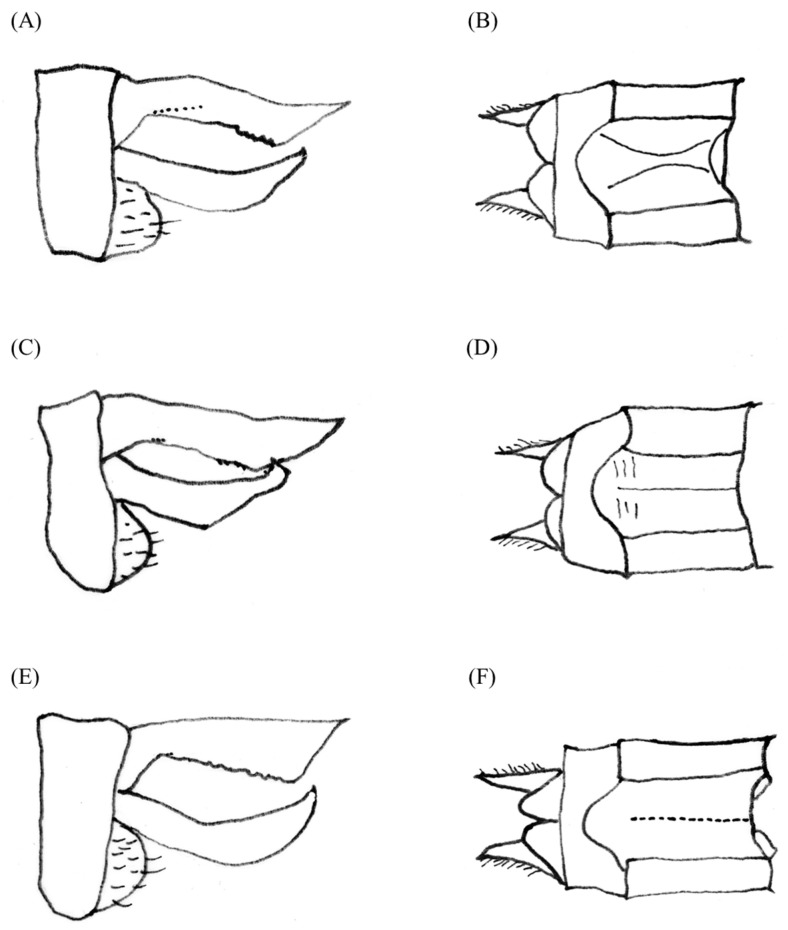
Genitalia of *Sympetrum* species: (**A**) 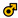, South Korea; (**B**) 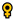, South Korea; (**C**) 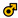, The Netherlands and Russia; (**D**) 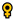, The Netherlands and Russia; (**E**) 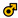, Japan; (**F**) 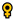, Japan.

**Figure 3:**
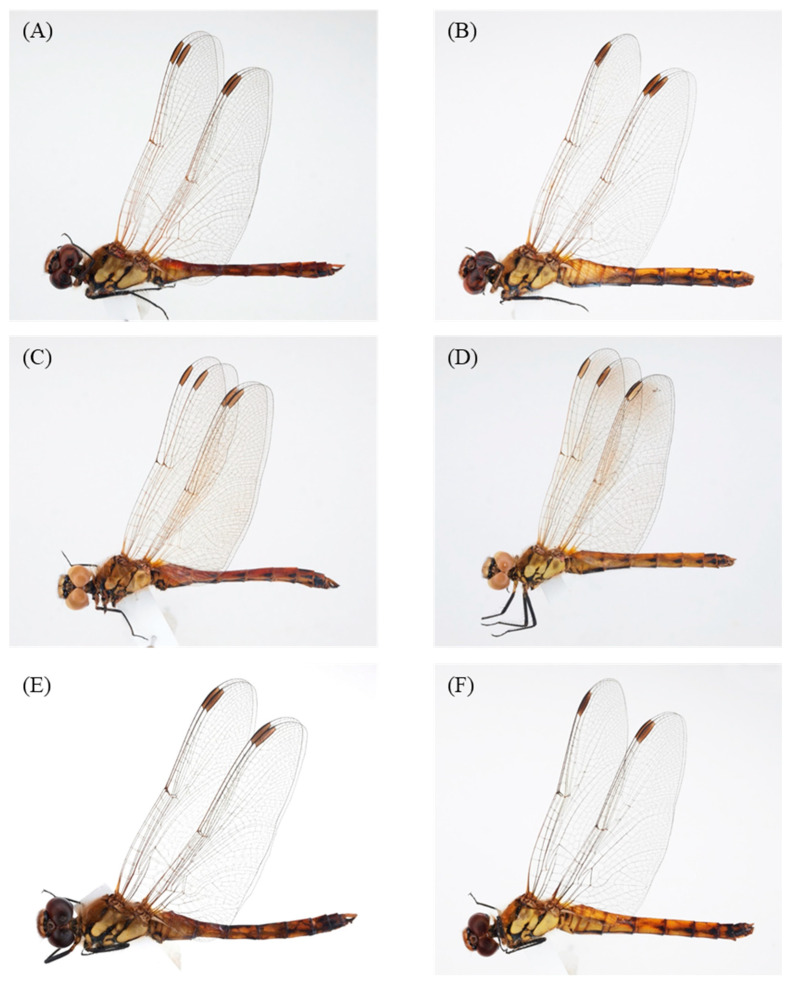
Collected *Sympetrum* species: (**A**) 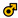, South Korea (Inje); (**B**) 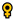, South Korea (Inje); (**C**) 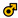, The Netherlands; (**D**) 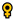, The Netherlands; (**E**) 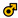, Japan; (**F**) 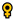, Japan.

**Figure 4:**
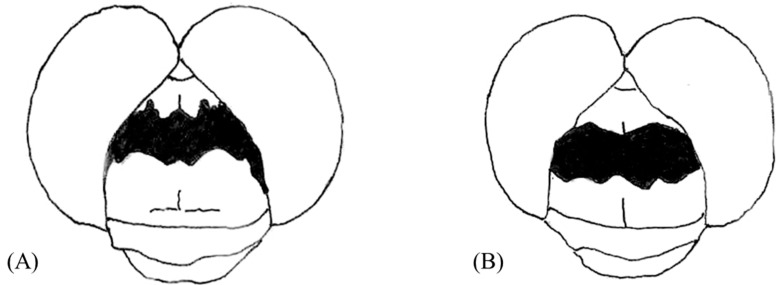
Head of *Sympetrum* species: (**A**) The Netherlands; (**B**) Japan.

**Figure 5:**
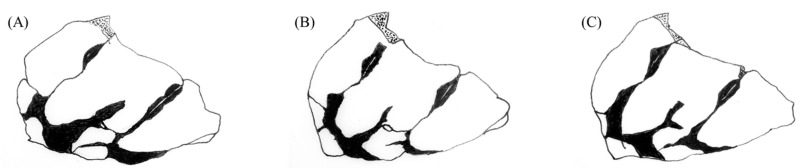
Pterothoracic black stripe of *Sympetrum* species: (**A**) South Korea; (**B**) The Netherlands and Russia; (**C**) Japan.

**Figure 6:**
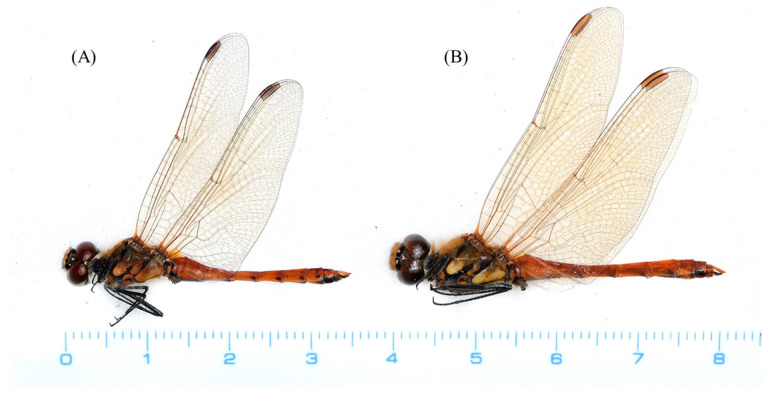
*Sympetrum* species with different body sizes collected in South Korea: (**A**) *Sympetrum* species with a smaller body size collected in Incheon; (**B**) *Sympetrum* species with a typical body size collected in Inje.

The male genitalia displayed minimal variation across the samples from different countries (Figure 2). The sole distinction observed in *S. frequens* collected from Japan compared to samples from other countries was a slightly thicker and more sharply truncated superior appendage when viewed laterally. Similarly, the female genitalia exhibited no significant differences, except for a slight protrusion at the end of the 10th abdominal segment in *S. frequens* compared to samples from other countries. In addition to subtle differences in genitalia in both sexes among country samples, we found that *Sympetrum* individuals collected in South Korea generally had a longer hindwing than those collected in The Netherlands, providing 29–34 mm, resembling *S. frequens* in Japan (Figure 3). However, *Sympetrum* individuals collected in South Korea had a more strongly undulating basifrontal black stripe than *S. frequens* (Figure 4) in addition to slightly narrower lateral thoracic dark stripes, which resembled those of *S. depressiusculum* in Europe (Figure 5). The lateral thoracic stripes of *Sympetrum* individuals collected in South Korea were slightly thicker than those of individuals collected in The Netherlands and Russia, but the difference was insignificant. In contrast, the lateral thoracic stripes of *S. frequens* were slightly thicker than those of *Sympetrum* individuals from Europe and South Korea, albeit with some variation between samples (Figure 5). Further, both European and South Korean samples had two first two lateral stripes that are connected to each other, but *S. frequens* has a weak connection to each other (Figure 5). Thus, South Korean samples bear a greater resemblance to European populations in terms of the basifrontal black stripe and thoracic black stripes. Unlike the majority of South Korean samples, four individuals collected in the west-central part of South Korea (locality 3, Incheon; Figure 1) exhibited the typical morphology of *S. depressiusculum* in Europe, including a smaller body size (Figure 6).

### 3.2. Dimorphic Sites in the ITS Region

Sixteen individuals collected in this study revealed dimorphism at one or two positions in ITS region sequences (Table S3, Figure S1). Sequencing of five clones per individual provided the corresponding two nucleotides at the dimorphic sites in some individuals, whereas only a single nucleotide was found where dimorphic sites were previously detected in other individuals (Figure S1). Further, for a few individuals, additional substitution was identified at sites in which no dubious sequences were detected in the direct sequencing. Collectively, 57 haplotypes from 138 sequences (74 individuals plus 4 additional clones from 16 individuals) were obtained from our own sequencing. Futahashi et al. [24] has shown that dimorphic sties of ITS1 sequence are possible indicators that are useful to decide hybridization between two *Sympetrum* species, such as *S. croceolum* and *S. speciosum*. However, our ITS analysis showed that dimorphic sites are found in all country samples including obvious *S. depressiusculum* distributed in The Netherlands and Incheon, South Korea. Thus, an application of ITS dimorphism as evidence of hybridization between *S. depressiusculum* and *S. frequens* remains unsolved.

### 3.3. Haplotype Distribution

The 74 individuals analyzed in this study provided 46 *COI*, 15 *16S rRNA*, 50 *COI* + *16S rRNA*, and 57 ITS haplotypes (Table S3). When 39 *COI* sequences from public data were considered together with the current data, 65 haplotypes were generated (Table S4). Among these haplotypes, nine were shared between countries, whereas the remaining 56 haplotypes were country-specific. In the case of The Netherlands, the addition of 10 individuals from public data to our own data still revealed one identical haplotype (SCOI32), and this haplotype was not shared with samples from any other countries. Excluding SCOI32, specific to The Netherlands, SCOI11 provided the highest frequency of 8.8% (10 individuals) with a relatively wide distribution, and SCOI05 provided the widest distribution with a relatively high frequency of 4.4% (five individuals). Thus, the haplotype distribution of *COI* can be characterized as a limited local distribution in most haplotypes, with some exceptions.

From the *16S rRNA*, 19 haplotypes were generated when 39 sequences from public data were considered together with our own data (Table S5). Among these haplotypes, four were common between countries, whereas the remaining 16 haplotypes were country-specific. From The Netherlands, a single haplotype was also detected (S16S01), but this haplotype was shared with all considered countries. Thus, the haplotype distribution of *16S rRNA* can also be characterized as a limited local distribution in most haplotypes, except for S16S01. A total of 73 haplotypes were generated from the concatenated sequences of the mitochondrial genes (Table S6). Among these, only four haplotypes were found to be shared between South Korea and Russia, whereas the remaining 69 were country-specific.

From the ITS region, 56 haplotypes were generated when 31 sequences from public data were considered together with our own data (Table S7). Among these haplotypes, three were shared between countries: SITS01 among all South Korean localities and all countries excluding The Netherlands, with the highest frequency at 40.2% (68 individuals and clones); SITS10 among all countries, with the second highest frequency of 17.8% (30 individuals and clones); and SITS26 between Russia and The Netherlands, with a frequency of 2.4% (four individuals and clones). The remaining 53 haplotypes were country-specific. SITS01 showed the highest frequency in South Korea and Japan. On the other hand, SITS10 possessed the highest frequency in The Netherlands and Russia. In this way, the two haplotypes with higher frequencies showed differences in their geographic dominance. Thus, the haplotype distribution of the ITS region can also be summarized as a limited local distribution in most haplotypes, with the primary exception of SITS01 and SITS10.

### 3.4. Haplotype Divergence

Sequence divergence among the 65 *COI* haplotypes ranged from 0.22% (1 bp) to 2.0% (9 bp), with the maximum sequence divergence (MSD) detected in the comparison of SCOI07 (Japan) to SCOI08 (Japan) and SCOI09 (Japan) and the comparison of SCOI16 (Paju, South Korea; locality 2) to SCOI08 (Japan) and SCOI09 (Japan) (Table S8). In addition, variation among haplotypes, without any divergent haplotype, was detected (Figure S2). Compared to that of *COI* sequences, the divergence and number of haplotypes of *16S rRNA* was markedly low (Table S9). Nineteen haplotypes also showed overall variation, without any divergent haplotype (Figure S2). When two mitochondrial genes are concatenated sequence divergence ranged from 0.14% (1 bp) to 1.38% (10 bp), with the MSD detected in the comparison of SMT28 (Boeun, South Korea) to SMT08 (Japan) and SMT09 (Japan) (Table S10). No divergent haplotype was detected when distribution of pairwise distance was examined (Figure S2). Sequence divergence among the 56 haplotypes of the ITS region showed overall variation among haplotypes, without any divergent haplotype (Figure S2). Haplotypes ranged from 0.13% (1 bp) to 1.61% (12 bp including insertion and deletion), with the MSD detected in the comparison of SITS20 (Boeun, South Korea) to SITS33 (The Netherlands) (Table S11).

### 3.5. Phylogenetic Analysis

**Figure 7:**
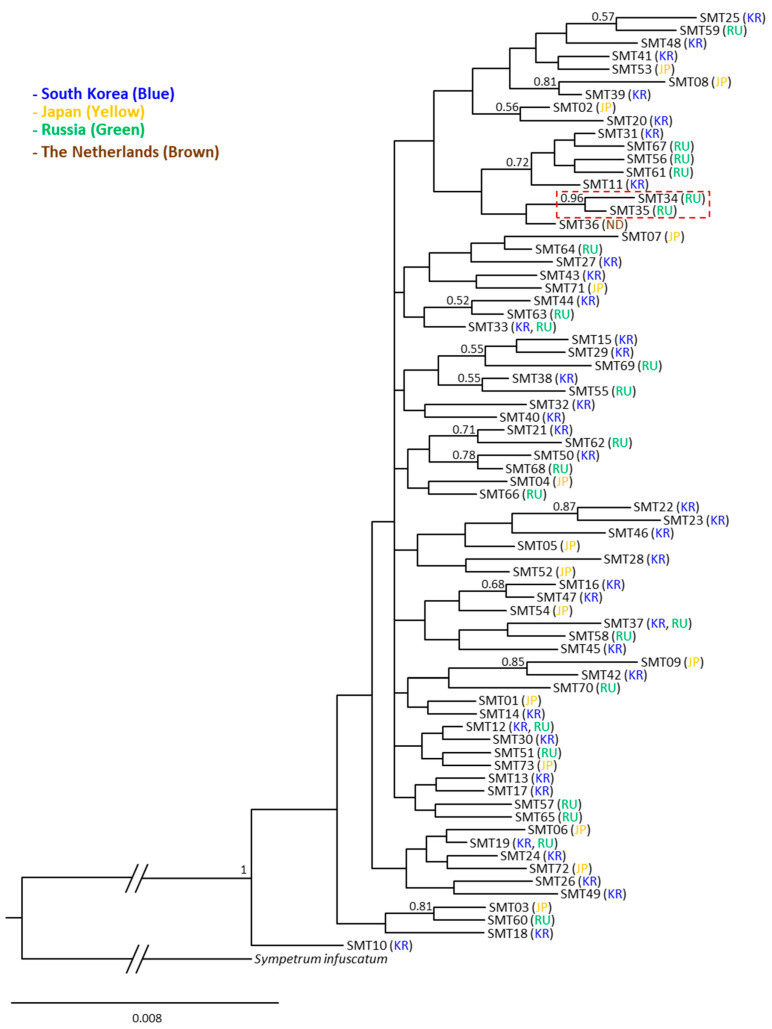
Phylogeny of Sympetrum *COI* + *16S rRNA* haplotypes obtained from the current study and public data using the Bayesian inference method. The numbers at each node specify Bayesian posterior probabilities (BPPs). BPPs below 0.5 are omitted. The scale bar indicates the number of substitutions per site. The abbreviations within parentheses indicate the country where the haplotypes were found (KR, South Korea; JP, Japan; RU, Russia; ND, The Netherlands). The branch length of *Sympetrum infuscatum* was truncated to approximately one-fifth of its true length due to the limited space. The dotted box indicates the subgroup supported with a BPP at 0.96.

**Figure 8:**
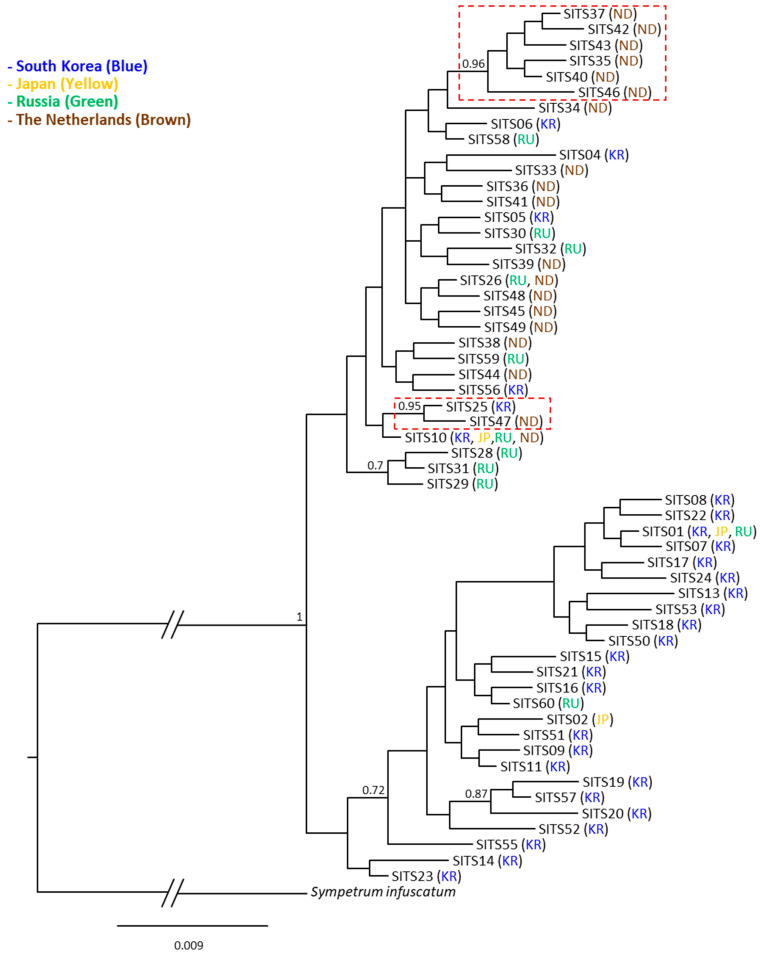
Phylogeny of *Sympetrum* ITS haplotypes obtained from the current study and public data using Bayesian inference method. The numbers at each node specify Bayesian posterior probabilities (BPPs). BPPs below 0.5 are omitted. The scale bar indicates the number of substitutions per site. The abbreviations within parentheses indicate the country where the haplotypes were found (KR, South Korea; JP, Japan; RU, Russia; ND, The Netherlands). The branch length of *Sympetrum infuscatum* was truncated to approximately one-fourth of its true length due to the limited space. The dotted box indicates the subgroup supported with a BPP ≥ 0.90.

The phylogenetic analysis using 73 haplotypes derived from the concatenated sequences of the two mitochondrial genes revealed generally weak nodal support for most subgroups in the BI analysis (Figure 7). The only discernible subgroup was comprised of SMT34 (Russia) and SMT35 (Russia), which garnered a substantial Bayesian posterior probability (BPP) of 0.96. This outcome strongly implies the absence of a detectable pattern based on country grouping. This is despite the fact that the majority of haplotypes are unique to specific countries (69 out of 73 haplotypes). ML-based analysis exhibited a few more numbers of subgroups with more robust support, indicated by higher SH-aLRT values (≥80%) and UFBoot values (≥95%). Nonetheless, the majority of these subgroups did not align with haplotypes exclusive to specific countries (Figure S3). Phylogenetic analysis with ITS haplotypes also revealed that the nodal support for most subgroups was generally weak. Particularly, we identified a single large group that was clearly differentiated from the outgroup, and country-specific groups were absent both in the BI (Figure 8) and ML analyses (Figure S4). The BI analysis revealed only two robustly supported subgroups (>0.90). One of these subgroups solely consisted of individuals from The Netherlands, whereas the other subgroup encompassed individuals from both South Korea and The Netherlands. In contrast, the ML-based analysis did not yield any single subgroup with higher SH-aLRT values (≥80%) and UFBoot values (≥95%), resulting in the formation of a single large group (Figure S4).

### 3.6. Network Analysis

**Figure 9:**
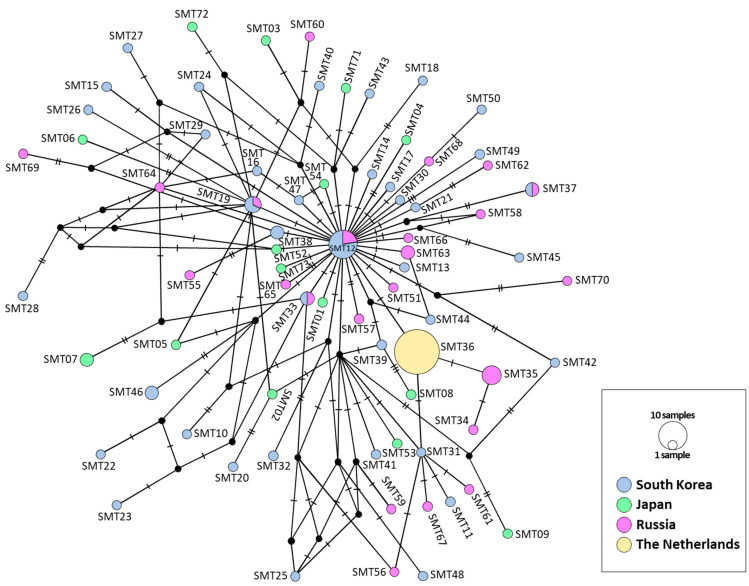
Median-joining network of *COI* + *16S rRNA* haplotypes of *Sympetrum* spp. obtained from the current study and public data. Each circle represents a haplotype and its colors represent a country. The circle size indicates the relative frequency of sequences belonging to a particular haplotype. Hatch marks along the network branches indicate the number of mutations.

The network analysis employing the 73 haplotypes derived from the concatenated sequences of the two mitochondrial genes yielded a single, expansive star-like phylogeny punctuated by several subgroups. These subgroups interconnect with varying distances to both each other and the central star-like phylogenetic group (Figure 9). However, no clearly distinguishable group comprising haplotypes exclusively found in a specific country was discernible (Figure 9). Notably, the haplotype SMT12, present in four localities within South Korea and Russia, occupied a central position within the main star-like phylogeny. This particular haplotype seemed to have played a role in the diversification of numerous haplotypes and subgroups.

**Figure 10:**
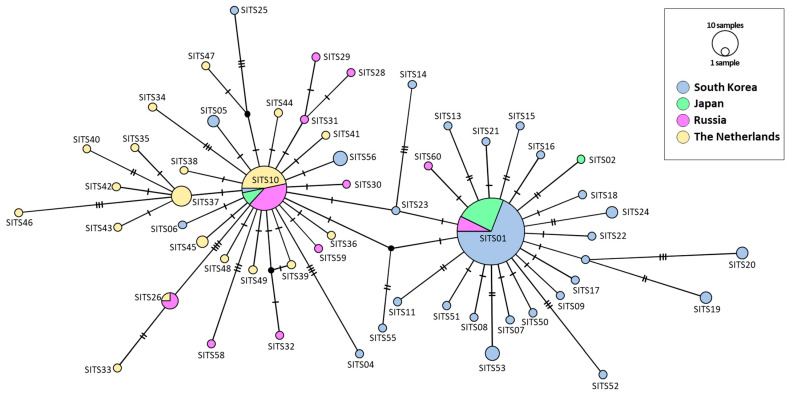
Median-joining network of ITS haplotypes of *Sympetrum* spp. obtained from the current study and public data. Each circle represents a haplotype, and its colors represent a country. The circle size indicates the relative frequency of sequences belonging to a particular haplotype. Hatch marks along the network branches indicate the number of mutations.

For the ITS region, two distinct star-like phylogenies emerged: one stemming from SITS10, which was identified in South Korea (one locality), Japan, Russia, and The Netherlands; and the other originating from SITS01, found in all localities in South Korea, Japan, and Russia (Figure 10). The former group encompassed members from all countries, with a greater representation of haplotypes from The Netherlands and Russia. The latter group contained more haplotypes from South Korea and Japan, without any representation from The Netherlands. Despite the discrete nature of these two subgroups in their respective star-like phylogenies, they were interconnected by three intermediate haplotypes solely found in South Korea (SITS14, SITS23, and SITS55). The genetic divergence between the two groups was a mere 0.27% (Table S11).

### 3.7. BAPS Analysis

**Figure 11:**
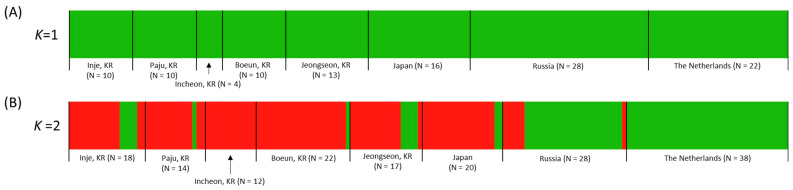
Bayesian Analysis of Population Structure (BAPS) of *Sympetrum* spp. using *COI* + *16S rRNA* (**A**) and ITS sequences (**B**) from our own data and public data. The optimum number of clusters (*K*) was 1 for *COI* + *16S rRNA* and 2 for the ITS region.

An evaluation of the likelihood scores from 10 repeated runs covering *K* values ranging from 1 to 20 in the BAPS analysis utilizing the concatenated sequences of the two mitochondrial genes revealed that all *Sympetrum* individuals fell within a singular optimal haplotype cluster, yielding no distinct groups (Figure 11A, *K* = 1, hereinafter referred to as haplogroup). In contrast, the analysis of the ITS region resulted in the identification of two haplogroups (red and green; Figure 11B). Among South Korean localities, with the exception of Incheon, two haplogroups (red and green) were evident, with the red haplogroup being predominant. Conversely, The Netherlands exclusively displayed the green haplogroup, whereas Russia exhibited two haplogroups, with the green haplogroup being dominant.

## 4. Discussion

The genus *Sympetrum* Newman 1833 (Libellulidae) encompasses more than 60 species, including *S. depressiusculum*, and is found in all continents except for Australia [1,51]. This extensive global distribution of the genus suggests that it is likely an ancient lineage, which originated approximately 50 million years ago during the Eocene epoch [3], a period marked by significant global vicariance events. The robust flight capabilities of this genus may have significantly contributed to its wide distribution and survival over its extended biogeographic history. Consistent with this notion, *S. depressiusculum* exhibited both morphological and molecular similarities across Europe and Asia.

### 4.1. Morphology

Although there is little difference in the morphology of genitalia between the two species, Asahina [9] described the morphological differences between *S. depressiusculum* and *S. frequens* as follows [3,52]:

1. The size of *S. depressiusculum* is smaller: *S. depressiusculum*, body length (from head to end of abdomen) ca. 30 mm, hindwing ca. 23 mm; *S. frequens*, body length ca. 40 mm, and hindwing ca. 30 mm.
2. Basifrontal dark stripe of *S. depressiusculum* is strongly undulated on front border with deep or distinct invagination on the sides, whereas that of *S. frequens* is broad, without deep invaginations on the sides.
3. Pterothoracic black stripe in *S. depressiusculum* is narrower, whereas that of *S. frequens* is broader.

The main takeaway from our morphological examination was that, though the two populations did differ in body size, South Korean samples most resembled European populations in terms of the basifrontal black stripe and thoracic black stripes (Figure 3, Figure 4 and Figure 5), which are considered to be important for distinguishing between *S. depressiusculum* and *S. frequens* [9,15].

**Figure 12:**
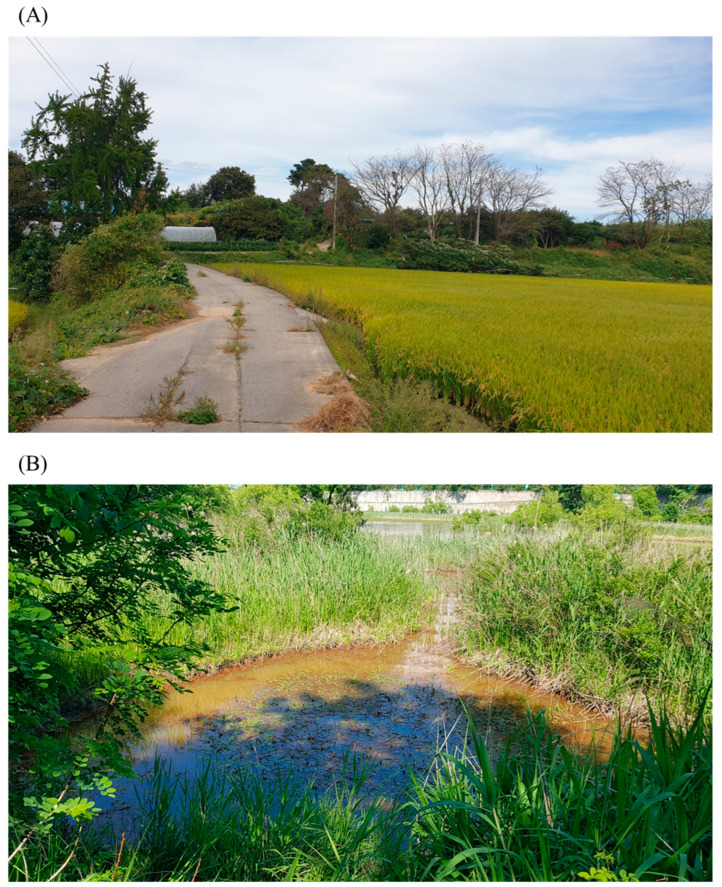
Photos of collection site in South Korea: (**A**) Rice field in Paju, Gyeonggi-do; (**B**) Swamp in Inje, Gangwon-do.

The body size difference between European populations and South Korea–Japan populations can probably be ascribed to habitat differences. *S. depressiusculum* exhibits distinct regional variations in habitat preferences, primarily driven by the availability of temporary unshaded, often shallow, and warm waters in Europe [53]. Despite its widespread presence, this species faces threats in Europe primarily due to habitat loss [6,7]. In response to the significant decline in its natural habitats, the species has adapted to various artificial habitats, including rice fields that dry out during winter [54]. In Southern Europe, *S. depressiusculum* is predominantly found in rice fields, whereas in Central Europe, it tends to inhabit lakes [53]. In Russia, *S. depressiusculum* inhabits estuaries of swampy areas and small rivulets that are surrounded by sedges and some reed patches along reservoir banks [55]. On the other hand, the Korean Peninsula and Japan are abundant in rice paddies and fields in autumn due to the occurrence of artificially stored stagnant water, although swamps are also widely present (Figure 12) [2,9,17]. Asahina [9,23] found that an *S. depressiusculum* population at a swamp, rather than the rice paddies, had a hindwing length ranging from 23 to 29 cm, morphologically resembling *S. depressiusculum* in Europe to Manchuria (in northeastern China). This habitat differs from the typical environments of South Korea, as it is situated in the northern highland of the Korean Peninsula, known as the Gaema Plateau (40°04′39.0″ N, 126°10′35.0″ E). Further, the current Incheon population of *S. depressiusculum*, which exhibited morphology and size typical of European populations, was also collected at a swamp, which is located very close to the Yellow Sea (approximately one kilometer to the seashore) in the west–central part of South Korea (Figure 6A). Thus, the body size difference between European populations and South Korea–Japan populations could possibly be explained by the difference in major habitat types, particularly for South Korean populations.

### 4.2. Sequence Variation and Phylogenetic Relationships

The three sequence fragments from both mitochondrial and nuclear DNA showed an absence of divergent haplotypes (Figure S2), particularly in South Korea, from which more thorough sampling was performed than any other countries (forty-seven individuals collected in five populations). In particular, MSD detected in current study and public data (2.00% in 65 haplotypes in *COI*, 1.38% in 19 haplotypes in *16S rRNA*, and 1.61% in 56 haplotypes in ITS region) did not bring about any suspicion enough results to consider the estimate stemmed from two mixed species, particularly considering available studies on dragonflies, which also used homologous sequence fragments. For example, the sequence divergence of *Libellula quadrimaculata* collected from 22 localities across its range in Europe, Asia, and North America (USA and Canada) showed a sequence divergence of 0.2–2.3% between samples within North America and 1–6% between samples from different continents in *COI* [56]. Consistent observation has also been reported in other dragonfly species such as several *Sympetrum* species in *COI* and both *COI* and ITS [57,58] and *Orthetrum pruinosum* in the three sequences [59]. Moreover, phylogenetic analyses each using the concatenated sequences of two mitochondrial genes and ITS region also supported each a single large genetic group, without any separable haplotype or haplotype group, although the ITS region provided two slightly distinct subgroups in the network (Figure 7, Figure 8, Figure 9 and Figure 10, Figures S3 and S4). Previously, Sawabe et al. [19] also found a similar result, although only *16S rRNA* (378 bp) was examined from the samples collected in South Korea and Japan, for which a thorough sampling was performed.

### 4.3. Haplotype Distribution

To further scrutinize the taxonomic identity of *Sympetrum* spp., the patterns of haplotype diversification were assessed using network analysis. It has been theorized that, under a simple isolation-by-distance model, the distribution pattern of mitochondrial DNA lineages is proportional to their age [60] such that the oldest are most widespread, whereas their progeny are expected to exist closer to the areas from which they originated [61]. Consequently, the network can arrange the oldest haplotypes in the center of the network, while placing those with limited distribution (e.g., a single country) in positions derived from the oldest haplotypes, creating a star-like phylogeny.

By applying this inference to the concatenated sequences of the two mitochondrial genes, we speculated that the oldest haplotype of *Sympetrum* spp., SMT12, is likely the primary source of haplotype diversification. This is supported by its central placement within the major star-like phylogeny, from which the highest number of haplotypes originated (Figure 9). However, the complexity of the arrangement and the presence of interconnected haplotypes have resulted in a complex pattern, making a definitive inference on haplotype diversification challenging. Nevertheless, these results suggest that the expansion of *S. depressiusculum* to the eastern Asian continent did not involve any event that led to the divergence of any haplotype or haplotype group from the central SMT12 haplotype. Possibly due to such complexity, including the interconnection among haplotypes in the network by the two mitochondrial genes, BAPS analysis suggested the optimal *K* = 1, indicating the presence of only a single haplogroup in all studied countries (green, Figure 11A).

In contrast to the mitochondrial DNA network, the nuclear ITS-based network revealed two distinguishable star-like phylogenies, although they are directly interconnected by a single intermediate haplotype found exclusively in South Korea (SITS23). This intermediate haplotype shares a proximity of only 0.13% (1 base pair) with each central haplotype (Figure 10). Notably, our findings revealed variations in the composition of each group in terms of country representation (Figure 10): one group, stemming from SITS01, predominantly encompasses haplotypes from South Korea and Japan, with a smaller representation from Russia, and no haplotypes from The Netherlands. The other group, originating from SITS10, comprises all haplotypes from The Netherlands, a majority from Russia, a significant number from South Korea, and a sole representative from Japan. The ITS-based BAPS analysis further reflects this network configuration. In this regard, The Netherlands is associated with a single haplogroup (green), aligning with the SITS10-derived group within the network. Meanwhile, Russia is predominantly characterized by the green haplogroup from the SITS10-derived group, accompanied by a minority presence of the red haplogroup from the SITS01-derived group within the network. Moreover, both South Korea and Japan are predominantly linked to the red haplogroup, accompanied by a minority presence of the green haplogroup. This distribution indicates that the red haplogroup exhibits a clinal variation, ranging from being absent in The Netherlands to being more prevalent in South Korea and Japan (Figure 11B). The absence of the red haplogroup (or SITS01-derived haplotypes in the network) in The Netherlands may have been caused by incomplete sampling, possibly along with the rarity in Europe, including The Netherlands. Alternatively, the red haplogroup may have evolved from the intermediate haplotype SITS23, which connects the two central haplotypes independently in the eastern Asian continent. However, further sampling, at least for European populations, may be needed to answer this question. More importantly, it is interesting to ask why nuclear and mitochondrial genomes showed different patterns of haplotype diversification.

We interpreted the slight yet noticeable distinction observed solely in the ITS-based analysis as arising from differences in the mode of inheritance between the two types of DNA. Mitochondrial DNA is maternally inherited, whereas the ITS is a nuclear DNA segment inherited via both sexes. Consequently, the fixation process within the mitochondrial gene pool tends to be more rapid, leading to an immediate response. In contrast, the nuclear ITS region demonstrates a more gradual response due to its four-fold larger effective population size [62,63]. Thus, in instances of successful long-distance dispersal, a female carrying its mitochondrial genome could significantly influence gene pools if its offspring thrive in the new environments. This phenomenon might explain the prevalence of the European gene pool extending to the eastern Asian continent, as indicated by the mitochondrial DNA-based BAPS analyses (Figure 11A). Conversely, a male carrying its nuclear genome would exert a lesser influence on gene pools in new sites following successful mating. This implies that the dominant gene pool in Europe would have a relatively limited impact on populations on the eastern Asian continent, leading to a noticeable differentiation in gene pools between Europe, including The Netherlands, and the eastern Asian continent. Therefore, this observation can be understood within the context of the differing modes of inheritance between the two types of genetic material and the diversification patterns within a species, while also considering that other data analyzed in this study do not support such a subdivision.

*S. depressiusculum* boasts a wide distribution spanning from Japan and the Korean Peninsula through northeastern China and southern Siberia to Western Europe, including France [53,64]. It is also important to note that our study primarily focused on samples from South Korea, with only a limited representation from Russia. Additionally, samples covering the entire Palearctic range were lacking. Consequently, a more extensive collection of samples from diverse regions would be essential to establish a robust inference concerning the patterns of haplotype diversification within mitochondrial and genomic DNA, as they relate to the species status of both *S. depressiusculum* and *S. frequens*.

## 5. Conclusions

South Korean populations resemble European *S. depressiusculum* in some morphological characters that have been employed to distinguish between European *S. depressiusculum* and Japanese *S. frequens*, with a subtle difference in genitalia in both sexes. However, South Korean *S. depressiusculum* more so resemble *S. frequens* in body size, which may reflect a difference in major habitat type between Europe and South Korea. Further, four individuals collected in a west–central region in South Korea were most likely *S. depressiusculum* in terms of their overall morphology and size. These observations suggest that there is no support for the presence of two *Sympetrum* species in South Korea. Phylogenetic analyses using data from three genomic regions that were obtained in this study and from public archives further support this observation. These analyses provided a single large clade within all geographic samples that lacked any discernable subgroups and lacked country-specific or divergent haplotypes within the clade. Network analysis based only on ITS region provided two slightly distinct groups, with one being dominant in The Netherlands–Russia and the other being dominant in South Korea–Japan. However, it is important to note that this observation is likely attributable to within-species diversification patterns rather than speciation. Our study sought to understand the species status of South Korean populations among neighboring populations, but we unexpectedly observed the potential of *S. depressiusculum* and *S. frequens* to be a single species.

## Data Availability

The data presented in this study are available in the text and Supplementary Material here.

## Supplementary Materials

The following supporting information can be downloaded at: https://www.mdpi.com/article/10.3390/insects14090733/s1, Figure S1: Chromatograms showing individuals with dimorphic sites in ITS sequences; Figure S2: Distribution of pairwise distances in base pairs; Figure S3: Phylogeny of *Sympetrum COI* + *16S rRNA* haplotypes obtained from the current study and public data using the maximum likelihood method; Figure S4: Phylogeny of *Sympetrum* ITS haplotypes obtained from the current study and public data using the maximum likelihood method; Table S1: Collection sites of *Sympetrum* specimens; Table S2: Primers utilized to amplify and sequence *COI*, *16S rRNA*, and the ITS region; Table S3: List of *Sympetrum* species samples sequenced in this study and obtained from public data; Table S4: Relative frequencies of *COI* haplotypes of *Sympetrum* species sequenced in this study and collected from public data; Table S5: Relative frequencies of *16S rRNA* haplotypes of *Sympetrum* species sequenced in this study and collected from public data; Table S6: Relative frequencies of *COI* + *16S rRNA* haplotypes of *Sympetrum* species sequenced in this study and collected from public data; Table S7: Relative frequencies of ITS haplotypes of *Sympetrum* species sequenced in this study and collected from public data; Table S8: Pairwise comparisons of *COI* haplotypes of *Sympetrum* species sequenced in this study and collected from public data; Table S9: Pairwise comparisons of *16S rRNA* haplotypes of *Sympetrum* species sequenced in this study and collected from public data; Table S10: Pairwise comparisons of *COI* + *16S rRNA* haplotypes of *Sympetrum* species sequenced in this study and collected from public data; Table S11: Pairwise comparisons of ITS haplotypes of *Sympetrum* species sequenced in this study and collected from public data. References [24,25,26,27,28,29] are cited in both the main text and Supplementary Materials.
