# Supplementary material for: Identification of Sympetrum depressiusculum Sélys, 1841 in South Korea (Odonata: Libellulidae) According to Morphology and Genetic Markers
Source: Insects. 2023 Aug 30;14(9):733. doi: 10.3390/insects14090733 (PMC10531817; doi:10.3390/insects14090733)
Supplement: Supplementary file 1 [file insects-14-00733-s001.zip › Figure S1. ITS dimorphic sites.pptx]

## Slide 1
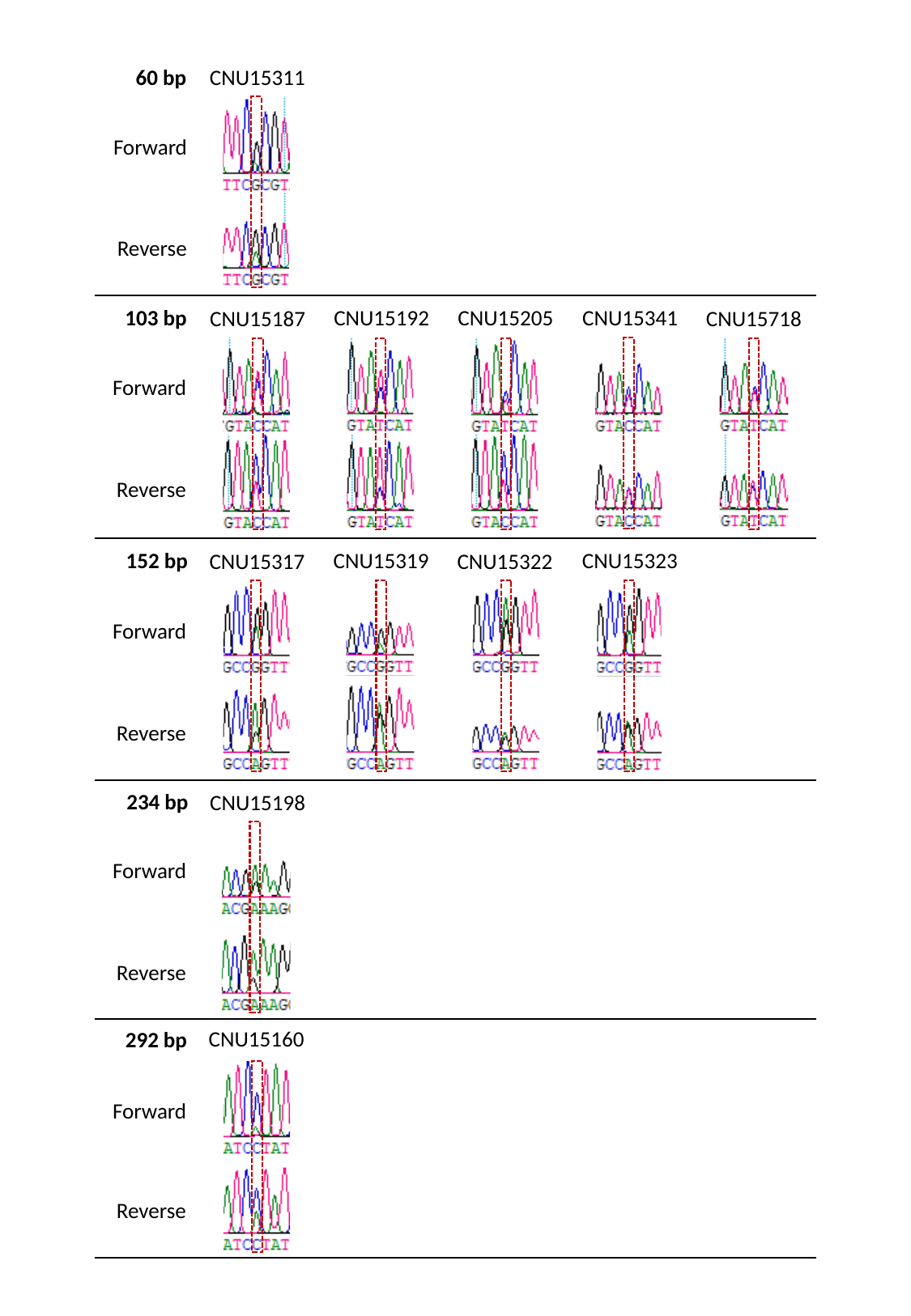

CNU15311
60 bp
Forward
Reverse
103 bp
CNU15192
CNU15205
CNU15341
CNU15187
CNU15718
152 bp
CNU15319
CNU15323
CNU15317
CNU15322
234 bp
CNU15198
CNU15160
292 bp
Forward
Reverse
Forward
Reverse
Forward
Reverse
Forward
Reverse

## Slide 2
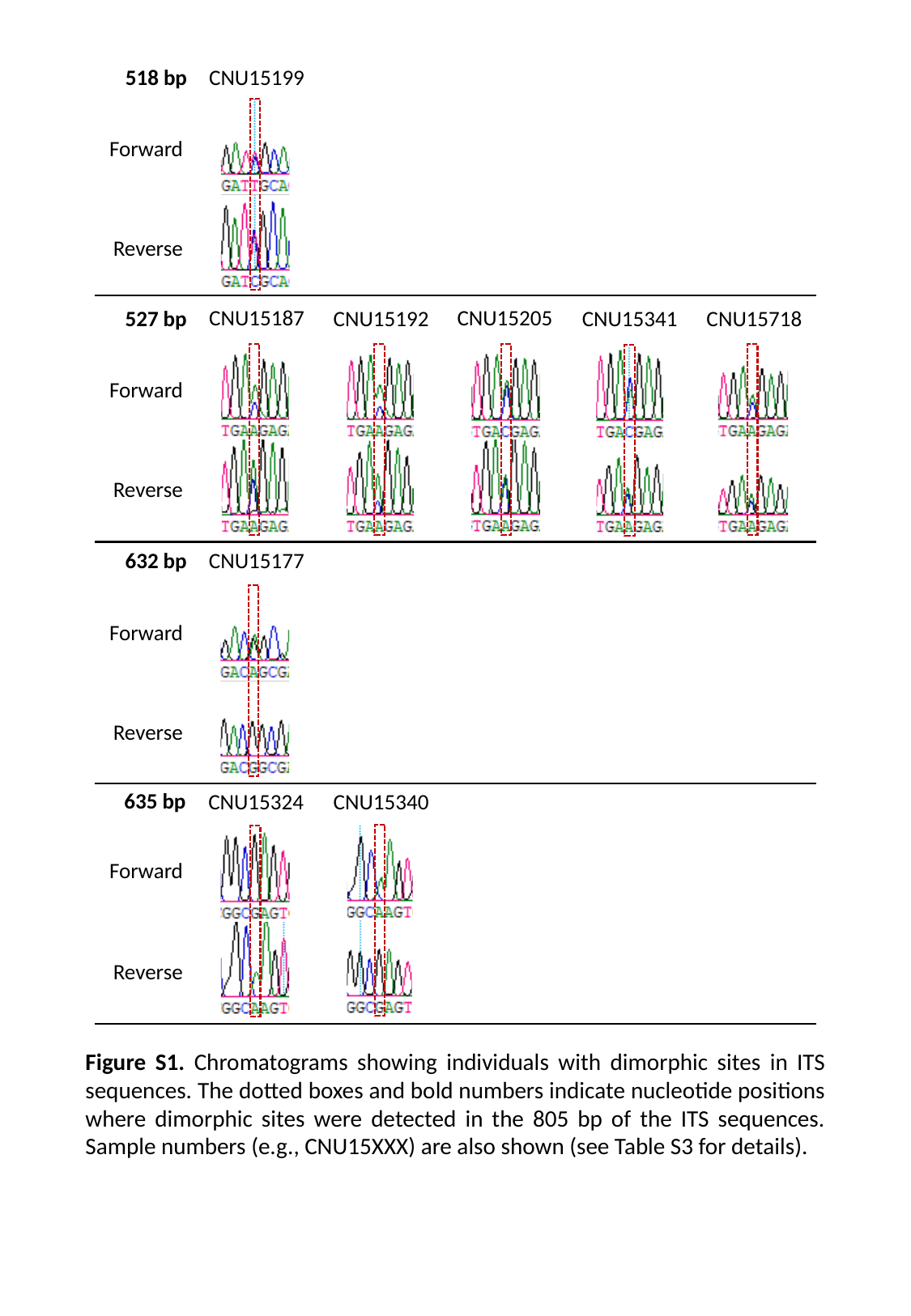

CNU15199
518 bp
CNU15187
CNU15205
CNU15341
CNU15192
CNU15718
527 bp
632 bp
CNU15177
635 bp
CNU15324
CNU15340
Forward
Reverse
Forward
Reverse
Forward
Reverse
Forward
Reverse
Figure S1. Chromatograms showing individuals with dimorphic sites in ITS sequences. The dotted boxes and bold numbers indicate nucleotide positions where dimorphic sites were detected in the 805 bp of the ITS sequences. Sample numbers (e.g., CNU15XXX) are also shown (see Table S3 for details).
