# Supplementary material for: Identification of Sympetrum depressiusculum Sélys, 1841 in South Korea (Odonata: Libellulidae) According to Morphology and Genetic Markers
Source: Insects. 2023 Aug 30;14(9):733. doi: 10.3390/insects14090733 (PMC10531817; doi:10.3390/insects14090733)
Supplement: Supplementary file 1 [file insects-14-00733-s001.zip › Figure S2. Haplotype frequency.pptx]

## Slide 1
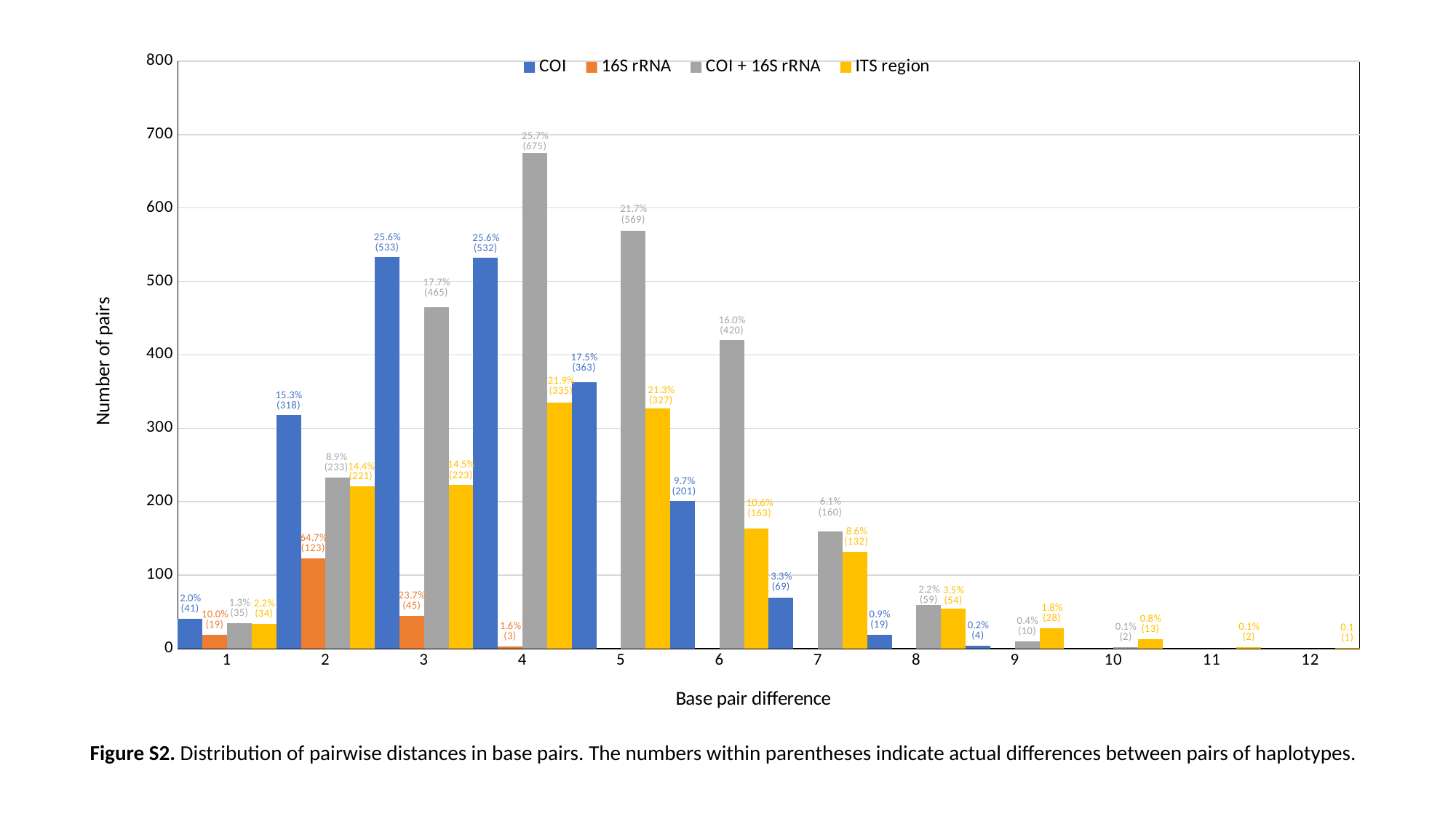

### Chart:
| Category | COI | 16S rRNA | COI + 16S rRNA | ITS region |
|---|---|---|---|---|
| 1 | 41.0 | 19.0 | 35.0 | 34.0 |
| 2 | 318.0 | 123.0 | 233.0 | 221.0 |
| 3 | 533.0 | 45.0 | 465.0 | 223.0 |
| 4 | 532.0 | 3.0 | 675.0 | 335.0 |
| 5 | 363.0 | None | 569.0 | 327.0 |
| 6 | 201.0 | None | 420.0 | 163.0 |
| 7 | 69.0 | None | 160.0 | 132.0 |
| 8 | 19.0 | None | 59.0 | 54.0 |
| 9 | 4.0 | None | 10.0 | 28.0 |
| 10 | None | None | 2.0 | 13.0 |
| 11 | None | None | None | 2.0 |
| 12 | None | None | None | 1.0 |Figure S2. Distribution of pairwise distances in base pairs. The numbers within parentheses indicate actual differences between pairs of haplotypes.
