# Supplementary material for: Identification of Sympetrum depressiusculum Sélys, 1841 in South Korea (Odonata: Libellulidae) According to Morphology and Genetic Markers
Source: Insects. 2023 Aug 30;14(9):733. doi: 10.3390/insects14090733 (PMC10531817; doi:10.3390/insects14090733)
Supplement: Supplementary file 1 [file insects-14-00733-s001.zip › Figure S3. COI+16S rRNA ML.pptx]

## Slide 1
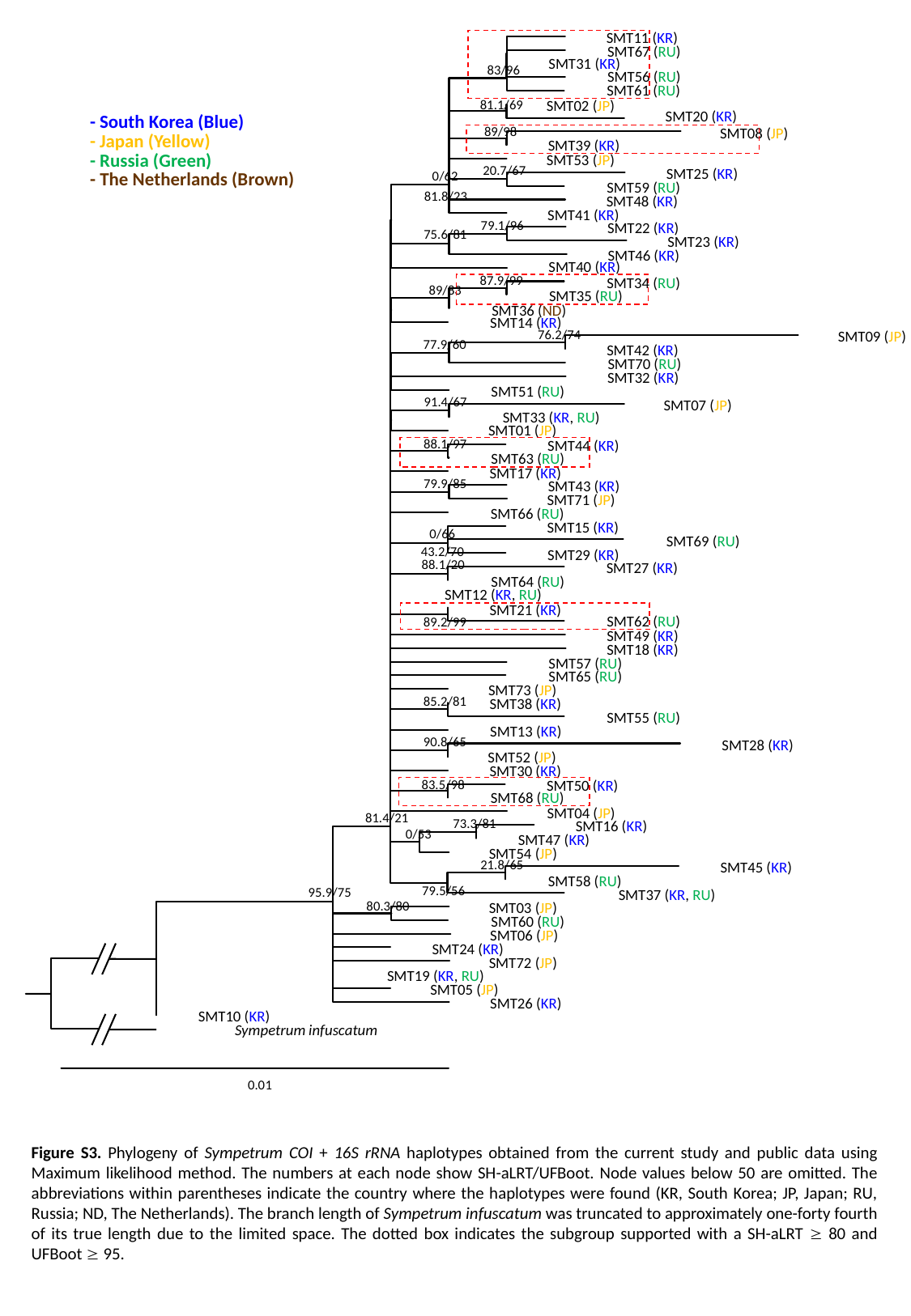

SMT11 (KR)
SMT67 (RU)
SMT31 (KR)
SMT56 (RU)
SMT61 (RU)
SMT02 (JP)
SMT20 (KR)
SMT08 (JP)
SMT39 (KR)
SMT53 (JP)
SMT25 (KR)
SMT59 (RU)
SMT48 (KR)
SMT41 (KR)
SMT22 (KR)
SMT23 (KR)
SMT46 (KR)
SMT40 (KR)
SMT34 (RU)
SMT35 (RU)
SMT36 (ND)
SMT14 (KR)
SMT09 (JP)
SMT42 (KR)
SMT70 (RU)
SMT32 (KR)
SMT51 (RU)
SMT07 (JP)
SMT33 (KR, RU)
SMT01 (JP)
SMT44 (KR)
SMT63 (RU)
SMT17 (KR)
SMT43 (KR)
SMT71 (JP)
SMT66 (RU)
SMT15 (KR)
SMT69 (RU)
SMT29 (KR)
SMT27 (KR)
SMT64 (RU)
SMT12 (KR, RU)
SMT21 (KR)
SMT62 (RU)
SMT49 (KR)
SMT18 (KR)
SMT57 (RU)
SMT65 (RU)
SMT73 (JP)
SMT38 (KR)
SMT55 (RU)
SMT13 (KR)
SMT28 (KR)
SMT52 (JP)
SMT30 (KR)
SMT50 (KR)
SMT68 (RU)
SMT04 (JP)
SMT16 (KR)
SMT47 (KR)
SMT54 (JP)
SMT45 (KR)
SMT58 (RU)
SMT37 (KR, RU)
SMT03 (JP)
SMT60 (RU)
SMT06 (JP)
SMT24 (KR)
SMT72 (JP)
SMT19 (KR, RU)
SMT05 (JP)
SMT26 (KR)
SMT10 (KR)
Sympetrum infuscatum
0.01
83/96
81.1/69
89/98
20.7/67
0/62
81.8/23
79.1/96
75.6/81
87.9/99
89/83
76.2/74
77.9/60
91.4/67
88.1/97
79.9/85
0/66
43.2/70
88.1/20
89.2/99
85.2/81
90.8/65
83.5/98
81.4/21
73.3/81
0/53
21.8/65
79.5/56
95.9/75
80.3/80
- South Korea (Blue)
- Japan (Yellow)
- Russia (Green)
- The Netherlands (Brown)
Figure S3. Phylogeny of Sympetrum COI + 16S rRNA haplotypes obtained from the current study and public data using Maximum likelihood method. The numbers at each node show SH-aLRT/UFBoot. Node values below 50 are omitted. The abbreviations within parentheses indicate the country where the haplotypes were found (KR, South Korea; JP, Japan; RU, Russia; ND, The Netherlands). The branch length of Sympetrum infuscatum was truncated to approximately one-forty fourth of its true length due to the limited space. The dotted box indicates the subgroup supported with a SH-aLRT ≥ 80 and UFBoot ≥ 95.
