# Supplementary material for: Identification of Sympetrum depressiusculum Sélys, 1841 in South Korea (Odonata: Libellulidae) According to Morphology and Genetic Markers
Source: Insects. 2023 Aug 30;14(9):733. doi: 10.3390/insects14090733 (PMC10531817; doi:10.3390/insects14090733)
Supplement: Supplementary file 1 [file insects-14-00733-s001.zip › Figure S4. ITS ML.pptx]

## Slide 1
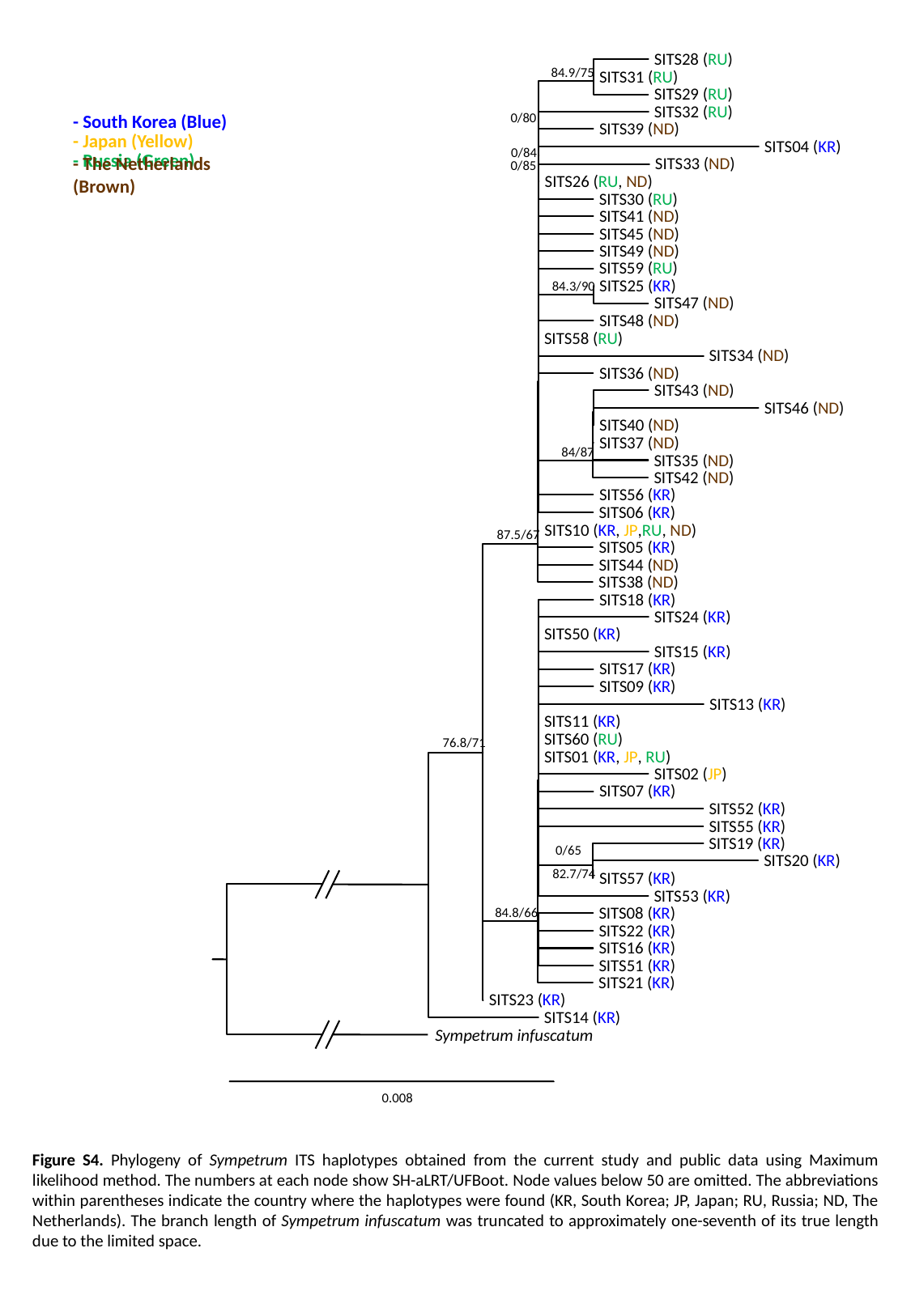

SITS28 (RU)
SITS31 (RU)
SITS29 (RU)
SITS32 (RU)
SITS39 (ND)
SITS04 (KR)
SITS33 (ND)
SITS26 (RU, ND)
SITS30 (RU)
SITS41 (ND)
SITS45 (ND)
SITS49 (ND)
SITS59 (RU)
SITS25 (KR)
SITS47 (ND)
SITS48 (ND)
SITS58 (RU)
SITS34 (ND)
SITS36 (ND)
SITS43 (ND)
SITS46 (ND)
SITS40 (ND)
SITS37 (ND)
SITS35 (ND)
SITS42 (ND)
SITS56 (KR)
SITS06 (KR)
SITS10 (KR, JP,RU, ND)
SITS05 (KR)
SITS44 (ND)
SITS38 (ND)
SITS18 (KR)
SITS24 (KR)
SITS50 (KR)
SITS15 (KR)
SITS17 (KR)
SITS09 (KR)
SITS13 (KR)
SITS11 (KR)
SITS60 (RU)
SITS01 (KR, JP, RU)
SITS02 (JP)
SITS07 (KR)
SITS52 (KR)
SITS55 (KR)
SITS19 (KR)
SITS20 (KR)
SITS57 (KR)
SITS53 (KR)
SITS08 (KR)
SITS22 (KR)
SITS16 (KR)
SITS51 (KR)
SITS21 (KR)
SITS23 (KR)
SITS14 (KR)
Sympetrum infuscatum
84.9/75
0/80
0/84
0/85
84.3/90
84/87
87.5/67
76.8/71
0/65
82.7/74
84.8/66
- South Korea (Blue)
- Japan (Yellow)
- Russia (Green)
- The Netherlands (Brown)
0.008
Figure S4. Phylogeny of Sympetrum ITS haplotypes obtained from the current study and public data using Maximum likelihood method. The numbers at each node show SH-aLRT/UFBoot. Node values below 50 are omitted. The abbreviations within parentheses indicate the country where the haplotypes were found (KR, South Korea; JP, Japan; RU, Russia; ND, The Netherlands). The branch length of Sympetrum infuscatum was truncated to approximately one-seventh of its true length due to the limited space.
