# Supplementary material for: Identification of Sympetrum depressiusculum Sélys, 1841 in South Korea (Odonata: Libellulidae) According to Morphology and Genetic Markers
Source: Insects. 2023 Aug 30;14(9):733. doi: 10.3390/insects14090733 (PMC10531817; doi:10.3390/insects14090733)
Supplement: Supplementary file 1 [file insects-14-00733-s001.zip › Table S1. Collection site.docx]

**Table S1.** Collection sites of *Sympetrum* specimens.

| Country | Site | Sample size | Collection date | Coordinates |
| --- | --- | --- | --- | --- |
| 1. South Korea | Inje, Gangwon-do | 10 | 03.09.2021 | 38°04'39.0"N, 128°10'35.0"E |
| 2. South Korea | Gyoha-dong, Paju, Gyeonggi-do | 10 | 30.08.2021 | 37°45'11.9" N, 126°44'47.0" E |
| 3. South Korea | Gojan-dong, Namdong-gu, Incheon | 4 | 27.09.2021 | 37°23'37.3"N, 126°41'50.8"E |
| 4. South Korea | Boeun, Chungcheongbuk-do | 10 | 16.09.2021 | 36°29'29.0"N, 127°43'39.0"E |
| 5. South Korea | Jeongseon, Gangwon-do | 13 | 26.09.2022 | 37°20'28.9"N 128°38'53.8"E |
| 6. Japan | Tamagawacho Shitagiri, Takahashi, Okayama | 10 | 30.07.2021 | 34°46'09.1"N, 133°34'27.1"E |
| 7. Russia | Ordynsky District, Novosibirsk region | 2 | 05.08.2021 | 54°08'48.8"N, 81°36'37.1"E |
| 7. Russia | Babayurtovsky District, Republic of Dagestan | 3 | 01.07.2021 | 43°41'30.5"N, 47°14'45.2"E |
| 8. The Netherlands | Scheerwolde, Overijssel | 12 | 26.08.2021 | 52°47'17.2"N, 6°01'34.0"E |
