# Supplementary material for: Identification of Sympetrum depressiusculum Sélys, 1841 in South Korea (Odonata: Libellulidae) According to Morphology and Genetic Markers
Source: Insects. 2023 Aug 30;14(9):733. doi: 10.3390/insects14090733 (PMC10531817; doi:10.3390/insects14090733)
Supplement: Supplementary file 1 [file insects-14-00733-s001.zip › Table S10. PC-Our COI+16S+GB COI+16S.docx]

**Table S10.** Pairwise comparisons of *COI* + *16S rRNA* haplotypes of *Sympetrum* species sequenced in this study and collected from public data.

| Haplotype | 1 | 2 | 3 | 4 | 5 | 6 | 7 | 8 | 9 | 10 | 11 | 12 | 13 | 14 | 15 |
| --- | --- | --- | --- | --- | --- | --- | --- | --- | --- | --- | --- | --- | --- | --- | --- |
| 1. SMT01 | - | 0.41 | 0.55 | 0.41 | 0.41 | 0.55 | 0.69 | 0.83 | 0.83 | 0.69 | 0.55 | 0.14 | 0.28 | 0.28 | 0.41 |
| 2. SMT02 | 3 | - | 0.41 | 0.55 | 0.28 | 0.41 | 0.83 | 0.69 | 0.69 | 0.55 | 0.41 | 0.28 | 0.41 | 0.41 | 0.55 |
| 3. SMT03 | 4 | 3 | - | 0.55 | 0.41 | 0.55 | 0.96 | 1.10 | 1.10 | 0.69 | 0.83 | 0.41 | 0.55 | 0.55 | 0.69 |
| 4. SMT04 | 3 | 4 | 4 | - | 0.55 | 0.69 | 0.83 | 0.96 | 0.96 | 0.83 | 0.69 | 0.28 | 0.41 | 0.41 | 0.55 |
| 5. SMT05 | 3 | 2 | 3 | 4 | - | 0.41 | 0.83 | 0.96 | 0.96 | 0.55 | 0.69 | 0.28 | 0.41 | 0.41 | 0.55 |
| 6. SMT06 | 4 | 3 | 4 | 5 | 3 | - | 0.96 | 1.10 | 1.10 | 0.69 | 0.83 | 0.41 | 0.55 | 0.55 | 0.69 |
| 7. SMT07 | 5 | 6 | 7 | 6 | 6 | 7 | - | 1.24 | 1.24 | 1.10 | 0.96 | 0.55 | 0.69 | 0.69 | 0.83 |
| 8. SMT08 | 6 | 5 | 8 | 7 | 7 | 8 | 9 | - | 1.10 | 1.24 | 0.83 | 0.69 | 0.83 | 0.83 | 0.96 |
| 9. SMT09 | 6 | 5 | 8 | 7 | 7 | 8 | 9 | 8 | - | 1.24 | 0.83 | 0.69 | 0.83 | 0.83 | 0.96 |
| 10. SMT10 | 5 | 4 | 5 | 6 | 4 | 5 | 8 | 9 | 9 | - | 0.96 | 0.55 | 0.69 | 0.69 | 0.83 |
| 11. SMT11 | 4 | 3 | 6 | 5 | 5 | 6 | 7 | 6 | 6 | 7 | - | 0.41 | 0.55 | 0.55 | 0.69 |
| 12. SMT12 | 1 | 2 | 3 | 2 | 2 | 3 | 4 | 5 | 5 | 4 | 3 | - | 0.14 | 0.14 | 0.28 |
| 13. SMT13 | 2 | 3 | 4 | 3 | 3 | 4 | 5 | 6 | 6 | 5 | 4 | 1 | - | 0.28 | 0.41 |
| 14. SMT14 | 2 | 3 | 4 | 3 | 3 | 4 | 5 | 6 | 6 | 5 | 4 | 1 | 2 | - | 0.41 |
| 15. SMT15 | 3 | 4 | 5 | 4 | 4 | 5 | 6 | 7 | 7 | 6 | 5 | 2 | 3 | 3 | - |
| 16. SMT16 | 3 | 4 | 5 | 4 | 4 | 5 | 4 | 7 | 7 | 6 | 5 | 2 | 3 | 3 | 4 |
| 17. SMT17 | 2 | 3 | 4 | 3 | 3 | 4 | 5 | 6 | 6 | 5 | 4 | 1 | 2 | 2 | 3 |
| 18. SMT18 | 4 | 5 | 4 | 5 | 5 | 6 | 7 | 8 | 8 | 7 | 6 | 3 | 4 | 4 | 5 |
| 19. SMT19 | 2 | 1 | 2 | 3 | 1 | 2 | 5 | 6 | 6 | 3 | 4 | 1 | 2 | 2 | 3 |
| 20. SMT20 | 5 | 2 | 5 | 6 | 4 | 5 | 8 | 7 | 7 | 6 | 4 | 4 | 5 | 5 | 6 |
| 21. SMT21 | 2 | 3 | 4 | 3 | 3 | 4 | 5 | 6 | 6 | 5 | 4 | 1 | 2 | 2 | 3 |
| 22. SMT22 | 4 | 5 | 6 | 5 | 3 | 6 | 7 | 8 | 6 | 7 | 6 | 3 | 4 | 4 | 5 |
| 23. SMT23 | 5 | 6 | 7 | 6 | 4 | 7 | 6 | 9 | 7 | 8 | 7 | 4 | 5 | 5 | 6 |
| 24. SMT24 | 3 | 2 | 3 | 4 | 2 | 3 | 6 | 7 | 7 | 4 | 5 | 2 | 3 | 3 | 4 |
| 25. SMT25 | 5 | 4 | 7 | 6 | 6 | 7 | 6 | 7 | 7 | 6 | 5 | 4 | 5 | 5 | 6 |
| 26. SMT26 | 4 | 3 | 4 | 5 | 3 | 4 | 7 | 8 | 8 | 5 | 6 | 3 | 4 | 4 | 5 |
| 27. SMT27 | 4 | 5 | 6 | 5 | 5 | 6 | 5 | 8 | 8 | 7 | 6 | 3 | 4 | 4 | 5 |
| 28. SMT28 | 6 | 5 | 6 | 7 | 5 | 6 | 7 | 10 | 10 | 7 | 8 | 5 | 6 | 6 | 7 |
| 29. SMT29 | 3 | 4 | 5 | 4 | 4 | 5 | 4 | 7 | 7 | 6 | 5 | 2 | 3 | 3 | 2 |
| 30. SMT30 | 2 | 3 | 4 | 3 | 3 | 4 | 5 | 6 | 6 | 5 | 2 | 1 | 2 | 2 | 3 |
| 31. SMT31 | 3 | 2 | 5 | 4 | 4 | 5 | 6 | 5 | 5 | 6 | 1 | 2 | 3 | 3 | 4 |
| 32. SMT32 | 4 | 5 | 6 | 5 | 5 | 6 | 7 | 6 | 8 | 5 | 6 | 3 | 4 | 4 | 5 |
| 33. SMT33 | 2 | 3 | 4 | 3 | 3 | 4 | 3 | 6 | 6 | 5 | 4 | 1 | 2 | 2 | 3 |
| 34. SMT34 | 4 | 5 | 6 | 5 | 5 | 6 | 7 | 8 | 8 | 7 | 4 | 3 | 4 | 4 | 5 |
| 35. SMT35 | 3 | 4 | 5 | 4 | 4 | 5 | 6 | 7 | 7 | 6 | 3 | 2 | 3 | 3 | 4 |
| 36. SMT36 | 2 | 3 | 4 | 3 | 3 | 4 | 5 | 6 | 6 | 5 | 2 | 1 | 2 | 2 | 3 |
| 37. SMT37 | 4 | 5 | 6 | 5 | 5 | 5 | 7 | 8 | 8 | 7 | 6 | 3 | 4 | 4 | 5 |
| 38. SMT38 | 2 | 3 | 4 | 3 | 3 | 4 | 5 | 6 | 6 | 5 | 4 | 1 | 2 | 2 | 3 |
| 39. SMT39 | 3 | 2 | 5 | 4 | 4 | 5 | 6 | 3 | 5 | 6 | 3 | 2 | 3 | 3 | 4 |
| 40. SMT40 | 3 | 4 | 5 | 4 | 4 | 5 | 6 | 7 | 7 | 6 | 5 | 2 | 3 | 3 | 4 |
| 41. SMT41 | 3 | 2 | 5 | 4 | 4 | 5 | 6 | 5 | 5 | 6 | 3 | 2 | 3 | 3 | 4 |
| 42. SMT42 | 4 | 5 | 6 | 5 | 5 | 6 | 7 | 8 | 4 | 7 | 6 | 3 | 4 | 4 | 5 |
| 43. SMT43 | 3 | 4 | 5 | 4 | 4 | 5 | 6 | 7 | 7 | 6 | 5 | 2 | 3 | 3 | 4 |
| 44. SMT44 | 3 | 4 | 5 | 4 | 4 | 5 | 6 | 5 | 7 | 6 | 5 | 2 | 3 | 3 | 4 |
| 45. SMT45 | 4 | 5 | 6 | 5 | 5 | 6 | 7 | 8 | 8 | 7 | 6 | 3 | 4 | 4 | 5 |
| 46. SMT46 | 4 | 5 | 6 | 5 | 3 | 6 | 5 | 8 | 8 | 7 | 6 | 3 | 4 | 4 | 5 |
| 47. SMT47 | 2 | 3 | 4 | 3 | 3 | 4 | 5 | 6 | 6 | 5 | 4 | 1 | 2 | 2 | 3 |
| 48. SMT48 | 4 | 3 | 6 | 5 | 5 | 6 | 7 | 6 | 6 | 5 | 4 | 3 | 4 | 4 | 5 |
| 49. SMT49 | 4 | 5 | 6 | 5 | 5 | 6 | 7 | 8 | 8 | 7 | 6 | 3 | 4 | 4 | 5 |
| 50. SMT50 | 3 | 4 | 5 | 4 | 4 | 5 | 6 | 7 | 7 | 6 | 5 | 2 | 3 | 3 | 4 |
| 51. SMT51 | 2 | 3 | 4 | 3 | 3 | 4 | 5 | 6 | 6 | 5 | 4 | 1 | 2 | 2 | 3 |
| 52. SMT52 | 2 | 3 | 4 | 3 | 3 | 4 | 5 | 6 | 6 | 5 | 4 | 1 | 2 | 2 | 3 |
| 53. SMT53 | 3 | 2 | 5 | 4 | 4 | 3 | 6 | 5 | 5 | 6 | 3 | 2 | 3 | 3 | 4 |
| 54. SMT54 | 2 | 3 | 4 | 3 | 3 | 4 | 5 | 6 | 6 | 5 | 4 | 1 | 2 | 2 | 3 |
| 55. SMT55 | 4 | 5 | 6 | 5 | 5 | 6 | 7 | 6 | 8 | 7 | 6 | 3 | 4 | 4 | 5 |
| 56. SMT56 | 4 | 3 | 6 | 5 | 5 | 6 | 5 | 6 | 6 | 7 | 2 | 3 | 4 | 4 | 5 |
| 57. SMT57 | 3 | 4 | 5 | 4 | 4 | 5 | 6 | 7 | 7 | 6 | 5 | 2 | 3 | 3 | 4 |
| 58. SMT58 | 3 | 4 | 5 | 4 | 4 | 5 | 6 | 7 | 7 | 6 | 5 | 2 | 3 | 3 | 4 |
| 59. SMT59 | 4 | 3 | 6 | 5 | 5 | 6 | 7 | 6 | 6 | 7 | 4 | 3 | 4 | 4 | 5 |
| 60. SMT60 | 4 | 3 | 2 | 5 | 3 | 4 | 7 | 8 | 8 | 5 | 6 | 3 | 4 | 4 | 5 |
| 61. SMT61 | 4 | 3 | 6 | 5 | 5 | 6 | 7 | 6 | 6 | 7 | 2 | 3 | 4 | 4 | 5 |
| 62. SMT62 | 4 | 5 | 6 | 5 | 5 | 6 | 7 | 8 | 8 | 7 | 6 | 3 | 4 | 4 | 5 |
| 63. SMT63 | 2 | 3 | 4 | 3 | 3 | 4 | 5 | 6 | 6 | 5 | 4 | 1 | 2 | 2 | 3 |
| 64. SMT64 | 2 | 3 | 4 | 3 | 3 | 4 | 3 | 6 | 6 | 5 | 4 | 1 | 2 | 2 | 3 |
| 65. SMT65 | 3 | 4 | 5 | 4 | 4 | 5 | 6 | 7 | 7 | 6 | 5 | 2 | 3 | 3 | 4 |
| 66. SMT66 | 2 | 3 | 4 | 3 | 3 | 4 | 5 | 6 | 6 | 5 | 4 | 1 | 2 | 2 | 3 |
| 67. SMT67 | 4 | 3 | 6 | 5 | 5 | 6 | 7 | 6 | 6 | 7 | 2 | 3 | 4 | 4 | 5 |
| 68. SMT68 | 2 | 3 | 4 | 3 | 3 | 4 | 5 | 6 | 6 | 5 | 4 | 1 | 2 | 2 | 3 |
| 69. SMT69 | 5 | 6 | 7 | 6 | 6 | 7 | 8 | 9 | 9 | 8 | 7 | 4 | 5 | 5 | 4 |
| 70. SMT70 | 4 | 5 | 6 | 5 | 5 | 6 | 7 | 8 | 8 | 7 | 6 | 3 | 4 | 4 | 5 |
| 71. SMT71 | 3 | 4 | 5 | 4 | 4 | 5 | 6 | 7 | 7 | 6 | 5 | 2 | 3 | 3 | 4 |
| 72. SMT72 | 4 | 3 | 4 | 5 | 3 | 4 | 7 | 8 | 8 | 5 | 6 | 3 | 4 | 4 | 5 |
| 73. SMT73 | 2 | 3 | 4 | 3 | 3 | 4 | 5 | 6 | 6 | 5 | 4 | 1 | 2 | 2 | 3 |

| Haplotype | 16 | 17 | 18 | 19 | 20 | 21 | 22 | 23 | 24 | 25 | 26 | 27 | 28 | 29 | 30 |
| --- | --- | --- | --- | --- | --- | --- | --- | --- | --- | --- | --- | --- | --- | --- | --- |
| 1. SMT01 | 0.41 | 0.28 | 0.55 | 0.28 | 0.69 | 0.28 | 0.55 | 0.69 | 0.41 | 0.69 | 0.55 | 0.55 | 0.83 | 0.41 | 0.28 |
| 2. SMT02 | 0.55 | 0.41 | 0.69 | 0.14 | 0.28 | 0.41 | 0.69 | 0.83 | 0.28 | 0.55 | 0.41 | 0.69 | 0.69 | 0.55 | 0.41 |
| 3. SMT03 | 0.69 | 0.55 | 0.55 | 0.28 | 0.69 | 0.55 | 0.83 | 0.96 | 0.41 | 0.96 | 0.55 | 0.83 | 0.83 | 0.69 | 0.55 |
| 4. SMT04 | 0.55 | 0.41 | 0.69 | 0.41 | 0.83 | 0.41 | 0.69 | 0.83 | 0.55 | 0.83 | 0.69 | 0.69 | 0.96 | 0.55 | 0.41 |
| 5. SMT05 | 0.55 | 0.41 | 0.69 | 0.14 | 0.55 | 0.41 | 0.41 | 0.55 | 0.28 | 0.83 | 0.41 | 0.69 | 0.69 | 0.55 | 0.41 |
| 6. SMT06 | 0.69 | 0.55 | 0.83 | 0.28 | 0.69 | 0.55 | 0.83 | 0.96 | 0.41 | 0.96 | 0.55 | 0.83 | 0.83 | 0.69 | 0.55 |
| 7. SMT07 | 0.55 | 0.69 | 0.96 | 0.69 | 1.10 | 0.69 | 0.96 | 0.83 | 0.83 | 0.83 | 0.96 | 0.69 | 0.96 | 0.55 | 0.69 |
| 8. SMT08 | 0.96 | 0.83 | 1.10 | 0.83 | 0.96 | 0.83 | 1.10 | 1.24 | 0.96 | 0.96 | 1.10 | 1.10 | 1.38 | 0.96 | 0.83 |
| 9. SMT09 | 0.96 | 0.83 | 1.10 | 0.83 | 0.96 | 0.83 | 0.83 | 0.96 | 0.96 | 0.96 | 1.10 | 1.10 | 1.38 | 0.96 | 0.83 |
| 10. SMT10 | 0.83 | 0.69 | 0.96 | 0.41 | 0.83 | 0.69 | 0.96 | 1.10 | 0.55 | 0.83 | 0.69 | 0.96 | 0.96 | 0.83 | 0.69 |
| 11. SMT11 | 0.69 | 0.55 | 0.83 | 0.55 | 0.55 | 0.55 | 0.83 | 0.96 | 0.69 | 0.69 | 0.83 | 0.83 | 1.10 | 0.69 | 0.28 |
| 12. SMT12 | 0.28 | 0.14 | 0.41 | 0.14 | 0.55 | 0.14 | 0.41 | 0.55 | 0.28 | 0.55 | 0.41 | 0.41 | 0.69 | 0.28 | 0.14 |
| 13. SMT13 | 0.41 | 0.28 | 0.55 | 0.28 | 0.69 | 0.28 | 0.55 | 0.69 | 0.41 | 0.69 | 0.55 | 0.55 | 0.83 | 0.41 | 0.28 |
| 14. SMT14 | 0.41 | 0.28 | 0.55 | 0.28 | 0.69 | 0.28 | 0.55 | 0.69 | 0.41 | 0.69 | 0.55 | 0.55 | 0.83 | 0.41 | 0.28 |
| 15. SMT15 | 0.55 | 0.41 | 0.69 | 0.41 | 0.83 | 0.41 | 0.69 | 0.83 | 0.55 | 0.83 | 0.69 | 0.69 | 0.96 | 0.28 | 0.41 |
| 16. SMT16 | - | 0.41 | 0.69 | 0.41 | 0.83 | 0.41 | 0.69 | 0.83 | 0.55 | 0.83 | 0.69 | 0.41 | 0.69 | 0.28 | 0.41 |
| 17. SMT17 | 3 | - | 0.55 | 0.28 | 0.69 | 0.28 | 0.55 | 0.69 | 0.41 | 0.69 | 0.55 | 0.55 | 0.83 | 0.41 | 0.28 |
| 18. SMT18 | 5 | 4 | - | 0.55 | 0.96 | 0.55 | 0.83 | 0.96 | 0.69 | 0.96 | 0.69 | 0.83 | 1.10 | 0.69 | 0.55 |
| 19. SMT19 | 3 | 2 | 4 | - | 0.41 | 0.28 | 0.55 | 0.69 | 0.14 | 0.69 | 0.28 | 0.55 | 0.55 | 0.41 | 0.28 |
| 20. SMT20 | 6 | 5 | 7 | 3 | - | 0.69 | 0.96 | 1.10 | 0.55 | 0.83 | 0.69 | 0.96 | 0.96 | 0.83 | 0.69 |
| 21. SMT21 | 3 | 2 | 4 | 2 | 5 | - | 0.55 | 0.69 | 0.41 | 0.69 | 0.55 | 0.55 | 0.83 | 0.41 | 0.28 |
| 22. SMT22 | 5 | 4 | 6 | 4 | 7 | 4 | - | 0.41 | 0.69 | 0.96 | 0.83 | 0.83 | 1.10 | 0.69 | 0.55 |
| 23. SMT23 | 6 | 5 | 7 | 5 | 8 | 5 | 3 | - | 0.83 | 0.83 | 0.96 | 0.96 | 1.24 | 0.83 | 0.69 |
| 24. SMT24 | 4 | 3 | 5 | 1 | 4 | 3 | 5 | 6 | - | 0.83 | 0.41 | 0.41 | 0.69 | 0.55 | 0.41 |
| 25. SMT25 | 6 | 5 | 7 | 5 | 6 | 5 | 7 | 6 | 6 | - | 0.96 | 0.96 | 1.24 | 0.83 | 0.69 |
| 26. SMT26 | 5 | 4 | 5 | 2 | 5 | 4 | 6 | 7 | 3 | 7 | - | 0.83 | 0.83 | 0.69 | 0.55 |
| 27. SMT27 | 3 | 4 | 6 | 4 | 7 | 4 | 6 | 7 | 3 | 7 | 6 | - | 0.83 | 0.41 | 0.55 |
| 28. SMT28 | 5 | 6 | 8 | 4 | 7 | 6 | 8 | 9 | 5 | 9 | 6 | 6 | - | 0.69 | 0.83 |
| 29. SMT29 | 2 | 3 | 5 | 3 | 6 | 3 | 5 | 6 | 4 | 6 | 5 | 3 | 5 | - | 0.41 |
| 30. SMT30 | 3 | 2 | 4 | 2 | 5 | 2 | 4 | 5 | 3 | 5 | 4 | 4 | 6 | 3 | - |
| 31. SMT31 | 4 | 3 | 5 | 3 | 3 | 3 | 5 | 6 | 4 | 4 | 5 | 5 | 7 | 4 | 3 |
| 32. SMT32 | 5 | 4 | 6 | 4 | 7 | 4 | 6 | 7 | 5 | 5 | 6 | 6 | 8 | 5 | 4 |
| 33. SMT33 | 3 | 2 | 4 | 2 | 5 | 2 | 4 | 3 | 3 | 3 | 4 | 4 | 6 | 3 | 2 |
| 34. SMT34 | 5 | 4 | 6 | 4 | 6 | 4 | 6 | 7 | 5 | 7 | 6 | 6 | 8 | 5 | 4 |
| 35. SMT35 | 4 | 3 | 5 | 3 | 5 | 3 | 5 | 6 | 4 | 6 | 5 | 5 | 7 | 4 | 3 |
| 36. SMT36 | 3 | 2 | 4 | 2 | 4 | 2 | 4 | 5 | 3 | 5 | 4 | 4 | 6 | 3 | 2 |
| 37. SMT37 | 5 | 4 | 6 | 4 | 7 | 4 | 6 | 7 | 5 | 7 | 6 | 6 | 8 | 5 | 4 |
| 38. SMT38 | 3 | 2 | 4 | 2 | 5 | 2 | 4 | 5 | 3 | 5 | 4 | 4 | 6 | 3 | 2 |
| 39. SMT39 | 4 | 3 | 5 | 3 | 4 | 3 | 5 | 6 | 4 | 4 | 5 | 5 | 7 | 4 | 3 |
| 40. SMT40 | 4 | 3 | 5 | 3 | 6 | 3 | 5 | 6 | 2 | 6 | 5 | 3 | 7 | 4 | 3 |
| 41. SMT41 | 4 | 3 | 5 | 3 | 4 | 3 | 5 | 6 | 4 | 4 | 5 | 5 | 7 | 4 | 3 |
| 42. SMT42 | 5 | 4 | 6 | 4 | 7 | 4 | 6 | 7 | 5 | 7 | 6 | 6 | 8 | 5 | 4 |
| 43. SMT43 | 4 | 3 | 5 | 3 | 6 | 3 | 5 | 6 | 4 | 6 | 5 | 3 | 7 | 4 | 3 |
| 44. SMT44 | 4 | 3 | 5 | 3 | 6 | 3 | 5 | 6 | 4 | 6 | 5 | 5 | 7 | 4 | 3 |
| 45. SMT45 | 5 | 4 | 6 | 4 | 7 | 4 | 6 | 7 | 5 | 7 | 6 | 6 | 8 | 5 | 4 |
| 46. SMT46 | 5 | 4 | 6 | 4 | 7 | 4 | 4 | 5 | 5 | 7 | 6 | 6 | 8 | 5 | 4 |
| 47. SMT47 | 1 | 2 | 4 | 2 | 5 | 2 | 4 | 5 | 3 | 5 | 4 | 4 | 6 | 3 | 2 |
| 48. SMT48 | 5 | 4 | 6 | 4 | 5 | 4 | 6 | 7 | 5 | 3 | 6 | 6 | 8 | 5 | 4 |
| 49. SMT49 | 5 | 4 | 6 | 4 | 5 | 4 | 6 | 7 | 5 | 7 | 4 | 6 | 8 | 5 | 4 |
| 50. SMT50 | 4 | 3 | 5 | 3 | 6 | 3 | 5 | 6 | 4 | 6 | 5 | 5 | 7 | 4 | 3 |
| 51. SMT51 | 3 | 2 | 4 | 2 | 5 | 2 | 4 | 5 | 3 | 5 | 4 | 4 | 6 | 3 | 2 |
| 52. SMT52 | 3 | 2 | 4 | 2 | 5 | 2 | 4 | 5 | 3 | 5 | 4 | 4 | 4 | 3 | 2 |
| 53. SMT53 | 4 | 3 | 5 | 3 | 4 | 3 | 5 | 6 | 4 | 4 | 5 | 5 | 7 | 4 | 3 |
| 54. SMT54 | 2 | 2 | 4 | 2 | 5 | 2 | 4 | 5 | 3 | 5 | 4 | 4 | 6 | 3 | 2 |
| 55. SMT55 | 5 | 4 | 6 | 4 | 7 | 4 | 6 | 7 | 5 | 7 | 6 | 6 | 8 | 5 | 4 |
| 56. SMT56 | 5 | 4 | 6 | 4 | 4 | 4 | 6 | 5 | 5 | 3 | 6 | 6 | 8 | 5 | 4 |
| 57. SMT57 | 4 | 3 | 5 | 3 | 6 | 3 | 5 | 6 | 4 | 6 | 5 | 5 | 7 | 4 | 3 |
| 58. SMT58 | 4 | 3 | 5 | 3 | 6 | 3 | 5 | 6 | 4 | 6 | 5 | 5 | 7 | 4 | 3 |
| 59. SMT59 | 5 | 4 | 6 | 4 | 5 | 4 | 6 | 7 | 5 | 3 | 6 | 6 | 8 | 5 | 4 |
| 60. SMT60 | 5 | 4 | 4 | 2 | 5 | 4 | 6 | 7 | 3 | 7 | 4 | 6 | 6 | 5 | 4 |
| 61. SMT61 | 5 | 4 | 6 | 4 | 4 | 4 | 6 | 7 | 5 | 5 | 6 | 6 | 8 | 5 | 4 |
| 62. SMT62 | 5 | 4 | 6 | 4 | 7 | 2 | 6 | 7 | 5 | 7 | 6 | 6 | 8 | 5 | 4 |
| 63. SMT63 | 3 | 2 | 4 | 2 | 5 | 2 | 4 | 5 | 3 | 5 | 4 | 4 | 6 | 3 | 2 |
| 64. SMT64 | 1 | 2 | 4 | 2 | 5 | 2 | 4 | 5 | 3 | 5 | 4 | 2 | 4 | 1 | 2 |
| 65. SMT65 | 4 | 3 | 5 | 3 | 6 | 3 | 5 | 6 | 4 | 6 | 5 | 5 | 7 | 4 | 3 |
| 66. SMT66 | 3 | 2 | 4 | 2 | 5 | 2 | 4 | 5 | 3 | 5 | 4 | 4 | 6 | 3 | 2 |
| 67. SMT67 | 5 | 4 | 6 | 4 | 4 | 4 | 6 | 7 | 5 | 5 | 6 | 6 | 8 | 5 | 4 |
| 68. SMT68 | 3 | 2 | 4 | 2 | 5 | 2 | 4 | 5 | 3 | 5 | 4 | 4 | 6 | 3 | 2 |
| 69. SMT69 | 6 | 5 | 7 | 5 | 8 | 5 | 7 | 8 | 6 | 6 | 7 | 7 | 9 | 4 | 5 |
| 70. SMT70 | 5 | 4 | 6 | 4 | 7 | 4 | 6 | 7 | 5 | 7 | 6 | 6 | 8 | 5 | 4 |
| 71. SMT71 | 4 | 3 | 5 | 3 | 6 | 3 | 5 | 6 | 4 | 6 | 5 | 5 | 7 | 4 | 3 |
| 72. SMT72 | 5 | 4 | 6 | 2 | 5 | 4 | 6 | 7 | 3 | 7 | 4 | 6 | 6 | 5 | 4 |
| 73. SMT73 | 3 | 2 | 4 | 2 | 5 | 2 | 4 | 5 | 3 | 5 | 4 | 4 | 6 | 3 | 2 |

| Haplotype | 31 | 32 | 33 | 34 | 35 | 36 | 37 | 38 | 39 | 40 | 41 | 42 | 43 | 44 | 45 |
| --- | --- | --- | --- | --- | --- | --- | --- | --- | --- | --- | --- | --- | --- | --- | --- |
| 1. SMT01 | 0.41 | 0.55 | 0.28 | 0.55 | 0.41 | 0.28 | 0.55 | 0.28 | 0.41 | 0.41 | 0.41 | 0.55 | 0.41 | 0.41 | 0.55 |
| 2. SMT02 | 0.28 | 0.69 | 0.41 | 0.69 | 0.55 | 0.41 | 0.69 | 0.41 | 0.28 | 0.55 | 0.28 | 0.69 | 0.55 | 0.55 | 0.69 |
| 3. SMT03 | 0.69 | 0.83 | 0.55 | 0.83 | 0.69 | 0.55 | 0.83 | 0.55 | 0.69 | 0.69 | 0.69 | 0.83 | 0.69 | 0.69 | 0.83 |
| 4. SMT04 | 0.55 | 0.69 | 0.41 | 0.69 | 0.55 | 0.41 | 0.69 | 0.41 | 0.55 | 0.55 | 0.55 | 0.69 | 0.55 | 0.55 | 0.69 |
| 5. SMT05 | 0.55 | 0.69 | 0.41 | 0.69 | 0.55 | 0.41 | 0.69 | 0.41 | 0.55 | 0.55 | 0.55 | 0.69 | 0.55 | 0.55 | 0.69 |
| 6. SMT06 | 0.69 | 0.83 | 0.55 | 0.83 | 0.69 | 0.55 | 0.69 | 0.55 | 0.69 | 0.69 | 0.69 | 0.83 | 0.69 | 0.69 | 0.83 |
| 7. SMT07 | 0.83 | 0.96 | 0.41 | 0.96 | 0.83 | 0.69 | 0.96 | 0.69 | 0.83 | 0.83 | 0.83 | 0.96 | 0.83 | 0.83 | 0.96 |
| 8. SMT08 | 0.69 | 0.83 | 0.83 | 1.10 | 0.96 | 0.83 | 1.10 | 0.83 | 0.41 | 0.96 | 0.69 | 1.10 | 0.96 | 0.69 | 1.10 |
| 9. SMT09 | 0.69 | 1.10 | 0.83 | 1.10 | 0.96 | 0.83 | 1.10 | 0.83 | 0.69 | 0.96 | 0.69 | 0.55 | 0.96 | 0.96 | 1.10 |
| 10. SMT10 | 0.83 | 0.69 | 0.69 | 0.96 | 0.83 | 0.69 | 0.96 | 0.69 | 0.83 | 0.83 | 0.83 | 0.96 | 0.83 | 0.83 | 0.96 |
| 11. SMT11 | 0.14 | 0.83 | 0.55 | 0.55 | 0.41 | 0.28 | 0.83 | 0.55 | 0.41 | 0.69 | 0.41 | 0.83 | 0.69 | 0.69 | 0.83 |
| 12. SMT12 | 0.28 | 0.41 | 0.14 | 0.41 | 0.28 | 0.14 | 0.41 | 0.14 | 0.28 | 0.28 | 0.28 | 0.41 | 0.28 | 0.28 | 0.41 |
| 13. SMT13 | 0.41 | 0.55 | 0.28 | 0.55 | 0.41 | 0.28 | 0.55 | 0.28 | 0.41 | 0.41 | 0.41 | 0.55 | 0.41 | 0.41 | 0.55 |
| 14. SMT14 | 0.41 | 0.55 | 0.28 | 0.55 | 0.41 | 0.28 | 0.55 | 0.28 | 0.41 | 0.41 | 0.41 | 0.55 | 0.41 | 0.41 | 0.55 |
| 15. SMT15 | 0.55 | 0.69 | 0.41 | 0.69 | 0.55 | 0.41 | 0.69 | 0.41 | 0.55 | 0.55 | 0.55 | 0.69 | 0.55 | 0.55 | 0.69 |
| 16. SMT16 | 0.55 | 0.69 | 0.41 | 0.69 | 0.55 | 0.41 | 0.69 | 0.41 | 0.55 | 0.55 | 0.55 | 0.69 | 0.55 | 0.55 | 0.69 |
| 17. SMT17 | 0.41 | 0.55 | 0.28 | 0.55 | 0.41 | 0.28 | 0.55 | 0.28 | 0.41 | 0.41 | 0.41 | 0.55 | 0.41 | 0.41 | 0.55 |
| 18. SMT18 | 0.69 | 0.83 | 0.55 | 0.83 | 0.69 | 0.55 | 0.83 | 0.55 | 0.69 | 0.69 | 0.69 | 0.83 | 0.69 | 0.69 | 0.83 |
| 19. SMT19 | 0.41 | 0.55 | 0.28 | 0.55 | 0.41 | 0.28 | 0.55 | 0.28 | 0.41 | 0.41 | 0.41 | 0.55 | 0.41 | 0.41 | 0.55 |
| 20. SMT20 | 0.41 | 0.96 | 0.69 | 0.83 | 0.69 | 0.55 | 0.96 | 0.69 | 0.55 | 0.83 | 0.55 | 0.96 | 0.83 | 0.83 | 0.96 |
| 21. SMT21 | 0.41 | 0.55 | 0.28 | 0.55 | 0.41 | 0.28 | 0.55 | 0.28 | 0.41 | 0.41 | 0.41 | 0.55 | 0.41 | 0.41 | 0.55 |
| 22. SMT22 | 0.69 | 0.83 | 0.55 | 0.83 | 0.69 | 0.55 | 0.83 | 0.55 | 0.69 | 0.69 | 0.69 | 0.83 | 0.69 | 0.69 | 0.83 |
| 23. SMT23 | 0.83 | 0.96 | 0.41 | 0.96 | 0.83 | 0.69 | 0.96 | 0.69 | 0.83 | 0.83 | 0.83 | 0.96 | 0.83 | 0.83 | 0.96 |
| 24. SMT24 | 0.55 | 0.69 | 0.41 | 0.69 | 0.55 | 0.41 | 0.69 | 0.41 | 0.55 | 0.28 | 0.55 | 0.69 | 0.55 | 0.55 | 0.69 |
| 25. SMT25 | 0.55 | 0.69 | 0.41 | 0.96 | 0.83 | 0.69 | 0.96 | 0.69 | 0.55 | 0.83 | 0.55 | 0.96 | 0.83 | 0.83 | 0.96 |
| 26. SMT26 | 0.69 | 0.83 | 0.55 | 0.83 | 0.69 | 0.55 | 0.83 | 0.55 | 0.69 | 0.69 | 0.69 | 0.83 | 0.69 | 0.69 | 0.83 |
| 27. SMT27 | 0.69 | 0.83 | 0.55 | 0.83 | 0.69 | 0.55 | 0.83 | 0.55 | 0.69 | 0.41 | 0.69 | 0.83 | 0.41 | 0.69 | 0.83 |
| 28. SMT28 | 0.96 | 1.10 | 0.83 | 1.10 | 0.96 | 0.83 | 1.10 | 0.83 | 0.96 | 0.96 | 0.96 | 1.10 | 0.96 | 0.96 | 1.10 |
| 29. SMT29 | 0.55 | 0.69 | 0.41 | 0.69 | 0.55 | 0.41 | 0.69 | 0.41 | 0.55 | 0.55 | 0.55 | 0.69 | 0.55 | 0.55 | 0.69 |
| 30. SMT30 | 0.41 | 0.55 | 0.28 | 0.55 | 0.41 | 0.28 | 0.55 | 0.28 | 0.41 | 0.41 | 0.41 | 0.55 | 0.41 | 0.41 | 0.55 |
| 31. SMT31 | - | 0.69 | 0.41 | 0.41 | 0.28 | 0.14 | 0.69 | 0.41 | 0.28 | 0.55 | 0.28 | 0.69 | 0.55 | 0.55 | 0.69 |
| 32. SMT32 | 5 | - | 0.55 | 0.83 | 0.69 | 0.55 | 0.83 | 0.55 | 0.69 | 0.69 | 0.69 | 0.83 | 0.69 | 0.69 | 0.83 |
| 33. SMT33 | 3 | 4 | - | 0.55 | 0.41 | 0.28 | 0.55 | 0.28 | 0.41 | 0.41 | 0.41 | 0.55 | 0.41 | 0.41 | 0.55 |
| 34. SMT34 | 3 | 6 | 4 | - | 0.14 | 0.28 | 0.83 | 0.55 | 0.69 | 0.69 | 0.69 | 0.83 | 0.69 | 0.69 | 0.83 |
| 35. SMT35 | 2 | 5 | 3 | 1 | - | 0.14 | 0.69 | 0.41 | 0.55 | 0.55 | 0.55 | 0.69 | 0.55 | 0.55 | 0.69 |
| 36. SMT36 | 1 | 4 | 2 | 2 | 1 | - | 0.55 | 0.28 | 0.41 | 0.41 | 0.41 | 0.55 | 0.41 | 0.41 | 0.55 |
| 37. SMT37 | 5 | 6 | 4 | 6 | 5 | 4 | - | 0.55 | 0.69 | 0.69 | 0.69 | 0.83 | 0.69 | 0.69 | 0.83 |
| 38. SMT38 | 3 | 4 | 2 | 4 | 3 | 2 | 4 | - | 0.41 | 0.41 | 0.41 | 0.55 | 0.41 | 0.41 | 0.55 |
| 39. SMT39 | 2 | 5 | 3 | 5 | 4 | 3 | 5 | 3 | - | 0.55 | 0.28 | 0.69 | 0.55 | 0.28 | 0.69 |
| 40. SMT40 | 4 | 5 | 3 | 5 | 4 | 3 | 5 | 3 | 4 | - | 0.55 | 0.69 | 0.55 | 0.55 | 0.69 |
| 41. SMT41 | 2 | 5 | 3 | 5 | 4 | 3 | 5 | 3 | 2 | 4 | - | 0.69 | 0.55 | 0.55 | 0.69 |
| 42. SMT42 | 5 | 6 | 4 | 6 | 5 | 4 | 6 | 4 | 5 | 5 | 5 | - | 0.69 | 0.69 | 0.83 |
| 43. SMT43 | 4 | 5 | 3 | 5 | 4 | 3 | 5 | 3 | 4 | 4 | 4 | 5 | - | 0.55 | 0.69 |
| 44. SMT44 | 4 | 5 | 3 | 5 | 4 | 3 | 5 | 3 | 2 | 4 | 4 | 5 | 4 | - | 0.69 |
| 45. SMT45 | 5 | 6 | 4 | 6 | 5 | 4 | 6 | 4 | 5 | 5 | 5 | 6 | 5 | 5 | - |
| 46. SMT46 | 5 | 6 | 4 | 6 | 5 | 4 | 6 | 4 | 5 | 5 | 5 | 6 | 5 | 5 | 6 |
| 47. SMT47 | 3 | 4 | 2 | 4 | 3 | 2 | 4 | 2 | 3 | 3 | 3 | 4 | 3 | 3 | 4 |
| 48. SMT48 | 3 | 4 | 4 | 6 | 5 | 4 | 6 | 4 | 3 | 5 | 3 | 6 | 5 | 5 | 6 |
| 49. SMT49 | 5 | 6 | 4 | 6 | 5 | 4 | 6 | 4 | 5 | 5 | 5 | 6 | 5 | 5 | 6 |
| 50. SMT50 | 4 | 5 | 3 | 5 | 4 | 3 | 5 | 3 | 4 | 4 | 4 | 5 | 4 | 4 | 5 |
| 51. SMT51 | 3 | 4 | 2 | 4 | 3 | 2 | 4 | 2 | 3 | 3 | 3 | 4 | 3 | 3 | 4 |
| 52. SMT52 | 3 | 4 | 2 | 4 | 3 | 2 | 4 | 2 | 3 | 3 | 3 | 4 | 3 | 3 | 4 |
| 53. SMT53 | 2 | 5 | 3 | 5 | 4 | 3 | 5 | 3 | 2 | 4 | 2 | 5 | 4 | 4 | 5 |
| 54. SMT54 | 3 | 4 | 2 | 4 | 3 | 2 | 4 | 2 | 3 | 3 | 3 | 4 | 3 | 3 | 4 |
| 55. SMT55 | 5 | 4 | 4 | 6 | 5 | 4 | 6 | 2 | 5 | 5 | 5 | 6 | 5 | 5 | 6 |
| 56. SMT56 | 1 | 6 | 2 | 4 | 3 | 2 | 6 | 4 | 3 | 5 | 3 | 6 | 5 | 5 | 6 |
| 57. SMT57 | 4 | 5 | 3 | 5 | 4 | 3 | 5 | 3 | 4 | 4 | 4 | 5 | 4 | 4 | 5 |
| 58. SMT58 | 4 | 5 | 3 | 5 | 4 | 3 | 3 | 3 | 4 | 4 | 4 | 5 | 4 | 4 | 3 |
| 59. SMT59 | 3 | 6 | 4 | 6 | 5 | 4 | 6 | 4 | 3 | 5 | 3 | 6 | 5 | 5 | 6 |
| 60. SMT60 | 5 | 6 | 4 | 6 | 5 | 4 | 6 | 4 | 5 | 5 | 5 | 6 | 5 | 5 | 6 |
| 61. SMT61 | 1 | 6 | 4 | 4 | 3 | 2 | 6 | 4 | 3 | 5 | 3 | 6 | 5 | 5 | 6 |
| 62. SMT62 | 5 | 6 | 4 | 6 | 5 | 4 | 6 | 4 | 5 | 5 | 5 | 6 | 5 | 5 | 6 |
| 63. SMT63 | 3 | 4 | 2 | 4 | 3 | 2 | 4 | 2 | 3 | 3 | 3 | 4 | 3 | 1 | 4 |
| 64. SMT64 | 3 | 4 | 2 | 4 | 3 | 2 | 4 | 2 | 3 | 3 | 3 | 4 | 3 | 3 | 4 |
| 65. SMT65 | 4 | 5 | 3 | 5 | 4 | 3 | 5 | 3 | 4 | 4 | 4 | 5 | 4 | 4 | 5 |
| 66. SMT66 | 3 | 4 | 2 | 4 | 3 | 2 | 4 | 2 | 3 | 3 | 3 | 4 | 3 | 3 | 4 |
| 67. SMT67 | 1 | 6 | 4 | 4 | 3 | 2 | 6 | 4 | 3 | 5 | 3 | 6 | 5 | 5 | 6 |
| 68. SMT68 | 3 | 4 | 2 | 4 | 3 | 2 | 4 | 2 | 3 | 3 | 3 | 4 | 3 | 3 | 4 |
| 69. SMT69 | 6 | 7 | 5 | 6 | 5 | 5 | 7 | 3 | 6 | 6 | 6 | 7 | 6 | 6 | 7 |
| 70. SMT70 | 5 | 6 | 4 | 6 | 5 | 4 | 6 | 4 | 5 | 5 | 5 | 4 | 5 | 5 | 6 |
| 71. SMT71 | 4 | 5 | 3 | 5 | 4 | 3 | 5 | 3 | 4 | 4 | 4 | 5 | 2 | 4 | 5 |
| 72. SMT72 | 5 | 6 | 4 | 6 | 5 | 4 | 6 | 4 | 5 | 5 | 5 | 6 | 3 | 5 | 6 |
| 73. SMT73 | 3 | 4 | 2 | 4 | 3 | 2 | 4 | 2 | 3 | 3 | 3 | 4 | 3 | 3 | 4 |

| Haplotype | 46 | 47 | 48 | 49 | 50 | 51 | 52 | 53 | 54 | 55 | 56 | 57 | 58 | 59 |
| --- | --- | --- | --- | --- | --- | --- | --- | --- | --- | --- | --- | --- | --- | --- |
| 1. SMT01 | 0.55 | 0.28 | 0.55 | 0.55 | 0.41 | 0.28 | 0.28 | 0.41 | 0.28 | 0.55 | 0.55 | 0.41 | 0.41 | 0.55 |
| 2. SMT02 | 0.69 | 0.41 | 0.41 | 0.69 | 0.55 | 0.41 | 0.41 | 0.28 | 0.41 | 0.69 | 0.41 | 0.55 | 0.55 | 0.41 |
| 3. SMT03 | 0.83 | 0.55 | 0.83 | 0.83 | 0.69 | 0.55 | 0.55 | 0.69 | 0.55 | 0.83 | 0.83 | 0.69 | 0.69 | 0.83 |
| 4. SMT04 | 0.69 | 0.41 | 0.69 | 0.69 | 0.55 | 0.41 | 0.41 | 0.55 | 0.41 | 0.69 | 0.69 | 0.55 | 0.55 | 0.69 |
| 5. SMT05 | 0.41 | 0.41 | 0.69 | 0.69 | 0.55 | 0.41 | 0.41 | 0.55 | 0.41 | 0.69 | 0.69 | 0.55 | 0.55 | 0.69 |
| 6. SMT06 | 0.83 | 0.55 | 0.83 | 0.83 | 0.69 | 0.55 | 0.55 | 0.41 | 0.55 | 0.83 | 0.83 | 0.69 | 0.69 | 0.83 |
| 7. SMT07 | 0.69 | 0.69 | 0.96 | 0.96 | 0.83 | 0.69 | 0.69 | 0.83 | 0.69 | 0.96 | 0.69 | 0.83 | 0.83 | 0.96 |
| 8. SMT08 | 1.10 | 0.83 | 0.83 | 1.10 | 0.96 | 0.83 | 0.83 | 0.69 | 0.83 | 0.83 | 0.83 | 0.96 | 0.96 | 0.83 |
| 9. SMT09 | 1.10 | 0.83 | 0.83 | 1.10 | 0.96 | 0.83 | 0.83 | 0.69 | 0.83 | 1.10 | 0.83 | 0.96 | 0.96 | 0.83 |
| 10. SMT10 | 0.96 | 0.69 | 0.69 | 0.96 | 0.83 | 0.69 | 0.69 | 0.83 | 0.69 | 0.96 | 0.96 | 0.83 | 0.83 | 0.96 |
| 11. SMT11 | 0.83 | 0.55 | 0.55 | 0.83 | 0.69 | 0.55 | 0.55 | 0.41 | 0.55 | 0.83 | 0.28 | 0.69 | 0.69 | 0.55 |
| 12. SMT12 | 0.41 | 0.14 | 0.41 | 0.41 | 0.28 | 0.14 | 0.14 | 0.28 | 0.14 | 0.41 | 0.41 | 0.28 | 0.28 | 0.41 |
| 13. SMT13 | 0.55 | 0.28 | 0.55 | 0.55 | 0.41 | 0.28 | 0.28 | 0.41 | 0.28 | 0.55 | 0.55 | 0.41 | 0.41 | 0.55 |
| 14. SMT14 | 0.55 | 0.28 | 0.55 | 0.55 | 0.41 | 0.28 | 0.28 | 0.41 | 0.28 | 0.55 | 0.55 | 0.41 | 0.41 | 0.55 |
| 15. SMT15 | 0.69 | 0.41 | 0.69 | 0.69 | 0.55 | 0.41 | 0.41 | 0.55 | 0.41 | 0.69 | 0.69 | 0.55 | 0.55 | 0.69 |
| 16. SMT16 | 0.69 | 0.14 | 0.69 | 0.69 | 0.55 | 0.41 | 0.41 | 0.55 | 0.28 | 0.69 | 0.69 | 0.55 | 0.55 | 0.69 |
| 17. SMT17 | 0.55 | 0.28 | 0.55 | 0.55 | 0.41 | 0.28 | 0.28 | 0.41 | 0.28 | 0.55 | 0.55 | 0.41 | 0.41 | 0.55 |
| 18. SMT18 | 0.83 | 0.55 | 0.83 | 0.83 | 0.69 | 0.55 | 0.55 | 0.69 | 0.55 | 0.83 | 0.83 | 0.69 | 0.69 | 0.83 |
| 19. SMT19 | 0.55 | 0.28 | 0.55 | 0.55 | 0.41 | 0.28 | 0.28 | 0.41 | 0.28 | 0.55 | 0.55 | 0.41 | 0.41 | 0.55 |
| 20. SMT20 | 0.96 | 0.69 | 0.69 | 0.69 | 0.83 | 0.69 | 0.69 | 0.55 | 0.69 | 0.96 | 0.55 | 0.83 | 0.83 | 0.69 |
| 21. SMT21 | 0.55 | 0.28 | 0.55 | 0.55 | 0.41 | 0.28 | 0.28 | 0.41 | 0.28 | 0.55 | 0.55 | 0.41 | 0.41 | 0.55 |
| 22. SMT22 | 0.55 | 0.55 | 0.83 | 0.83 | 0.69 | 0.55 | 0.55 | 0.69 | 0.55 | 0.83 | 0.83 | 0.69 | 0.69 | 0.83 |
| 23. SMT23 | 0.69 | 0.69 | 0.96 | 0.96 | 0.83 | 0.69 | 0.69 | 0.83 | 0.69 | 0.96 | 0.69 | 0.83 | 0.83 | 0.96 |
| 24. SMT24 | 0.69 | 0.41 | 0.69 | 0.69 | 0.55 | 0.41 | 0.41 | 0.55 | 0.41 | 0.69 | 0.69 | 0.55 | 0.55 | 0.69 |
| 25. SMT25 | 0.96 | 0.69 | 0.41 | 0.96 | 0.83 | 0.69 | 0.69 | 0.55 | 0.69 | 0.96 | 0.41 | 0.83 | 0.83 | 0.41 |
| 26. SMT26 | 0.83 | 0.55 | 0.83 | 0.55 | 0.69 | 0.55 | 0.55 | 0.69 | 0.55 | 0.83 | 0.83 | 0.69 | 0.69 | 0.83 |
| 27. SMT27 | 0.83 | 0.55 | 0.83 | 0.83 | 0.69 | 0.55 | 0.55 | 0.69 | 0.55 | 0.83 | 0.83 | 0.69 | 0.69 | 0.83 |
| 28. SMT28 | 1.10 | 0.83 | 1.10 | 1.10 | 0.96 | 0.83 | 0.55 | 0.96 | 0.83 | 1.10 | 1.10 | 0.96 | 0.96 | 1.10 |
| 29. SMT29 | 0.69 | 0.41 | 0.69 | 0.69 | 0.55 | 0.41 | 0.41 | 0.55 | 0.41 | 0.69 | 0.69 | 0.55 | 0.55 | 0.69 |
| 30. SMT30 | 0.55 | 0.28 | 0.55 | 0.55 | 0.41 | 0.28 | 0.28 | 0.41 | 0.28 | 0.55 | 0.55 | 0.41 | 0.41 | 0.55 |
| 31. SMT31 | 0.69 | 0.41 | 0.41 | 0.69 | 0.55 | 0.41 | 0.41 | 0.28 | 0.41 | 0.69 | 0.14 | 0.55 | 0.55 | 0.41 |
| 32. SMT32 | 0.83 | 0.55 | 0.55 | 0.83 | 0.69 | 0.55 | 0.55 | 0.69 | 0.55 | 0.55 | 0.83 | 0.69 | 0.69 | 0.83 |
| 33. SMT33 | 0.55 | 0.28 | 0.55 | 0.55 | 0.41 | 0.28 | 0.28 | 0.41 | 0.28 | 0.55 | 0.28 | 0.41 | 0.41 | 0.55 |
| 34. SMT34 | 0.83 | 0.55 | 0.83 | 0.83 | 0.69 | 0.55 | 0.55 | 0.69 | 0.55 | 0.83 | 0.55 | 0.69 | 0.69 | 0.83 |
| 35. SMT35 | 0.69 | 0.41 | 0.69 | 0.69 | 0.55 | 0.41 | 0.41 | 0.55 | 0.41 | 0.69 | 0.41 | 0.55 | 0.55 | 0.69 |
| 36. SMT36 | 0.55 | 0.28 | 0.55 | 0.55 | 0.41 | 0.28 | 0.28 | 0.41 | 0.28 | 0.55 | 0.28 | 0.41 | 0.41 | 0.55 |
| 37. SMT37 | 0.83 | 0.55 | 0.83 | 0.83 | 0.69 | 0.55 | 0.55 | 0.69 | 0.55 | 0.83 | 0.83 | 0.69 | 0.41 | 0.83 |
| 38. SMT38 | 0.55 | 0.28 | 0.55 | 0.55 | 0.41 | 0.28 | 0.28 | 0.41 | 0.28 | 0.28 | 0.55 | 0.41 | 0.41 | 0.55 |
| 39. SMT39 | 0.69 | 0.41 | 0.41 | 0.69 | 0.55 | 0.41 | 0.41 | 0.28 | 0.41 | 0.69 | 0.41 | 0.55 | 0.55 | 0.41 |
| 40. SMT40 | 0.69 | 0.41 | 0.69 | 0.69 | 0.55 | 0.41 | 0.41 | 0.55 | 0.41 | 0.69 | 0.69 | 0.55 | 0.55 | 0.69 |
| 41. SMT41 | 0.69 | 0.41 | 0.41 | 0.69 | 0.55 | 0.41 | 0.41 | 0.28 | 0.41 | 0.69 | 0.41 | 0.55 | 0.55 | 0.41 |
| 42. SMT42 | 0.83 | 0.55 | 0.83 | 0.83 | 0.69 | 0.55 | 0.55 | 0.69 | 0.55 | 0.83 | 0.83 | 0.69 | 0.69 | 0.83 |
| 43. SMT43 | 0.69 | 0.41 | 0.69 | 0.69 | 0.55 | 0.41 | 0.41 | 0.55 | 0.41 | 0.69 | 0.69 | 0.55 | 0.55 | 0.69 |
| 44. SMT44 | 0.69 | 0.41 | 0.69 | 0.69 | 0.55 | 0.41 | 0.41 | 0.55 | 0.41 | 0.69 | 0.69 | 0.55 | 0.55 | 0.69 |
| 45. SMT45 | 0.83 | 0.55 | 0.83 | 0.83 | 0.69 | 0.55 | 0.55 | 0.69 | 0.55 | 0.83 | 0.83 | 0.69 | 0.41 | 0.83 |
| 46. SMT46 | - | 0.55 | 0.83 | 0.83 | 0.69 | 0.55 | 0.55 | 0.69 | 0.55 | 0.83 | 0.83 | 0.69 | 0.69 | 0.83 |
| 47. SMT47 | 4 | - | 0.55 | 0.55 | 0.41 | 0.28 | 0.28 | 0.41 | 0.14 | 0.55 | 0.55 | 0.41 | 0.41 | 0.55 |
| 48. SMT48 | 6 | 4 | - | 0.83 | 0.69 | 0.55 | 0.55 | 0.41 | 0.55 | 0.83 | 0.55 | 0.69 | 0.69 | 0.55 |
| 49. SMT49 | 6 | 4 | 6 | - | 0.69 | 0.55 | 0.55 | 0.69 | 0.55 | 0.83 | 0.83 | 0.69 | 0.69 | 0.83 |
| 50. SMT50 | 5 | 3 | 5 | 5 | - | 0.41 | 0.41 | 0.55 | 0.41 | 0.69 | 0.69 | 0.55 | 0.55 | 0.69 |
| 51. SMT51 | 4 | 2 | 4 | 4 | 3 | - | 0.28 | 0.41 | 0.28 | 0.55 | 0.55 | 0.41 | 0.41 | 0.55 |
| 52. SMT52 | 4 | 2 | 4 | 4 | 3 | 2 | - | 0.41 | 0.28 | 0.55 | 0.55 | 0.41 | 0.41 | 0.55 |
| 53. SMT53 | 5 | 3 | 3 | 5 | 4 | 3 | 3 | - | 0.41 | 0.69 | 0.41 | 0.55 | 0.55 | 0.41 |
| 54. SMT54 | 4 | 1 | 4 | 4 | 3 | 2 | 2 | 3 | - | 0.55 | 0.55 | 0.41 | 0.41 | 0.55 |
| 55. SMT55 | 6 | 4 | 6 | 6 | 5 | 4 | 4 | 5 | 4 | - | 0.83 | 0.69 | 0.69 | 0.83 |
| 56. SMT56 | 6 | 4 | 4 | 6 | 5 | 4 | 4 | 3 | 4 | 6 | - | 0.69 | 0.69 | 0.55 |
| 57. SMT57 | 5 | 3 | 5 | 5 | 4 | 3 | 3 | 4 | 3 | 5 | 5 | - | 0.55 | 0.69 |
| 58. SMT58 | 5 | 3 | 5 | 5 | 4 | 3 | 3 | 4 | 3 | 5 | 5 | 4 | - | 0.69 |
| 59. SMT59 | 6 | 4 | 4 | 6 | 5 | 4 | 4 | 3 | 4 | 6 | 4 | 5 | 5 | - |
| 60. SMT60 | 6 | 4 | 6 | 6 | 5 | 4 | 4 | 5 | 4 | 6 | 6 | 5 | 5 | 6 |
| 61. SMT61 | 6 | 4 | 4 | 6 | 5 | 4 | 4 | 3 | 4 | 6 | 2 | 5 | 5 | 4 |
| 62. SMT62 | 6 | 4 | 6 | 6 | 5 | 4 | 4 | 5 | 4 | 6 | 6 | 5 | 5 | 6 |
| 63. SMT63 | 4 | 2 | 4 | 4 | 3 | 2 | 2 | 3 | 2 | 4 | 4 | 3 | 3 | 4 |
| 64. SMT64 | 4 | 2 | 4 | 4 | 3 | 2 | 2 | 3 | 2 | 4 | 4 | 3 | 3 | 4 |
| 65. SMT65 | 5 | 3 | 5 | 5 | 4 | 3 | 3 | 4 | 3 | 5 | 5 | 4 | 4 | 5 |
| 66. SMT66 | 4 | 2 | 4 | 4 | 3 | 2 | 2 | 3 | 2 | 4 | 4 | 3 | 3 | 4 |
| 67. SMT67 | 6 | 4 | 4 | 6 | 5 | 4 | 4 | 3 | 4 | 6 | 2 | 3 | 5 | 4 |
| 68. SMT68 | 4 | 2 | 4 | 4 | 1 | 2 | 2 | 3 | 2 | 4 | 4 | 3 | 3 | 4 |
| 69. SMT69 | 7 | 5 | 7 | 7 | 6 | 5 | 5 | 6 | 5 | 5 | 7 | 6 | 6 | 5 |
| 70. SMT70 | 6 | 4 | 6 | 6 | 5 | 4 | 4 | 5 | 4 | 6 | 6 | 5 | 5 | 6 |
| 71. SMT71 | 5 | 3 | 5 | 5 | 4 | 3 | 3 | 4 | 3 | 5 | 5 | 4 | 4 | 5 |
| 72. SMT72 | 6 | 4 | 6 | 6 | 5 | 4 | 4 | 5 | 4 | 6 | 6 | 5 | 5 | 6 |
| 73. SMT73 | 4 | 2 | 4 | 4 | 3 | 2 | 2 | 3 | 2 | 4 | 4 | 3 | 3 | 4 |

| Haplotype | 60 | 61 | 62 | 63 | 64 | 65 | 66 | 67 | 68 | 69 | 70 | 71 | 72 | 73 |
| --- | --- | --- | --- | --- | --- | --- | --- | --- | --- | --- | --- | --- | --- | --- |
| 1. SMT01 | 0.55 | 0.55 | 0.55 | 0.28 | 0.28 | 0.41 | 0.28 | 0.55 | 0.28 | 0.69 | 0.55 | 0.41 | 0.55 | 0.28 |
| 2. SMT02 | 0.41 | 0.41 | 0.69 | 0.41 | 0.41 | 0.55 | 0.41 | 0.41 | 0.41 | 0.83 | 0.69 | 0.55 | 0.41 | 0.41 |
| 3. SMT03 | 0.28 | 0.83 | 0.83 | 0.55 | 0.55 | 0.69 | 0.55 | 0.83 | 0.55 | 0.96 | 0.83 | 0.69 | 0.55 | 0.55 |
| 4. SMT04 | 0.69 | 0.69 | 0.69 | 0.41 | 0.41 | 0.55 | 0.41 | 0.69 | 0.41 | 0.83 | 0.69 | 0.55 | 0.69 | 0.41 |
| 5. SMT05 | 0.41 | 0.69 | 0.69 | 0.41 | 0.41 | 0.55 | 0.41 | 0.69 | 0.41 | 0.83 | 0.69 | 0.55 | 0.41 | 0.41 |
| 6. SMT06 | 0.55 | 0.83 | 0.83 | 0.55 | 0.55 | 0.69 | 0.55 | 0.83 | 0.55 | 0.96 | 0.83 | 0.69 | 0.55 | 0.55 |
| 7. SMT07 | 0.96 | 0.96 | 0.96 | 0.69 | 0.41 | 0.83 | 0.69 | 0.96 | 0.69 | 1.10 | 0.96 | 0.83 | 0.96 | 0.69 |
| 8. SMT08 | 1.10 | 0.83 | 1.10 | 0.83 | 0.83 | 0.96 | 0.83 | 0.83 | 0.83 | 1.24 | 1.10 | 0.96 | 1.10 | 0.83 |
| 9. SMT09 | 1.10 | 0.83 | 1.10 | 0.83 | 0.83 | 0.96 | 0.83 | 0.83 | 0.83 | 1.24 | 1.10 | 0.96 | 1.10 | 0.83 |
| 10. SMT10 | 0.69 | 0.96 | 0.96 | 0.69 | 0.69 | 0.83 | 0.69 | 0.96 | 0.69 | 1.10 | 0.96 | 0.83 | 0.69 | 0.69 |
| 11. SMT11 | 0.83 | 0.28 | 0.83 | 0.55 | 0.55 | 0.69 | 0.55 | 0.28 | 0.55 | 0.96 | 0.83 | 0.69 | 0.83 | 0.55 |
| 12. SMT12 | 0.41 | 0.41 | 0.41 | 0.14 | 0.14 | 0.28 | 0.14 | 0.41 | 0.14 | 0.55 | 0.41 | 0.28 | 0.41 | 0.14 |
| 13. SMT13 | 0.55 | 0.55 | 0.55 | 0.28 | 0.28 | 0.41 | 0.28 | 0.55 | 0.28 | 0.69 | 0.55 | 0.41 | 0.55 | 0.28 |
| 14. SMT14 | 0.55 | 0.55 | 0.55 | 0.28 | 0.28 | 0.41 | 0.28 | 0.55 | 0.28 | 0.69 | 0.55 | 0.41 | 0.55 | 0.28 |
| 15. SMT15 | 0.69 | 0.69 | 0.69 | 0.41 | 0.41 | 0.55 | 0.41 | 0.69 | 0.41 | 0.55 | 0.69 | 0.55 | 0.69 | 0.41 |
| 16. SMT16 | 0.69 | 0.69 | 0.69 | 0.41 | 0.14 | 0.55 | 0.41 | 0.69 | 0.41 | 0.83 | 0.69 | 0.55 | 0.69 | 0.41 |
| 17. SMT17 | 0.55 | 0.55 | 0.55 | 0.28 | 0.28 | 0.41 | 0.28 | 0.55 | 0.28 | 0.69 | 0.55 | 0.41 | 0.55 | 0.28 |
| 18. SMT18 | 0.55 | 0.83 | 0.83 | 0.55 | 0.55 | 0.69 | 0.55 | 0.83 | 0.55 | 0.96 | 0.83 | 0.69 | 0.83 | 0.55 |
| 19. SMT19 | 0.28 | 0.55 | 0.55 | 0.28 | 0.28 | 0.41 | 0.28 | 0.55 | 0.28 | 0.69 | 0.55 | 0.41 | 0.28 | 0.28 |
| 20. SMT20 | 0.69 | 0.55 | 0.96 | 0.69 | 0.69 | 0.83 | 0.69 | 0.55 | 0.69 | 1.10 | 0.96 | 0.83 | 0.69 | 0.69 |
| 21. SMT21 | 0.55 | 0.55 | 0.28 | 0.28 | 0.28 | 0.41 | 0.28 | 0.55 | 0.28 | 0.69 | 0.55 | 0.41 | 0.55 | 0.28 |
| 22. SMT22 | 0.83 | 0.83 | 0.83 | 0.55 | 0.55 | 0.69 | 0.55 | 0.83 | 0.55 | 0.96 | 0.83 | 0.69 | 0.83 | 0.55 |
| 23. SMT23 | 0.96 | 0.96 | 0.96 | 0.69 | 0.69 | 0.83 | 0.69 | 0.96 | 0.69 | 1.10 | 0.96 | 0.83 | 0.96 | 0.69 |
| 24. SMT24 | 0.41 | 0.69 | 0.69 | 0.41 | 0.41 | 0.55 | 0.41 | 0.69 | 0.41 | 0.83 | 0.69 | 0.55 | 0.41 | 0.41 |
| 25. SMT25 | 0.96 | 0.69 | 0.96 | 0.69 | 0.69 | 0.83 | 0.69 | 0.69 | 0.69 | 0.83 | 0.96 | 0.83 | 0.96 | 0.69 |
| 26. SMT26 | 0.55 | 0.83 | 0.83 | 0.55 | 0.55 | 0.69 | 0.55 | 0.83 | 0.55 | 0.96 | 0.83 | 0.69 | 0.55 | 0.55 |
| 27. SMT27 | 0.83 | 0.83 | 0.83 | 0.55 | 0.28 | 0.69 | 0.55 | 0.83 | 0.55 | 0.96 | 0.83 | 0.69 | 0.83 | 0.55 |
| 28. SMT28 | 0.83 | 1.10 | 1.10 | 0.83 | 0.55 | 0.96 | 0.83 | 1.10 | 0.83 | 1.24 | 1.10 | 0.96 | 0.83 | 0.83 |
| 29. SMT29 | 0.69 | 0.69 | 0.69 | 0.41 | 0.14 | 0.55 | 0.41 | 0.69 | 0.41 | 0.55 | 0.69 | 0.55 | 0.69 | 0.41 |
| 30. SMT30 | 0.55 | 0.55 | 0.55 | 0.28 | 0.28 | 0.41 | 0.28 | 0.55 | 0.28 | 0.69 | 0.55 | 0.41 | 0.55 | 0.28 |
| 31. SMT31 | 0.69 | 0.14 | 0.69 | 0.41 | 0.41 | 0.55 | 0.41 | 0.14 | 0.41 | 0.83 | 0.69 | 0.55 | 0.69 | 0.41 |
| 32. SMT32 | 0.83 | 0.83 | 0.83 | 0.55 | 0.55 | 0.69 | 0.55 | 0.83 | 0.55 | 0.96 | 0.83 | 0.69 | 0.83 | 0.55 |
| 33. SMT33 | 0.55 | 0.55 | 0.55 | 0.28 | 0.28 | 0.41 | 0.28 | 0.55 | 0.28 | 0.69 | 0.55 | 0.41 | 0.55 | 0.28 |
| 34. SMT34 | 0.83 | 0.55 | 0.83 | 0.55 | 0.55 | 0.69 | 0.55 | 0.55 | 0.55 | 0.83 | 0.83 | 0.69 | 0.83 | 0.55 |
| 35. SMT35 | 0.69 | 0.41 | 0.69 | 0.41 | 0.41 | 0.55 | 0.41 | 0.41 | 0.41 | 0.69 | 0.69 | 0.55 | 0.69 | 0.41 |
| 36. SMT36 | 0.55 | 0.28 | 0.55 | 0.28 | 0.28 | 0.41 | 0.28 | 0.28 | 0.28 | 0.69 | 0.55 | 0.41 | 0.55 | 0.28 |
| 37. SMT37 | 0.83 | 0.83 | 0.83 | 0.55 | 0.55 | 0.69 | 0.55 | 0.83 | 0.55 | 0.96 | 0.83 | 0.69 | 0.83 | 0.55 |
| 38. SMT38 | 0.55 | 0.55 | 0.55 | 0.28 | 0.28 | 0.41 | 0.28 | 0.55 | 0.28 | 0.41 | 0.55 | 0.41 | 0.55 | 0.28 |
| 39. SMT39 | 0.69 | 0.41 | 0.69 | 0.41 | 0.41 | 0.55 | 0.41 | 0.41 | 0.41 | 0.83 | 0.69 | 0.55 | 0.69 | 0.41 |
| 40. SMT40 | 0.69 | 0.69 | 0.69 | 0.41 | 0.41 | 0.55 | 0.41 | 0.69 | 0.41 | 0.83 | 0.69 | 0.55 | 0.69 | 0.41 |
| 41. SMT41 | 0.69 | 0.41 | 0.69 | 0.41 | 0.41 | 0.55 | 0.41 | 0.41 | 0.41 | 0.83 | 0.69 | 0.55 | 0.69 | 0.41 |
| 42. SMT42 | 0.83 | 0.83 | 0.83 | 0.55 | 0.55 | 0.69 | 0.55 | 0.83 | 0.55 | 0.96 | 0.55 | 0.69 | 0.83 | 0.55 |
| 43. SMT43 | 0.69 | 0.69 | 0.69 | 0.41 | 0.41 | 0.55 | 0.41 | 0.69 | 0.41 | 0.83 | 0.69 | 0.28 | 0.41 | 0.41 |
| 44. SMT44 | 0.69 | 0.69 | 0.69 | 0.14 | 0.41 | 0.55 | 0.41 | 0.69 | 0.41 | 0.83 | 0.69 | 0.55 | 0.69 | 0.41 |
| 45. SMT45 | 0.83 | 0.83 | 0.83 | 0.55 | 0.55 | 0.69 | 0.55 | 0.83 | 0.55 | 0.96 | 0.83 | 0.69 | 0.83 | 0.55 |
| 46. SMT46 | 0.83 | 0.83 | 0.83 | 0.55 | 0.55 | 0.69 | 0.55 | 0.83 | 0.55 | 0.96 | 0.83 | 0.69 | 0.83 | 0.55 |
| 47. SMT47 | 0.55 | 0.55 | 0.55 | 0.28 | 0.28 | 0.41 | 0.28 | 0.55 | 0.28 | 0.69 | 0.55 | 0.41 | 0.55 | 0.28 |
| 48. SMT48 | 0.83 | 0.55 | 0.83 | 0.55 | 0.55 | 0.69 | 0.55 | 0.55 | 0.55 | 0.96 | 0.83 | 0.69 | 0.83 | 0.55 |
| 49. SMT49 | 0.83 | 0.83 | 0.83 | 0.55 | 0.55 | 0.69 | 0.55 | 0.83 | 0.55 | 0.96 | 0.83 | 0.69 | 0.83 | 0.55 |
| 50. SMT50 | 0.69 | 0.69 | 0.69 | 0.41 | 0.41 | 0.55 | 0.41 | 0.69 | 0.14 | 0.83 | 0.69 | 0.55 | 0.69 | 0.41 |
| 51. SMT51 | 0.55 | 0.55 | 0.55 | 0.28 | 0.28 | 0.41 | 0.28 | 0.55 | 0.28 | 0.69 | 0.55 | 0.41 | 0.55 | 0.28 |
| 52. SMT52 | 0.55 | 0.55 | 0.55 | 0.28 | 0.28 | 0.41 | 0.28 | 0.55 | 0.28 | 0.69 | 0.55 | 0.41 | 0.55 | 0.28 |
| 53. SMT53 | 0.69 | 0.41 | 0.69 | 0.41 | 0.41 | 0.55 | 0.41 | 0.41 | 0.41 | 0.83 | 0.69 | 0.55 | 0.69 | 0.41 |
| 54. SMT54 | 0.55 | 0.55 | 0.55 | 0.28 | 0.28 | 0.41 | 0.28 | 0.55 | 0.28 | 0.69 | 0.55 | 0.41 | 0.55 | 0.28 |
| 55. SMT55 | 0.83 | 0.83 | 0.83 | 0.55 | 0.55 | 0.69 | 0.55 | 0.83 | 0.55 | 0.69 | 0.83 | 0.69 | 0.83 | 0.55 |
| 56. SMT56 | 0.83 | 0.28 | 0.83 | 0.55 | 0.55 | 0.69 | 0.55 | 0.28 | 0.55 | 0.96 | 0.83 | 0.69 | 0.83 | 0.55 |
| 57. SMT57 | 0.69 | 0.69 | 0.69 | 0.41 | 0.41 | 0.55 | 0.41 | 0.41 | 0.41 | 0.83 | 0.69 | 0.55 | 0.69 | 0.41 |
| 58. SMT58 | 0.69 | 0.69 | 0.69 | 0.41 | 0.41 | 0.55 | 0.41 | 0.69 | 0.41 | 0.83 | 0.69 | 0.55 | 0.69 | 0.41 |
| 59. SMT59 | 0.83 | 0.55 | 0.83 | 0.55 | 0.55 | 0.69 | 0.55 | 0.55 | 0.55 | 0.69 | 0.83 | 0.69 | 0.83 | 0.55 |
| 60. SMT60 | - | 0.83 | 0.83 | 0.55 | 0.55 | 0.69 | 0.55 | 0.83 | 0.55 | 0.96 | 0.83 | 0.69 | 0.55 | 0.55 |
| 61. SMT61 | 6 | - | 0.83 | 0.55 | 0.55 | 0.69 | 0.55 | 0.28 | 0.55 | 0.96 | 0.83 | 0.69 | 0.83 | 0.55 |
| 62. SMT62 | 6 | 6 | - | 0.55 | 0.55 | 0.69 | 0.55 | 0.83 | 0.55 | 0.96 | 0.83 | 0.69 | 0.83 | 0.55 |
| 63. SMT63 | 4 | 4 | 4 | - | 0.28 | 0.41 | 0.28 | 0.55 | 0.28 | 0.69 | 0.55 | 0.41 | 0.55 | 0.28 |
| 64. SMT64 | 4 | 4 | 4 | 2 | - | 0.41 | 0.28 | 0.55 | 0.28 | 0.69 | 0.55 | 0.41 | 0.55 | 0.28 |
| 65. SMT65 | 5 | 5 | 5 | 3 | 3 | - | 0.41 | 0.69 | 0.41 | 0.83 | 0.69 | 0.55 | 0.69 | 0.41 |
| 66. SMT66 | 4 | 4 | 4 | 2 | 2 | 3 | - | 0.55 | 0.28 | 0.69 | 0.55 | 0.41 | 0.55 | 0.28 |
| 67. SMT67 | 6 | 2 | 6 | 4 | 4 | 5 | 4 | - | 0.55 | 0.96 | 0.83 | 0.69 | 0.83 | 0.55 |
| 68. SMT68 | 4 | 4 | 4 | 2 | 2 | 3 | 2 | 4 | - | 0.69 | 0.55 | 0.41 | 0.55 | 0.28 |
| 69. SMT69 | 7 | 7 | 7 | 5 | 5 | 6 | 5 | 7 | 5 | - | 0.96 | 0.83 | 0.96 | 0.69 |
| 70. SMT70 | 6 | 6 | 6 | 4 | 4 | 5 | 4 | 6 | 4 | 7 | - | 0.69 | 0.83 | 0.55 |
| 71. SMT71 | 5 | 5 | 5 | 3 | 3 | 4 | 3 | 5 | 3 | 6 | 5 | - | 0.41 | 0.41 |
| 72. SMT72 | 4 | 6 | 6 | 4 | 4 | 5 | 4 | 6 | 4 | 7 | 6 | 3 | - | 0.55 |
| 73. SMT73 | 4 | 4 | 4 | 2 | 2 | 3 | 2 | 4 | 2 | 5 | 4 | 3 | 4 | - |

Numbers above the diagonal are percent distance values; numbers below the diagonal are absolute distance values.
