# Supplementary material for: Identification of Sympetrum depressiusculum Sélys, 1841 in South Korea (Odonata: Libellulidae) According to Morphology and Genetic Markers
Source: Insects. 2023 Aug 30;14(9):733. doi: 10.3390/insects14090733 (PMC10531817; doi:10.3390/insects14090733)
Supplement: Supplementary file 1 [file insects-14-00733-s001.zip › Table S11. PC-Our ITS+GB ITS.docx]

**Table S11.** Pairwise comparisons of ITS haplotypes of *Sympetrum* species sequenced in this study and collected from public data.

| Haplotype | 1 | 2 | 3 | 4 | 5 | 6 | 7 | 8 | 9 | 10 | 11 | 12 | 13 | 14 |
| --- | --- | --- | --- | --- | --- | --- | --- | --- | --- | --- | --- | --- | --- | --- |
| 1. SITS01 | - | 0.27 | 0.80 | 0.40 | 0.40 | 0.13 | 0.13 | 0.13 | 0.27 | 0.27 | 0.27 | 0.54 | 0.27 | 0.13 |
| 2. SITS02 | 2 | - | 1.07 | 0.67 | 0.67 | 0.40 | 0.40 | 0.40 | 0.54 | 0.54 | 0.54 | 0.80 | 0.54 | 0.40 |
| 3. SITS04 | 6 | 8 | - | 0.67 | 0.67 | 0.94 | 0.94 | 0.94 | 0.54 | 1.07 | 1.07 | 1.07 | 1.07 | 0.94 |
| 4. SITS05 | 3 | 5 | 5 | - | 0.27 | 0.54 | 0.54 | 0.54 | 0.13 | 0.67 | 0.67 | 0.67 | 0.67 | 0.54 |
| 5. SITS06 | 3 | 5 | 5 | 2 | - | 0.54 | 0.54 | 0.54 | 0.13 | 0.67 | 0.67 | 0.67 | 0.67 | 0.54 |
| 6. SITS07 | 1 | 3 | 7 | 4 | 4 | - | 0.27 | 0.27 | 0.40 | 0.40 | 0.40 | 0.67 | 0.40 | 0.27 |
| 7. SITS08 | 1 | 3 | 7 | 4 | 4 | 2 | - | 0.27 | 0.40 | 0.40 | 0.40 | 0.67 | 0.40 | 0.27 |
| 8. SITS09 | 1 | 3 | 7 | 4 | 4 | 2 | 2 | - | 0.40 | 0.40 | 0.40 | 0.67 | 0.40 | 0.27 |
| 9. SITS10 | 2 | 4 | 4 | 1 | 1 | 3 | 3 | 3 | - | 0.54 | 0.54 | 0.54 | 0.54 | 0.40 |
| 10. SITS11 | 2 | 4 | 8 | 5 | 5 | 3 | 3 | 3 | 4 | - | 0.54 | 0.67 | 0.54 | 0.40 |
| 11. SITS13 | 2 | 4 | 8 | 5 | 5 | 3 | 3 | 3 | 4 | 4 | - | 0.80 | 0.54 | 0.40 |
| 12. SITS14 | 4 | 6 | 8 | 5 | 5 | 5 | 5 | 5 | 4 | 5 | 6 | - | 0.80 | 0.67 |
| 13. SITS15 | 2 | 4 | 8 | 5 | 5 | 3 | 3 | 3 | 4 | 4 | 4 | 6 | - | 0.40 |
| 14. SITS16 | 1 | 3 | 7 | 4 | 4 | 2 | 2 | 2 | 3 | 3 | 3 | 5 | 3 | - |
| 15. SITS17 | 1 | 3 | 7 | 4 | 4 | 2 | 2 | 2 | 3 | 3 | 3 | 5 | 3 | 2 |
| 16. SITS18 | 1 | 3 | 7 | 4 | 4 | 2 | 2 | 2 | 3 | 3 | 3 | 5 | 3 | 2 |
| 17. SITS19 | 3 | 5 | 9 | 6 | 6 | 4 | 4 | 4 | 5 | 4 | 5 | 7 | 5 | 4 |
| 18. SITS20 | 4 | 6 | 10 | 7 | 7 | 5 | 5 | 5 | 6 | 6 | 6 | 8 | 6 | 5 |
| 19. SITS21 | 1 | 3 | 7 | 4 | 4 | 2 | 2 | 2 | 3 | 3 | 3 | 5 | 3 | 2 |
| 20. SITS22 | 1 | 3 | 7 | 4 | 4 | 2 | 2 | 2 | 3 | 3 | 3 | 5 | 3 | 2 |
| 21. SITS23 | 1 | 3 | 5 | 2 | 2 | 2 | 2 | 2 | 1 | 3 | 3 | 3 | 3 | 2 |
| 22. SITS24 | 2 | 4 | 8 | 5 | 5 | 3 | 3 | 3 | 4 | 4 | 4 | 6 | 4 | 3 |
| 23. SITS25 | 6 | 8 | 8 | 5 | 5 | 7 | 7 | 7 | 4 | 8 | 8 | 8 | 8 | 7 |
| 24. SITS26 | 6 | 8 | 7 | 5 | 5 | 7 | 7 | 7 | 4 | 8 | 8 | 8 | 8 | 7 |
| 25. SITS28 | 4 | 6 | 6 | 3 | 3 | 5 | 5 | 5 | 2 | 6 | 6 | 6 | 6 | 5 |
| 26. SITS29 | 4 | 6 | 6 | 3 | 3 | 5 | 5 | 5 | 2 | 6 | 6 | 6 | 6 | 5 |
| 27. SITS30 | 3 | 5 | 5 | 2 | 2 | 4 | 4 | 4 | 1 | 5 | 5 | 5 | 5 | 4 |
| 28. SITS31 | 3 | 5 | 5 | 2 | 2 | 4 | 4 | 4 | 1 | 5 | 5 | 5 | 5 | 4 |
| 29. SITS32 | 4 | 6 | 6 | 3 | 3 | 5 | 5 | 5 | 2 | 6 | 6 | 6 | 6 | 5 |
| 30. SITS33 | 8 | 10 | 9 | 7 | 7 | 9 | 9 | 9 | 6 | 10 | 10 | 10 | 10 | 9 |
| 31. SITS34 | 5 | 7 | 7 | 4 | 4 | 6 | 6 | 6 | 3 | 7 | 7 | 7 | 7 | 6 |
| 32. SITS35 | 4 | 6 | 6 | 3 | 3 | 5 | 5 | 5 | 2 | 6 | 6 | 6 | 6 | 5 |
| 33. SITS36 | 3 | 5 | 5 | 2 | 2 | 4 | 4 | 4 | 1 | 5 | 5 | 5 | 5 | 4 |
| 34. SITS37 | 3 | 5 | 5 | 2 | 2 | 4 | 4 | 4 | 1 | 5 | 5 | 5 | 5 | 4 |
| 35. SITS38 | 3 | 5 | 5 | 2 | 2 | 4 | 4 | 4 | 1 | 5 | 5 | 5 | 5 | 4 |
| 36. SITS39 | 3 | 5 | 5 | 2 | 2 | 4 | 4 | 4 | 1 | 5 | 5 | 5 | 5 | 4 |
| 37. SITS40 | 5 | 7 | 7 | 4 | 4 | 6 | 6 | 6 | 3 | 7 | 7 | 7 | 7 | 6 |
| 38. SITS41 | 3 | 5 | 5 | 2 | 2 | 4 | 4 | 4 | 1 | 5 | 5 | 5 | 5 | 4 |
| 39. SITS42 | 4 | 6 | 6 | 3 | 3 | 5 | 5 | 5 | 2 | 6 | 6 | 6 | 6 | 5 |
| 40. SITS43 | 4 | 6 | 6 | 3 | 3 | 5 | 5 | 5 | 2 | 6 | 6 | 6 | 6 | 5 |
| 41. SITS44 | 3 | 5 | 5 | 2 | 2 | 4 | 4 | 4 | 1 | 5 | 5 | 5 | 5 | 4 |
| 42. SITS45 | 3 | 5 | 5 | 2 | 2 | 4 | 4 | 4 | 1 | 5 | 5 | 5 | 5 | 4 |
| 43. SITS46 | 6 | 8 | 8 | 5 | 5 | 7 | 7 | 7 | 4 | 8 | 8 | 8 | 8 | 7 |
| 44. SITS47 | 4 | 6 | 6 | 3 | 3 | 5 | 5 | 5 | 2 | 6 | 6 | 6 | 6 | 5 |
| 45. SITS48 | 3 | 5 | 5 | 2 | 2 | 4 | 4 | 4 | 1 | 5 | 5 | 5 | 5 | 4 |
| 46. SITS49 | 3 | 5 | 5 | 2 | 2 | 4 | 4 | 4 | 1 | 5 | 5 | 5 | 5 | 4 |
| 47. SITS50 | 1 | 3 | 7 | 4 | 4 | 2 | 2 | 2 | 3 | 3 | 3 | 5 | 3 | 2 |
| 48. SITS51 | 1 | 3 | 7 | 4 | 4 | 2 | 2 | 2 | 3 | 3 | 3 | 5 | 3 | 2 |
| 49. SITS52 | 3 | 5 | 9 | 6 | 6 | 4 | 4 | 4 | 5 | 5 | 5 | 7 | 5 | 4 |
| 50. SITS53 | 2 | 4 | 8 | 5 | 5 | 3 | 3 | 3 | 4 | 4 | 4 | 6 | 4 | 3 |
| 51. SITS55 | 3 | 5 | 7 | 4 | 4 | 4 | 4 | 4 | 3 | 5 | 5 | 7 | 5 | 4 |
| 52. SITS56 | 3 | 5 | 5 | 2 | 2 | 4 | 4 | 4 | 1 | 5 | 5 | 5 | 5 | 4 |
| 53. SITS57 | 1 | 3 | 7 | 4 | 4 | 2 | 2 | 2 | 3 | 3 | 3 | 5 | 3 | 2 |
| 54. SITS58 | 5 | 7 | 7 | 4 | 4 | 6 | 6 | 6 | 3 | 7 | 7 | 7 | 7 | 6 |
| 55. SITS59 | 3 | 5 | 5 | 2 | 2 | 4 | 4 | 4 | 1 | 5 | 5 | 5 | 5 | 4 |
| 56. SITS60 | 1 | 3 | 7 | 4 | 4 | 2 | 2 | 2 | 3 | 3 | 3 | 5 | 3 | 2 |

| Haplotype | 15 | 16 | 17 | 18 | 19 | 20 | 21 | 22 | 23 | 24 | 25 | 26 | 27 | 28 |
| --- | --- | --- | --- | --- | --- | --- | --- | --- | --- | --- | --- | --- | --- | --- |
| 1. SITS01 | 0.13 | 0.13 | 0.40 | 0.54 | 0.13 | 0.13 | 0.13 | 0.27 | 0.80 | 0.80 | 0.54 | 0.54 | 0.40 | 0.40 |
| 2. SITS02 | 0.40 | 0.40 | 0.67 | 0.80 | 0.40 | 0.40 | 0.40 | 0.54 | 1.07 | 1.07 | 0.80 | 0.80 | 0.67 | 0.67 |
| 3. SITS04 | 0.94 | 0.94 | 1.21 | 1.34 | 0.94 | 0.94 | 0.67 | 1.07 | 1.07 | 0.94 | 0.80 | 0.80 | 0.67 | 0.67 |
| 4. SITS05 | 0.54 | 0.54 | 0.80 | 0.94 | 0.54 | 0.54 | 0.27 | 0.67 | 0.67 | 0.67 | 0.40 | 0.40 | 0.27 | 0.27 |
| 5. SITS06 | 0.54 | 0.54 | 0.80 | 0.94 | 0.54 | 0.54 | 0.27 | 0.67 | 0.67 | 0.67 | 0.40 | 0.40 | 0.27 | 0.27 |
| 6. SITS07 | 0.27 | 0.27 | 0.54 | 0.67 | 0.27 | 0.27 | 0.27 | 0.40 | 0.94 | 0.94 | 0.67 | 0.67 | 0.54 | 0.54 |
| 7. SITS08 | 0.27 | 0.27 | 0.54 | 0.67 | 0.27 | 0.27 | 0.27 | 0.40 | 0.94 | 0.94 | 0.67 | 0.67 | 0.54 | 0.54 |
| 8. SITS09 | 0.27 | 0.27 | 0.54 | 0.67 | 0.27 | 0.27 | 0.27 | 0.40 | 0.94 | 0.94 | 0.67 | 0.67 | 0.54 | 0.54 |
| 9. SITS10 | 0.40 | 0.40 | 0.67 | 0.80 | 0.40 | 0.40 | 0.13 | 0.54 | 0.54 | 0.54 | 0.27 | 0.27 | 0.13 | 0.13 |
| 10. SITS11 | 0.40 | 0.40 | 0.54 | 0.80 | 0.40 | 0.40 | 0.40 | 0.54 | 1.07 | 1.07 | 0.80 | 0.80 | 0.67 | 0.67 |
| 11. SITS13 | 0.40 | 0.40 | 0.67 | 0.80 | 0.40 | 0.40 | 0.40 | 0.54 | 1.07 | 1.07 | 0.80 | 0.80 | 0.67 | 0.67 |
| 12. SITS14 | 0.67 | 0.67 | 0.94 | 1.07 | 0.67 | 0.67 | 0.40 | 0.80 | 1.07 | 1.07 | 0.80 | 0.80 | 0.67 | 0.67 |
| 13. SITS15 | 0.40 | 0.40 | 0.67 | 0.80 | 0.40 | 0.40 | 0.40 | 0.54 | 1.07 | 1.07 | 0.80 | 0.80 | 0.67 | 0.67 |
| 14. SITS16 | 0.27 | 0.27 | 0.54 | 0.67 | 0.27 | 0.27 | 0.27 | 0.40 | 0.94 | 0.94 | 0.67 | 0.67 | 0.54 | 0.54 |
| 15. SITS17 | - | 0.27 | 0.54 | 0.67 | 0.27 | 0.27 | 0.27 | 0.40 | 0.94 | 0.94 | 0.67 | 0.67 | 0.54 | 0.54 |
| 16. SITS18 | 2 | - | 0.54 | 0.67 | 0.27 | 0.27 | 0.27 | 0.40 | 0.94 | 0.94 | 0.67 | 0.67 | 0.54 | 0.54 |
| 17. SITS19 | 4 | 4 | - | 0.67 | 0.54 | 0.54 | 0.54 | 0.67 | 1.21 | 1.21 | 0.94 | 0.94 | 0.80 | 0.80 |
| 18. SITS20 | 5 | 5 | 5 | - | 0.67 | 0.67 | 0.67 | 0.80 | 1.34 | 1.34 | 1.07 | 1.07 | 0.94 | 0.94 |
| 19. SITS21 | 2 | 2 | 4 | 5 | - | 0.27 | 0.27 | 0.40 | 0.94 | 0.94 | 0.67 | 0.67 | 0.54 | 0.54 |
| 20. SITS22 | 2 | 2 | 4 | 5 | 2 | - | 0.27 | 0.40 | 0.94 | 0.94 | 0.67 | 0.67 | 0.54 | 0.54 |
| 21. SITS23 | 2 | 2 | 4 | 5 | 2 | 2 | - | 0.40 | 0.67 | 0.67 | 0.40 | 0.40 | 0.27 | 0.27 |
| 22. SITS24 | 3 | 3 | 5 | 6 | 3 | 3 | 3 | - | 1.07 | 1.07 | 0.80 | 0.80 | 0.67 | 0.67 |
| 23. SITS25 | 7 | 7 | 9 | 10 | 7 | 7 | 5 | 8 | - | 1.07 | 0.80 | 0.80 | 0.67 | 0.67 |
| 24. SITS26 | 7 | 7 | 9 | 10 | 7 | 7 | 5 | 8 | 8 | - | 0.80 | 0.80 | 0.67 | 0.67 |
| 25. SITS28 | 5 | 5 | 7 | 8 | 5 | 5 | 3 | 6 | 6 | 6 | - | 0.27 | 0.40 | 0.13 |
| 26. SITS29 | 5 | 5 | 7 | 8 | 5 | 5 | 3 | 6 | 6 | 6 | 2 | - | 0.40 | 0.13 |
| 27. SITS30 | 4 | 4 | 6 | 7 | 4 | 4 | 2 | 5 | 5 | 5 | 3 | 3 | - | 0.27 |
| 28. SITS31 | 4 | 4 | 6 | 7 | 4 | 4 | 2 | 5 | 5 | 5 | 1 | 1 | 2 | - |
| 29. SITS32 | 5 | 5 | 7 | 8 | 5 | 5 | 3 | 6 | 6 | 6 | 4 | 4 | 3 | 3 |
| 30. SITS33 | 9 | 9 | 11 | 12 | 9 | 9 | 7 | 10 | 10 | 2 | 8 | 8 | 7 | 7 |
| 31. SITS34 | 6 | 6 | 8 | 9 | 6 | 6 | 4 | 7 | 7 | 7 | 5 | 5 | 4 | 4 |
| 32. SITS35 | 5 | 5 | 7 | 8 | 5 | 5 | 3 | 6 | 6 | 6 | 4 | 4 | 3 | 3 |
| 33. SITS36 | 4 | 4 | 6 | 7 | 4 | 4 | 2 | 5 | 5 | 5 | 3 | 3 | 2 | 2 |
| 34. SITS37 | 4 | 4 | 6 | 7 | 4 | 4 | 2 | 5 | 5 | 5 | 3 | 3 | 2 | 2 |
| 35. SITS38 | 4 | 4 | 6 | 7 | 4 | 4 | 2 | 5 | 5 | 5 | 3 | 3 | 2 | 2 |
| 36. SITS39 | 4 | 4 | 6 | 7 | 4 | 4 | 2 | 5 | 5 | 5 | 3 | 3 | 2 | 2 |
| 37. SITS40 | 6 | 6 | 8 | 9 | 6 | 6 | 4 | 7 | 7 | 7 | 5 | 5 | 4 | 4 |
| 38. SITS41 | 4 | 4 | 6 | 7 | 4 | 4 | 2 | 5 | 5 | 5 | 3 | 3 | 2 | 2 |
| 39. SITS42 | 5 | 5 | 7 | 8 | 5 | 5 | 3 | 6 | 6 | 6 | 4 | 4 | 3 | 3 |
| 40. SITS43 | 5 | 5 | 7 | 8 | 5 | 5 | 3 | 6 | 6 | 6 | 4 | 4 | 3 | 3 |
| 41. SITS44 | 4 | 4 | 6 | 7 | 4 | 4 | 2 | 5 | 5 | 5 | 3 | 3 | 2 | 2 |
| 42. SITS45 | 4 | 4 | 6 | 7 | 4 | 4 | 2 | 5 | 5 | 5 | 3 | 3 | 2 | 2 |
| 43. SITS46 | 7 | 7 | 9 | 10 | 7 | 7 | 5 | 8 | 8 | 8 | 6 | 6 | 5 | 5 |
| 44. SITS47 | 5 | 5 | 7 | 8 | 5 | 5 | 3 | 6 | 4 | 6 | 4 | 4 | 3 | 3 |
| 45. SITS48 | 4 | 4 | 6 | 7 | 2 | 4 | 2 | 5 | 5 | 5 | 3 | 3 | 2 | 2 |
| 46. SITS49 | 4 | 4 | 6 | 7 | 4 | 4 | 2 | 5 | 5 | 5 | 3 | 3 | 2 | 2 |
| 47. SITS50 | 2 | 2 | 4 | 5 | 2 | 2 | 2 | 3 | 7 | 7 | 5 | 5 | 4 | 4 |
| 48. SITS51 | 2 | 2 | 4 | 5 | 2 | 2 | 2 | 3 | 7 | 7 | 5 | 5 | 4 | 4 |
| 49. SITS52 | 4 | 4 | 6 | 5 | 4 | 4 | 4 | 5 | 9 | 9 | 7 | 7 | 6 | 6 |
| 50. SITS53 | 3 | 3 | 5 | 6 | 3 | 3 | 3 | 4 | 8 | 8 | 6 | 6 | 5 | 5 |
| 51. SITS55 | 4 | 4 | 6 | 7 | 4 | 4 | 4 | 5 | 7 | 7 | 5 | 5 | 4 | 4 |
| 52. SITS56 | 4 | 4 | 4 | 5 | 4 | 4 | 2 | 5 | 5 | 5 | 3 | 3 | 2 | 2 |
| 53. SITS57 | 2 | 2 | 2 | 3 | 2 | 2 | 2 | 3 | 7 | 7 | 5 | 5 | 4 | 4 |
| 54. SITS58 | 6 | 6 | 8 | 9 | 6 | 6 | 4 | 7 | 7 | 7 | 5 | 5 | 4 | 4 |
| 55. SITS59 | 4 | 4 | 6 | 7 | 4 | 4 | 2 | 5 | 5 | 5 | 3 | 3 | 2 | 2 |
| 56. SITS60 | 2 | 2 | 4 | 5 | 2 | 2 | 2 | 3 | 7 | 7 | 5 | 5 | 4 | 4 |

| Haplotype | 29 | 30 | 31 | 32 | 33 | 34 | 35 | 36 | 37 | 38 | 39 | 40 | 41 | 42 |
| --- | --- | --- | --- | --- | --- | --- | --- | --- | --- | --- | --- | --- | --- | --- |
| 1. SITS01 | 0.54 | 1.07 | 0.67 | 0.54 | 0.40 | 0.40 | 0.40 | 0.40 | 0.67 | 0.40 | 0.54 | 0.54 | 0.40 | 0.40 |
| 2. SITS02 | 0.80 | 1.34 | 0.94 | 0.80 | 0.67 | 0.67 | 0.67 | 0.67 | 0.94 | 0.67 | 0.80 | 0.80 | 0.67 | 0.67 |
| 3. SITS04 | 0.80 | 1.21 | 0.94 | 0.80 | 0.67 | 0.67 | 0.67 | 0.67 | 0.94 | 0.67 | 0.80 | 0.80 | 0.67 | 0.67 |
| 4. SITS05 | 0.40 | 0.94 | 0.54 | 0.40 | 0.27 | 0.27 | 0.27 | 0.27 | 0.54 | 0.27 | 0.40 | 0.40 | 0.27 | 0.27 |
| 5. SITS06 | 0.40 | 0.94 | 0.54 | 0.40 | 0.27 | 0.27 | 0.27 | 0.27 | 0.54 | 0.27 | 0.40 | 0.40 | 0.27 | 0.27 |
| 6. SITS07 | 0.67 | 1.21 | 0.80 | 0.67 | 0.54 | 0.54 | 0.54 | 0.54 | 0.80 | 0.54 | 0.67 | 0.67 | 0.54 | 0.54 |
| 7. SITS08 | 0.67 | 1.21 | 0.80 | 0.67 | 0.54 | 0.54 | 0.54 | 0.54 | 0.80 | 0.54 | 0.67 | 0.67 | 0.54 | 0.54 |
| 8. SITS09 | 0.67 | 1.21 | 0.80 | 0.67 | 0.54 | 0.54 | 0.54 | 0.54 | 0.80 | 0.54 | 0.67 | 0.67 | 0.54 | 0.54 |
| 9. SITS10 | 0.27 | 0.80 | 0.40 | 0.27 | 0.13 | 0.13 | 0.13 | 0.13 | 0.40 | 0.13 | 0.27 | 0.27 | 0.13 | 0.13 |
| 10. SITS11 | 0.80 | 1.34 | 0.94 | 0.80 | 0.67 | 0.67 | 0.67 | 0.67 | 0.94 | 0.67 | 0.80 | 0.80 | 0.67 | 0.67 |
| 11. SITS13 | 0.80 | 1.34 | 0.94 | 0.80 | 0.67 | 0.67 | 0.67 | 0.67 | 0.94 | 0.67 | 0.80 | 0.80 | 0.67 | 0.67 |
| 12. SITS14 | 0.80 | 1.34 | 0.94 | 0.80 | 0.67 | 0.67 | 0.67 | 0.67 | 0.94 | 0.67 | 0.80 | 0.80 | 0.67 | 0.67 |
| 13. SITS15 | 0.80 | 1.34 | 0.94 | 0.80 | 0.67 | 0.67 | 0.67 | 0.67 | 0.94 | 0.67 | 0.80 | 0.80 | 0.67 | 0.67 |
| 14. SITS16 | 0.67 | 1.21 | 0.80 | 0.67 | 0.54 | 0.54 | 0.54 | 0.54 | 0.80 | 0.54 | 0.67 | 0.67 | 0.54 | 0.54 |
| 15. SITS17 | 0.67 | 1.21 | 0.80 | 0.67 | 0.54 | 0.54 | 0.54 | 0.54 | 0.80 | 0.54 | 0.67 | 0.67 | 0.54 | 0.54 |
| 16. SITS18 | 0.67 | 1.21 | 0.80 | 0.67 | 0.54 | 0.54 | 0.54 | 0.54 | 0.80 | 0.54 | 0.67 | 0.67 | 0.54 | 0.54 |
| 17. SITS19 | 0.94 | 1.48 | 1.07 | 0.94 | 0.80 | 0.80 | 0.80 | 0.80 | 1.07 | 0.80 | 0.94 | 0.94 | 0.80 | 0.80 |
| 18. SITS20 | 1.07 | 1.61 | 1.21 | 1.07 | 0.94 | 0.94 | 0.94 | 0.94 | 1.21 | 0.94 | 1.07 | 1.07 | 0.94 | 0.94 |
| 19. SITS21 | 0.67 | 1.21 | 0.80 | 0.67 | 0.54 | 0.54 | 0.54 | 0.54 | 0.80 | 0.54 | 0.67 | 0.67 | 0.54 | 0.54 |
| 20. SITS22 | 0.67 | 1.21 | 0.80 | 0.67 | 0.54 | 0.54 | 0.54 | 0.54 | 0.80 | 0.54 | 0.67 | 0.67 | 0.54 | 0.54 |
| 21. SITS23 | 0.40 | 0.94 | 0.54 | 0.40 | 0.27 | 0.27 | 0.27 | 0.27 | 0.54 | 0.27 | 0.40 | 0.40 | 0.27 | 0.27 |
| 22. SITS24 | 0.80 | 1.34 | 0.94 | 0.80 | 0.67 | 0.67 | 0.67 | 0.67 | 0.94 | 0.67 | 0.80 | 0.80 | 0.67 | 0.67 |
| 23. SITS25 | 0.80 | 1.34 | 0.94 | 0.80 | 0.67 | 0.67 | 0.67 | 0.67 | 0.94 | 0.67 | 0.80 | 0.80 | 0.67 | 0.67 |
| 24. SITS26 | 0.80 | 0.27 | 0.94 | 0.80 | 0.67 | 0.67 | 0.67 | 0.67 | 0.94 | 0.67 | 0.80 | 0.80 | 0.67 | 0.67 |
| 25. SITS28 | 0.54 | 1.07 | 0.67 | 0.54 | 0.40 | 0.40 | 0.40 | 0.40 | 0.67 | 0.40 | 0.54 | 0.54 | 0.40 | 0.40 |
| 26. SITS29 | 0.54 | 1.07 | 0.67 | 0.54 | 0.40 | 0.40 | 0.40 | 0.40 | 0.67 | 0.40 | 0.54 | 0.54 | 0.40 | 0.40 |
| 27. SITS30 | 0.40 | 0.94 | 0.54 | 0.40 | 0.27 | 0.27 | 0.27 | 0.27 | 0.54 | 0.27 | 0.40 | 0.40 | 0.27 | 0.27 |
| 28. SITS31 | 0.40 | 0.94 | 0.54 | 0.40 | 0.27 | 0.27 | 0.27 | 0.27 | 0.54 | 0.27 | 0.40 | 0.40 | 0.27 | 0.27 |
| 29. SITS32 | - | 1.07 | 0.67 | 0.54 | 0.40 | 0.40 | 0.40 | 0.27 | 0.67 | 0.40 | 0.54 | 0.54 | 0.40 | 0.40 |
| 30. SITS33 | 8 | - | 1.21 | 1.07 | 0.94 | 0.94 | 0.94 | 0.94 | 1.21 | 0.94 | 1.07 | 1.07 | 0.94 | 0.94 |
| 31. SITS34 | 5 | 9 | - | 0.67 | 0.54 | 0.54 | 0.54 | 0.54 | 0.80 | 0.54 | 0.67 | 0.67 | 0.54 | 0.54 |
| 32. SITS35 | 4 | 8 | 5 | - | 0.40 | 0.13 | 0.40 | 0.40 | 0.40 | 0.40 | 0.27 | 0.27 | 0.40 | 0.40 |
| 33. SITS36 | 3 | 7 | 4 | 3 | - | 0.27 | 0.27 | 0.27 | 0.54 | 0.27 | 0.40 | 0.40 | 0.27 | 0.27 |
| 34. SITS37 | 3 | 7 | 4 | 1 | 2 | - | 0.27 | 0.27 | 0.27 | 0.27 | 0.13 | 0.13 | 0.27 | 0.27 |
| 35. SITS38 | 3 | 7 | 4 | 3 | 2 | 2 | - | 0.27 | 0.54 | 0.27 | 0.40 | 0.40 | 0.27 | 0.27 |
| 36. SITS39 | 2 | 7 | 4 | 3 | 2 | 2 | 2 | - | 0.54 | 0.27 | 0.40 | 0.40 | 0.27 | 0.27 |
| 37. SITS40 | 5 | 9 | 6 | 3 | 4 | 2 | 4 | 4 | - | 0.54 | 0.40 | 0.40 | 0.54 | 0.54 |
| 38. SITS41 | 3 | 7 | 4 | 3 | 2 | 2 | 2 | 2 | 4 | - | 0.40 | 0.40 | 0.27 | 0.27 |
| 39. SITS42 | 4 | 8 | 5 | 2 | 3 | 1 | 3 | 3 | 3 | 3 | - | 0.27 | 0.40 | 0.40 |
| 40. SITS43 | 4 | 8 | 5 | 2 | 3 | 1 | 3 | 3 | 3 | 3 | 2 | - | 0.40 | 0.40 |
| 41. SITS44 | 3 | 7 | 4 | 3 | 2 | 2 | 2 | 2 | 4 | 2 | 3 | 3 | - | 0.27 |
| 42. SITS45 | 3 | 7 | 4 | 3 | 2 | 2 | 2 | 2 | 4 | 2 | 3 | 3 | 2 | - |
| 43. SITS46 | 6 | 10 | 7 | 4 | 5 | 3 | 5 | 5 | 5 | 5 | 4 | 4 | 5 | 5 |
| 44. SITS47 | 4 | 8 | 5 | 4 | 3 | 3 | 3 | 3 | 5 | 3 | 4 | 4 | 3 | 3 |
| 45. SITS48 | 3 | 7 | 4 | 3 | 2 | 2 | 2 | 2 | 4 | 2 | 3 | 3 | 2 | 2 |
| 46. SITS49 | 3 | 7 | 4 | 3 | 2 | 2 | 2 | 2 | 4 | 2 | 3 | 3 | 2 | 2 |
| 47. SITS50 | 5 | 9 | 6 | 5 | 4 | 4 | 4 | 4 | 6 | 4 | 5 | 5 | 4 | 4 |
| 48. SITS51 | 5 | 9 | 6 | 5 | 4 | 4 | 4 | 4 | 6 | 4 | 5 | 5 | 4 | 4 |
| 49. SITS52 | 7 | 11 | 8 | 7 | 6 | 6 | 6 | 6 | 8 | 6 | 7 | 7 | 6 | 6 |
| 50. SITS53 | 6 | 10 | 7 | 6 | 5 | 5 | 5 | 5 | 7 | 5 | 6 | 6 | 5 | 5 |
| 51. SITS55 | 5 | 9 | 6 | 5 | 4 | 4 | 4 | 4 | 6 | 4 | 5 | 5 | 4 | 4 |
| 52. SITS56 | 3 | 7 | 4 | 3 | 2 | 2 | 2 | 2 | 4 | 2 | 3 | 3 | 2 | 2 |
| 53. SITS57 | 5 | 9 | 6 | 5 | 4 | 4 | 4 | 4 | 6 | 4 | 5 | 5 | 4 | 4 |
| 54. SITS58 | 5 | 9 | 6 | 5 | 4 | 4 | 4 | 4 | 6 | 4 | 5 | 5 | 4 | 4 |
| 55. SITS59 | 3 | 7 | 4 | 3 | 2 | 2 | 2 | 2 | 4 | 2 | 3 | 3 | 2 | 2 |
| 56. SITS60 | 5 | 9 | 6 | 5 | 4 | 4 | 4 | 4 | 6 | 4 | 5 | 5 | 4 | 4 |

| Haplotype | 43 | 44 | 45 | 46 | 47 | 48 | 49 | 50 | 51 | 52 | 53 | 54 | 55 | 56 |
| --- | --- | --- | --- | --- | --- | --- | --- | --- | --- | --- | --- | --- | --- | --- |
| 1. SITS01 | 0.80 | 0.54 | 0.40 | 0.40 | 0.13 | 0.13 | 0.40 | 0.27 | 0.40 | 0.40 | 0.13 | 0.67 | 0.40 | 0.13 |
| 2. SITS02 | 1.07 | 0.80 | 0.67 | 0.67 | 0.40 | 0.40 | 0.67 | 0.54 | 0.67 | 0.67 | 0.40 | 0.94 | 0.67 | 0.40 |
| 3. SITS04 | 1.07 | 0.80 | 0.67 | 0.67 | 0.94 | 0.94 | 1.21 | 1.07 | 0.94 | 0.67 | 0.94 | 0.94 | 0.67 | 0.94 |
| 4. SITS05 | 0.67 | 0.40 | 0.27 | 0.27 | 0.54 | 0.54 | 0.80 | 0.67 | 0.54 | 0.27 | 0.54 | 0.54 | 0.27 | 0.54 |
| 5. SITS06 | 0.67 | 0.40 | 0.27 | 0.27 | 0.54 | 0.54 | 0.80 | 0.67 | 0.54 | 0.27 | 0.54 | 0.54 | 0.27 | 0.54 |
| 6. SITS07 | 0.94 | 0.67 | 0.54 | 0.54 | 0.27 | 0.27 | 0.54 | 0.40 | 0.54 | 0.54 | 0.27 | 0.80 | 0.54 | 0.27 |
| 7. SITS08 | 0.94 | 0.67 | 0.54 | 0.54 | 0.27 | 0.27 | 0.54 | 0.40 | 0.54 | 0.54 | 0.27 | 0.80 | 0.54 | 0.27 |
| 8. SITS09 | 0.94 | 0.67 | 0.54 | 0.54 | 0.27 | 0.27 | 0.54 | 0.40 | 0.54 | 0.54 | 0.27 | 0.80 | 0.54 | 0.27 |
| 9. SITS10 | 0.54 | 0.27 | 0.13 | 0.13 | 0.40 | 0.40 | 0.67 | 0.54 | 0.40 | 0.13 | 0.40 | 0.40 | 0.13 | 0.40 |
| 10. SITS11 | 1.07 | 0.80 | 0.67 | 0.67 | 0.40 | 0.40 | 0.67 | 0.54 | 0.67 | 0.67 | 0.40 | 0.94 | 0.67 | 0.40 |
| 11. SITS13 | 1.07 | 0.80 | 0.67 | 0.67 | 0.40 | 0.40 | 0.67 | 0.54 | 0.67 | 0.67 | 0.40 | 0.94 | 0.67 | 0.40 |
| 12. SITS14 | 1.07 | 0.80 | 0.67 | 0.67 | 0.67 | 0.67 | 0.94 | 0.80 | 0.94 | 0.67 | 0.67 | 0.94 | 0.67 | 0.67 |
| 13. SITS15 | 1.07 | 0.80 | 0.67 | 0.67 | 0.40 | 0.40 | 0.67 | 0.54 | 0.67 | 0.67 | 0.40 | 0.94 | 0.67 | 0.40 |
| 14. SITS16 | 0.94 | 0.67 | 0.54 | 0.54 | 0.27 | 0.27 | 0.54 | 0.40 | 0.54 | 0.54 | 0.27 | 0.80 | 0.54 | 0.27 |
| 15. SITS17 | 0.94 | 0.67 | 0.54 | 0.54 | 0.27 | 0.27 | 0.54 | 0.40 | 0.54 | 0.54 | 0.27 | 0.80 | 0.54 | 0.27 |
| 16. SITS18 | 0.94 | 0.67 | 0.54 | 0.54 | 0.27 | 0.27 | 0.54 | 0.40 | 0.54 | 0.54 | 0.27 | 0.80 | 0.54 | 0.27 |
| 17. SITS19 | 1.21 | 0.94 | 0.80 | 0.80 | 0.54 | 0.54 | 0.80 | 0.67 | 0.80 | 0.54 | 0.27 | 1.07 | 0.80 | 0.54 |
| 18. SITS20 | 1.34 | 1.07 | 0.94 | 0.94 | 0.67 | 0.67 | 0.67 | 0.80 | 0.94 | 0.67 | 0.40 | 1.21 | 0.94 | 0.67 |
| 19. SITS21 | 0.94 | 0.67 | 0.27 | 0.54 | 0.27 | 0.27 | 0.54 | 0.40 | 0.54 | 0.54 | 0.27 | 0.80 | 0.54 | 0.27 |
| 20. SITS22 | 0.94 | 0.67 | 0.54 | 0.54 | 0.27 | 0.27 | 0.54 | 0.40 | 0.54 | 0.54 | 0.27 | 0.80 | 0.54 | 0.27 |
| 21. SITS23 | 0.67 | 0.40 | 0.27 | 0.27 | 0.27 | 0.27 | 0.54 | 0.40 | 0.54 | 0.27 | 0.27 | 0.54 | 0.27 | 0.27 |
| 22. SITS24 | 1.07 | 0.80 | 0.67 | 0.67 | 0.40 | 0.40 | 0.67 | 0.54 | 0.67 | 0.67 | 0.40 | 0.94 | 0.67 | 0.40 |
| 23. SITS25 | 1.07 | 0.54 | 0.67 | 0.67 | 0.94 | 0.94 | 1.21 | 1.07 | 0.94 | 0.67 | 0.94 | 0.94 | 0.67 | 0.94 |
| 24. SITS26 | 1.07 | 0.80 | 0.67 | 0.67 | 0.94 | 0.94 | 1.21 | 1.07 | 0.94 | 0.67 | 0.94 | 0.94 | 0.67 | 0.94 |
| 25. SITS28 | 0.80 | 0.54 | 0.40 | 0.40 | 0.67 | 0.67 | 0.94 | 0.80 | 0.67 | 0.40 | 0.67 | 0.67 | 0.40 | 0.67 |
| 26. SITS29 | 0.80 | 0.54 | 0.40 | 0.40 | 0.67 | 0.67 | 0.94 | 0.80 | 0.67 | 0.40 | 0.67 | 0.67 | 0.40 | 0.67 |
| 27. SITS30 | 0.67 | 0.40 | 0.27 | 0.27 | 0.54 | 0.54 | 0.80 | 0.67 | 0.54 | 0.27 | 0.54 | 0.54 | 0.27 | 0.54 |
| 28. SITS31 | 0.67 | 0.40 | 0.27 | 0.27 | 0.54 | 0.54 | 0.80 | 0.67 | 0.54 | 0.27 | 0.54 | 0.54 | 0.27 | 0.54 |
| 29. SITS32 | 0.80 | 0.54 | 0.40 | 0.40 | 0.67 | 0.67 | 0.94 | 0.80 | 0.67 | 0.40 | 0.67 | 0.67 | 0.40 | 0.67 |
| 30. SITS33 | 1.34 | 1.07 | 0.94 | 0.94 | 1.21 | 1.21 | 1.48 | 1.34 | 1.21 | 0.94 | 1.21 | 1.21 | 0.94 | 1.21 |
| 31. SITS34 | 0.94 | 0.67 | 0.54 | 0.54 | 0.80 | 0.80 | 1.07 | 0.94 | 0.80 | 0.54 | 0.80 | 0.80 | 0.54 | 0.80 |
| 32. SITS35 | 0.54 | 0.54 | 0.40 | 0.40 | 0.67 | 0.67 | 0.94 | 0.80 | 0.67 | 0.40 | 0.67 | 0.67 | 0.40 | 0.67 |
| 33. SITS36 | 0.67 | 0.40 | 0.27 | 0.27 | 0.54 | 0.54 | 0.80 | 0.67 | 0.54 | 0.27 | 0.54 | 0.54 | 0.27 | 0.54 |
| 34. SITS37 | 0.40 | 0.40 | 0.27 | 0.27 | 0.54 | 0.54 | 0.80 | 0.67 | 0.54 | 0.27 | 0.54 | 0.54 | 0.27 | 0.54 |
| 35. SITS38 | 0.67 | 0.40 | 0.27 | 0.27 | 0.54 | 0.54 | 0.80 | 0.67 | 0.54 | 0.27 | 0.54 | 0.54 | 0.27 | 0.54 |
| 36. SITS39 | 0.67 | 0.40 | 0.27 | 0.27 | 0.54 | 0.54 | 0.80 | 0.67 | 0.54 | 0.27 | 0.54 | 0.54 | 0.27 | 0.54 |
| 37. SITS40 | 0.67 | 0.67 | 0.54 | 0.54 | 0.80 | 0.80 | 1.07 | 0.94 | 0.80 | 0.54 | 0.80 | 0.80 | 0.54 | 0.80 |
| 38. SITS41 | 0.67 | 0.40 | 0.27 | 0.27 | 0.54 | 0.54 | 0.80 | 0.67 | 0.54 | 0.27 | 0.54 | 0.54 | 0.27 | 0.54 |
| 39. SITS42 | 0.54 | 0.54 | 0.40 | 0.40 | 0.67 | 0.67 | 0.94 | 0.80 | 0.67 | 0.40 | 0.67 | 0.67 | 0.40 | 0.67 |
| 40. SITS43 | 0.54 | 0.54 | 0.40 | 0.40 | 0.67 | 0.67 | 0.94 | 0.80 | 0.67 | 0.40 | 0.67 | 0.67 | 0.40 | 0.67 |
| 41. SITS44 | 0.67 | 0.40 | 0.27 | 0.27 | 0.54 | 0.54 | 0.80 | 0.67 | 0.54 | 0.27 | 0.54 | 0.54 | 0.27 | 0.54 |
| 42. SITS45 | 0.67 | 0.40 | 0.27 | 0.27 | 0.54 | 0.54 | 0.80 | 0.67 | 0.54 | 0.27 | 0.54 | 0.54 | 0.27 | 0.54 |
| 43. SITS46 | - | 0.80 | 0.67 | 0.67 | 0.94 | 0.94 | 1.21 | 1.07 | 0.94 | 0.67 | 0.94 | 0.94 | 0.67 | 0.94 |
| 44. SITS47 | 6 | - | 0.40 | 0.40 | 0.67 | 0.67 | 0.94 | 0.80 | 0.67 | 0.40 | 0.67 | 0.67 | 0.40 | 0.67 |
| 45. SITS48 | 5 | 3 | - | 0.27 | 0.54 | 0.54 | 0.80 | 0.67 | 0.54 | 0.27 | 0.54 | 0.54 | 0.27 | 0.54 |
| 46. SITS49 | 5 | 3 | 2 | - | 0.54 | 0.54 | 0.80 | 0.67 | 0.54 | 0.27 | 0.54 | 0.54 | 0.27 | 0.54 |
| 47. SITS50 | 7 | 5 | 4 | 4 | - | 0.27 | 0.54 | 0.40 | 0.54 | 0.54 | 0.27 | 0.80 | 0.54 | 0.27 |
| 48. SITS51 | 7 | 5 | 4 | 4 | 2 | - | 0.54 | 0.40 | 0.54 | 0.54 | 0.27 | 0.80 | 0.54 | 0.27 |
| 49. SITS52 | 9 | 7 | 6 | 6 | 4 | 4 | - | 0.67 | 0.80 | 0.80 | 0.54 | 1.07 | 0.80 | 0.54 |
| 50. SITS53 | 8 | 6 | 5 | 5 | 3 | 3 | 5 | - | 0.67 | 0.67 | 0.40 | 0.94 | 0.67 | 0.40 |
| 51. SITS55 | 7 | 5 | 4 | 4 | 4 | 4 | 6 | 5 | - | 0.54 | 0.54 | 0.80 | 0.54 | 0.54 |
| 52. SITS56 | 5 | 3 | 2 | 2 | 4 | 4 | 6 | 5 | 4 | - | 0.27 | 0.54 | 0.27 | 0.54 |
| 53. SITS57 | 7 | 5 | 4 | 4 | 2 | 2 | 4 | 3 | 4 | 2 | - | 0.80 | 0.54 | 0.27 |
| 54. SITS58 | 7 | 5 | 4 | 4 | 6 | 6 | 8 | 7 | 6 | 4 | 6 | - | 0.54 | 0.80 |
| 55. SITS59 | 5 | 3 | 2 | 2 | 4 | 4 | 6 | 5 | 4 | 2 | 4 | 4 | - | 0.54 |
| 56. SITS60 | 7 | 5 | 4 | 4 | 2 | 2 | 4 | 3 | 4 | 4 | 2 | 6 | 4 | - |

Numbers above the diagonal are percent distance values; numbers below the diagonal are absolute distance values.
