# Supplementary material for: Identification of Sympetrum depressiusculum Sélys, 1841 in South Korea (Odonata: Libellulidae) According to Morphology and Genetic Markers
Source: Insects. 2023 Aug 30;14(9):733. doi: 10.3390/insects14090733 (PMC10531817; doi:10.3390/insects14090733)
Supplement: Supplementary file 1 [file insects-14-00733-s001.zip › Table S2. Primer list.docx]

**Table S2.** Primers utilized to amplify and sequence *COI*, *16S rRNA*, and the ITS region.

| Region | Length (bp)* | Name | Sequence (5' - 3') | References |
| --- | --- | --- | --- | --- |
| *COI* | 451/497 | COI-S1n | ATA ATT GGR GGR TTY GGA AAC TG | Futahashi et al. [24] |
|  |  | COI-AS1 | CCA AAR AAT CAA AAT AAR TGT TG | Hayashi et al. [25] |
| *16S rRNA* | 509/552 | 16S-F0^†^ | TTA AAC CGG TYT GAA CTC AGA TC | Futahashi et al. [24] |
|  |  | 16S-R3^†^ | CGC CTG TTT ATC AAA AAC AT | Okude et al. [26] |
|  | 276/322 | 16S-F3^‡^ | ACG CTG TTA TCC CTA AGG TAA C | Sugimura et al. [27] |
|  |  | 16S-R3^‡^ | GCA AAG GTA GCA TAA TCA TTA GTC | Okude et al. [26] |
| ITS** | 805/849 | ITS-F | TAG AGG AAG TAA AAG TCG | Weekers et al. [28] |
|  |  | 28S-AS1 | TTT CAC TCG CCG TTA CTA AGG GAA TC | Futahashi and Sasamoto [29] |

^†^Used for PCR amplification. ^‡^Used for sequencing.

*PCR-product size excluding primer sites (first number)/including primer sites (second number).

**The ITS region is composed of 40 bp of *18S rRNA*, 261 bp of ITS1, 148 bp of *5.8S rRNA*, 312 bp of ITS2, and 44 bp of *28S rRNA*, excluding primer sites.
