# Supplementary material for: Identification of Sympetrum depressiusculum Sélys, 1841 in South Korea (Odonata: Libellulidae) According to Morphology and Genetic Markers
Source: Insects. 2023 Aug 30;14(9):733. doi: 10.3390/insects14090733 (PMC10531817; doi:10.3390/insects14090733)
Supplement: Supplementary file 1 [file insects-14-00733-s001.zip › Table S3. Sample list-Ours+GB.docx]

**Table S3.** List of *Sympetrum* species samples sequenced in this study and obtained from public data.

| Locality  (no. of individuals) | | Sample number | GenBank no. | *COI*  (451 bp) | *16S rRNA*  (276 bp) | *COI* +  *16S rRNA*  (727 bp) | ITS  (746 bp) |
| --- | --- | --- | --- | --- | --- | --- | --- |
| 1. Inje, South Korea (10) | | CNU15186 | OR028963 / OR029037 / OR029122 | SCOI10 | S16S01 | SMT20 | SITS01 |
|  |  | CNU15187 | OR028964 / OR029038 / - | SCOI11 | S16S06 | SMT21 | - |
|  |  | CNU15187-1 | - / - / OR029123 | - | - | - | SITS04 |
|  |  | CNU15187-2 | - / - / OR029124 | - | - | - | SITS05 |
|  |  | CNU15187-3 | - / - / OR029125 | - | - | - | SITS06 |
|  |  | CNU15187-4 | - / - / OR029126 | - | - | - | SITS07 |
|  |  | CNU15187-5 | - / - / OR029127 | - | - | - | SITS05 |
|  |  | CNU15188 | OR028965 / OR029039 / OR029128 | SCOI11 | S16S01 | SMT12 | SITS01 |
|  |  | CNU15189 | OR028966 / OR029040 / OR029129 | SCOI12 | S16S04 | SMT22 | SITS01 |
|  |  | CNU15190 | OR028967 / OR029041 / OR029130 | SCOI11 | S16S01 | SMT12 | SITS01 |
|  |  | CNU15191 | OR028968 / OR029042 / OR029131 | SCOI11 | S16S01 | SMT12 | SITS01 |
|  |  | CNU15192 | OR028969 / OR029043 / - | SCOI13 | S16S04 | SMT23 | - |
|  |  | CNU15192-1 | - / - / OR029132 | - | - | - | SITS01 |
|  |  | CNU15192-2 | - / - / OR029133 | - | - | - | SITS01 |
|  |  | CNU15192-3 | - / - / OR029134 | - | - | - | SITS01 |
|  |  | CNU15192-4 | - / - / OR029135 | - | - | - | SITS01 |
|  |  | CNU15192-5 | - / - / OR029136 | - | - | - | SITS08 |
|  |  | CNU15193 | OR028970 / OR029044 / OR029137 | SCOI14 | S16S01 | SMT24 | SITS01 |
|  |  | CNU15194 | OR028971 / OR029045 / OR029138 | SCOI05 | S16S01 | SMT19 | SITS01 |
|  |  | CNU15195 | OR028972 / OR029046 / OR029139 | SCOI15 | S16S01 | SMT25 | SITS01 |
| 2. Paju, South Korea (10) | | CNU15176 | OR028973 / OR029047 / OR029140 | SCOI16 | S16S01 | SMT10 | SITS01 |
|  |  | CNU15177 | OR028974 / OR029048 / - | SCOI17 | S16S01 | SMT11 | - |
|  |  | CNU15177-1 | - / - / OR029141 | - | - | - | SITS09 |
|  |  | CNU15177-2 | - / - / OR029142 | - | - | - | SITS01 |
|  |  | CNU15177-3 | - / - / OR029143 | - | - | - | SITS01 |
|  |  | CNU15177-4 | - / - / OR029144 | - | - | - | SITS10 |
|  |  | CNU15177-5 | - / - / OR029145 | - | - | - | SITS11 |
|  |  | CNU15178 | OR028975 / OR029049 / OR029146 | SCOI11 | S16S01 | SMT12 | SITS01-12 |
|  |  | CNU15179 | OR028976 / OR029050 / OR029147 | SCOI18 | S16S01 | SMT13 | SITS01 |
|  |  | CNU15180 | OR028977 / OR029051 / OR029148 | SCOI19 | S16S01 | SMT14 | SITS01 |
|  |  | CNU15181 | OR028978 / OR029052 / OR029149 | SCOI20 | S16S07 | SMT15 | SITS01 |
|  |  | CNU15182 | OR028979 / OR029053 / OR029150 | SCOI21 | S16S01 | SMT16 | SITS01 |
|  |  | CNU15183 | OR028980 / OR029054 / OR029151 | SCOI22 | S16S01 | SMT17 | SITS13 |
|  |  | CNU15184 | OR028981 / OR029055 / OR029152 | SCOI23 | S16S08 | SMT18 | SITS01 |
|  |  | CNU15185 | OR028982 / OR029056 / OR029153 | SCOI05 | S16S01 | SMT19 | SITS01 |
| 3. Incheon, South Korea (4) | | CNU15338 | OR029010 / OR029084 / OR029217 | SCOI33 | S16S01 | SMT37 | SITS01 |
|  |  | CNU15339 | OR029011 / OR029085 / OR029218 | SCOI34 | S16S01 | SMT38 | SITS50 |
|  |  | CNU15340 | OR029012 / OR029086 / - | SCOI35 | S16S01 | SMT39 | - |
|  |  | CNU15340-1 | - / - / OR029219 | - | - | - | SITS01 |
|  |  | CNU15340-2 | - / - / OR029220 | - | - | - | SITS51 |
|  |  | CNU15340-3 | - / - / OR029221 | - | - | - | SITS01 |
|  |  | CNU15340-4 | - / - / OR029222 | - | - | - | SITS52 |
|  |  | CNU15340-5 | - / - / OR029223 | - | - | - | SITS01 |
|  |  | CNU15341 | OR029013 / OR029087 / - | SCOI36 | S16S01 | SMT40 | - |
|  |  | CNU15341-1 | - / - / OR029224 | - | - | - | SITS53 |
|  |  | CNU15341-2 | - / - / OR029225 | - | - | - | SITS53 |
|  |  | CNU15341-3 | - / - / OR029226 | - | - | - | SITS01-54 |
|  |  | CNU15341-4 | - / - / OR029227 | - | - | - | SITS53 |
|  |  | CNU15341-5 | - / - / OR029228 | - | - | - | SITS01 |
| 4. Boeun, South Korea (10) | | CNU15196 | OR028983 / OR029057 / OR029154 | SCOI05 | S16S09 | SMT26 | SITS01 |
|  |  | CNU15197 | OR028984 / OR029058 / OR029155 | SCOI24 | S16S01 | SMT27 | SITS14 |
|  |  | CNU15198 | OR028985 / OR029059 / - | SCOI25 | S16S10 | SMT28 | - |
|  |  | CNU15198-1 | - / - / OR029156 | - | - | - | SITS01 |
|  |  | CNU15198-2 | - / - / OR029157 | - | - | - | SITS15 |
|  |  | CNU15198-3 | - / - / OR029158 | - | - | - | SITS16 |
|  |  | CNU15198-4 | - / - / OR029159 | - | - | - | SITS17 |
|  |  | CNU15198-5 | - / - / OR029160 | - | - | - | SITS18 |
|  |  | CNU15199 | OR028986 / OR029060 / - | SCOI11 | S16S01 | SMT12 | - |
|  |  | CNU15199-1 | - / - / OR029161 | - | - | - | SITS19 |
|  |  | CNU15199-2 | - / - / OR029162 | - | - | - | SITS20 |
|  |  | CNU15199-3 | - / - / OR029163 | - | - | - | SITS20 |
|  |  | CNU15199-4 | - / - / OR029164 | - | - | - | SITS19 |
|  |  | CNU15199-5 | - / - / OR029165 | - | - | - | SITS21 |
|  |  | CNU15200 | OR028987 / OR029061 / OR029166 | SCOI26 | S16S07 | SMT29 | SITS01 |
|  |  | CNU15201 | OR028988 / OR029062 / OR029167 | SCOI11 | S16S01 | SMT12 | SITS01 |
|  |  | CNU15202 | OR028989 / OR029063 / OR029168 | SCOI27 | S16S01 | SMT30 | SITS01 |
|  |  | CNU15203 | OR028990 / OR029064 / OR029169 | SCOI28 | S16S01 | SMT31 | SITS01 |
|  |  | CNU15204 | OR028991 / OR029065 / OR029170 | SCOI29 | S16S01 | SMT32 | SITS01 |
|  |  | CNU15205 | OR028992 / OR029066 / - | SCOI30 | S16S01 | SMT33 | - |
|  |  | CNU15205-1 | - / - / OR029171 | - | - | - | SITS22 |
|  |  | CNU15205-2 | - / - / OR029172 | - | - | - | SITS23 |
|  |  | CNU15205-3 | - / - / OR029173 | - | - | - | SITS24 |
|  |  | CNU15205-4 | - / - / OR029174 | - | - | - | SITS24 |
|  |  | CNU15205-5 | - / - / OR029175 | - | - | - | SITS25 |
| 5. Jeongseon, South Korea (13) | | CNU15718 | OR029014 / OR029088 / - | SCOI11 | S16S01 | SMT12 | - |
|  |  | CNU15718-1 | - / - / OR029229 | - | - | - | SITS55 |
|  |  | CNU15718-2 | - / - / OR029230 | - | - | - | SITS56 |
|  |  | CNU15718-3 | - / - / OR029231 | - | - | - | SITS56 |
|  |  | CNU15718-4 | - / - / OR029232 | - | - | - | SITS56 |
|  |  | CNU15718-5 | - / - / OR029233 | - | - | - | SITS01 |
|  |  | CNU15719 | OR029015 / OR029089 / OR029234 | SCOI37 | S16S01 | SMT41 | SITS01 |
|  |  | CNU15720 | OR029016 / OR029090 / OR029235 | SCOI38 | S16S01 | SMT42 | SITS01 |
|  |  | CNU15721 | OR029017 / OR029091 / OR029236 | SCOI39 | S16S01 | SMT43 | SITS01 |
|  |  | CNU15722 | OR029018 / OR029092 / OR029237 | SCOI34 | S16S01 | SMT38 | SITS01 |
|  |  | CNU15723 | OR029019 / OR029093 / OR029238 | SCOI40 | S16S01 | SMT44 | SITS01 |
|  |  | CNU15724 | OR029020 / OR029094 / OR029239 | SCOI41 | S16S12 | SMT45 | SITS01 |
|  |  | CNU15725 | OR029021 / OR029095 / OR029240 | SCOI42 | S16S13 | SMT46 | SITS01 |
|  |  | CNU15726 | OR029022 / OR029096 / OR029241 | SCOI43 | S16S01 | SMT47 | SITS01 |
|  |  | CNU15727 | OR029023 / OR029097 / OR029242 | SCOI42 | S16S13 | SMT46 | SITS01 |
|  |  | CNU15728 | OR029024 / OR029098 / OR029243 | SCOI44 | S16S01 | SMT48 | SITS01 |
|  |  | CNU15729 | OR029025 / OR029099 / OR029244 | SCOI45 | S16S14 | SMT49 | SITS01 |
|  |  | CNU15730 | OR029026 / OR029100 / OR029245 | SCOI46 | S16S15 | SMT50 | SITS57 |
| 6. Japan (16) | Okayama (10) | CNU15157 | OR028953 / OR029027 / OR029108 | SCOI01 | S16S01 | SMT01 | SITS01 |
|  |  | CNU15158 | OR028954 / OR029028 / OR029109 | SCOI02 | S16S01 | SMT02 | SITS01 |
|  |  | CNU15159 | OR028955 / OR029029 / OR029110 | SCOI03 | S16S02 | SMT03 | SITS01 |
|  |  | CNU15160 | OR028956 / OR029030 / - | SCOI04 | S16S03 | SMT04 | - |
|  |  | CNU15160-1 | - / - / OR029111 | - | - | - | SITS01 |
|  |  | CNU15160-2 | - / - / OR029112 | - | - | - | SITS02 |
|  |  | CNU15160-3 | - / - / OR029113 | - | - | - | SITS01 |
|  |  | CNU15160-4 | - / - / OR029114 | - | - | - | SITS01 |
|  |  | CNU15160-5 | - / - / OR029115 | - | - | - | SITS01 |
|  |  | CNU15161 | OR028957 / OR029031 / OR029116 | SCOI05 | S16S04 | SMT05 | SITS01 |
|  |  | CNU15162 | OR028958 / OR029032 / OR029117 | SCOI06 | S16S05 | SMT06 | SITS01-03 |
|  |  | CNU15163 | OR028959 / OR029033 / OR029118 | SCOI07 | S16S01 | SMT07 | SITS01 |
|  |  | CNU15164 | OR028960 / OR029034 / OR029119 | SCOI07 | S16S01 | SMT07 | SITS01 |
|  |  | CNU15165 | OR028961 / OR029035 / OR029120 | SCOI08 | S16S01 | SMT08 | SITS01 |
|  |  | CNU15166 | OR028962 / OR029036 / OR029121 | SCOI09 | S16S01 | SMT09 | SITS01 |
|  |  |  |  |  |  |  |  |
|  | Nagano (1) | RF661 | AB709158 / AB708214 / AB707264 | SCOI59 | S16S01 | SMT73 | SITS01 |
|  |  |  |  |  |  |  |  |
|  | Hokkaido (2) | RF1831 | AB709144 / AB708200 / AB707250 | SCOI47 | S16S01 | SMT71 | SITS10 |
|  |  | RF2000 | LC366661 / LC366364 / LC366067 | SCOI60 | S16S01 | SMT72 | SITS01 |
|  |  |  |  |  |  |  |  |
|  | Toyama (3) | RF1360 | AB709126 / AB708182 / AB707232 | SCOI48 | S16S01 | SMT52 | SITS10 |
|  |  | RF1527 | AB709127 / AB708183 / AB707233 | SCOI49 | S16S01 | SMT54 | SITS10 |
|  |  | RF1520 | AB709159 / AB708215 / AB707265 | SCOI61 | S16S05 | SMT53 | SITS01 |
| 7. Russia (28) | Novosibirsk region (2) | CNU15309 | OR028993 / OR029067 / OR029176 | SCOI31 | S16S11 | SMT34 | SITS26 |
|  |  | CNU15310 | OR028994 / OR029068 / OR029177 | SCOI31 | S16S01 | SMT35 | SITS26-27 |
|  |  |  |  |  |  |  |  |
|  | Republic of Dagestan (3) | CNU15311 | OR028995 / OR029069 / - | SCOI31 | S16S01 | SMT35 | - |
|  |  | CNU15311-1 | - / - / OR029178 | - | - | - | SITS28 |
|  |  | CNU15311-2 | - / - / OR029179 | - | - | - | SITS29 |
|  |  | CNU15311-3 | - / - / OR029180 | - | - | - | SITS10 |
|  |  | CNU15311-4 | - / - / OR029181 | - | - | - | SITS30 |
|  |  | CNU15311-5 | - / - / OR029182 | - | - | - | SITS10 |
|  |  | CNU15312 | OR028996 / OR029070 / OR029183 | SCOI31 | S16S01 | SMT35 | SITS31 |
|  |  | CNU15313 | OR028997 / OR029071 / OR029184 | SCOI31 | S16S01 | SMT35 | SITS32 |
|  |  |  |  |  |  |  |  |
|  | Unknown (23) | RF1299 | AB709125 / AB708181 / - | SCOI50 | S16S01 | SMT51 | - |
|  |  | RF1772 | AB709128 / AB708184 / AB707234 | SCOI51 | S16S01 | SMT58 | SITS58 |
|  |  | RF1774 | AB709129 / AB708185 / AB707235 | SCOI52 | S16S16 | SMT59 | SITS10 |
|  |  | RF1776 | AB709130 / AB708186 / AB707236 | SCOI28 | S16S17 | SMT61 | SITS10 |
|  |  | RF1779 | AB709131 / AB708187 / AB707237 | SCOI53 | S16S01 | SMT63 | SITS10 |
|  |  | RF1780 | AB709132 / AB708188 / AB707238 | SCOI26 | S16S01 | SMT64 | SITS10 |
|  |  | RF1783 | AB709133 / AB708189 / AB707239 | SCOI30 | S16S01 | SMT33 | SITS10 |
|  |  | RF1787 | AB709134 / AB708190 / - | SCOI53 | S16S01 | SMT63 | - |
|  |  | RF1788 | AB709135 / AB708191 / AB707241 | SCOI54 | S16S18 | SMT65 | SITS10 |
|  |  | RF1789 | AB709136 / AB708192 / - | SCOI05 | S16S01 | SMT19 | - |
|  |  | RF1790 | AB709137 / AB708193 / AB707243 | SCOI55 | S16S01 | SMT66 | SITS10 |
|  |  | RF1791 | AB709138 / AB708194 / AB707244 | SCOI56 | S16S01 | SMT67 | SITS10 |
|  |  | RF1792 | AB709139 / AB708195 / - | SCOI46 | S16S01 | SMT68 | - |
|  |  | RF1793 | AB709140 / AB708196 / AB707246 | SCOI33 | S16S01 | SMT37 | SITS10 |
|  |  | RF1794 | AB709141 / AB708197 / AB707247 | SCOI57 | S16S07 | SMT69 | SITS59 |
|  |  | RF1795 | AB709142 / AB708198 / AB707248 | SCOI11 | S16S01 | SMT12 | SITS10 |
|  |  | RF1796 | AB709143 / AB708199 / AB707249 | SCOI58 | S16S01 | SMT70 | SITS26 |
|  |  | RF1755 | AB709160 / AB708216 / AB707266 | SCOI62 | S16S01 | SMT55 | SITS60 |
|  |  | RF1756 | AB709161 / AB708217 / AB707267 | SCOI63 | S16S01 | SMT56 | SITS01 |
|  |  | RF1760 | AB709162 / AB708218 / AB707268 | SCOI64 | S16S01 | SMT57 | SITS01 |
|  |  | RF1775 | AB709163 / AB708219 / AB707269 | SCOI03 | S16S20 | SMT60 | SITS01 |
|  |  | RF1777 | AB709164 / AB708220 / AB707270 | SCOI65 | S16S06 | SMT62 | SITS01 |
|  |  | RF1786 | AB709165 / AB708221 / AB707271 | SCOI11 | S16S01 | SMT12 | SITS01 |
| 8. The Netherlands (22) | Overijssel (12) | CNU15314 | OR028998 / OR029072 / OR029185 | SCOI32 | S16S01 | SMT36 | SITS10 |
|  |  | CNU15315 | OR028999 / OR029073 / OR029186 | SCOI32 | S16S01 | SMT36 | SITS33 |
|  |  | CNU15316 | OR029000 / OR029074 / OR029187 | SCOI32 | S16S01 | SMT36 | SITS34 |
|  |  | CNU15317 | OR029001 / OR029075 / - | SCOI32 | S16S01 | SMT36 | - |
|  |  | CNU15317-1 | - / - / OR029188 | - | - | - | SITS35 |
|  |  | CNU15317-2 | - / - / OR029189 | - | - | - | SITS36 |
|  |  | CNU15317-3 | - / - / OR029190 | - | - | - | SITS37 |
|  |  | CNU15317-4 | - / - / OR029191 | - | - | - | SITS38 |
|  |  | CNU15317-5 | - / - / OR029192 | - | - | - | SITS10 |
|  |  | CNU15318 | OR029002 / OR029076 / OR029193 | SCOI32 | S16S01 | SMT36 | SITS10 |
|  |  | CNU15319 | OR029003 / OR029077 / - | SCOI32 | S16S01 | SMT36 | - |
|  |  | CNU15319-1 | - / - / OR029194 | - | - | - | SITS37 |
|  |  | CNU15319-2 | - / - / OR029195 | - | - | - | SITS10 |
|  |  | CNU15319-3 | - / - / OR029196 | - | - | - | SITS37 |
|  |  | CNU15319-4 | - / - / OR029197 | - | - | - | SITS39 |
|  |  | CNU15319-5 | - / - / OR029198 | - | - | - | SITS10 |
|  |  | CNU15320 | OR029004 / OR029078 / OR029199 | SCOI32 | S16S01 | SMT36 | SITS40 |
|  |  | CNU15321 | OR029005 / OR029079 / OR029200 | SCOI32 | S16S01 | SMT36 | SITS41 |
|  |  | CNU15322 | OR029006 / OR029080 / - | SCOI32 | S16S01 | SMT36 | - |
|  |  | CNU15322-1 | - / - / OR029201 | - | - | - | SITS42 |
|  |  | CNU15322-2 | - / - / OR029202 | - | - | - | SITS43 |
|  |  | CNU15322-3 | - / - / OR029203 | - | - | - | SITS37 |
|  |  | CNU15322-4 | - / - / OR029204 | - | - | - | SITS37 |
|  |  | CNU15322-5 | - / - / OR029205 | - | - | - | SITS44 |
|  |  | CNU15323 | OR029007 / OR029081 / - | SCOI32 | S16S01 | SMT36 | - |
|  |  | CNU15323-1 | - / - / OR029206 | - | - | - | SITS45 |
|  |  | CNU15323-2 | - / - / OR029207 | - | - | - | SITS45 |
|  |  | CNU15323-3 | - / - / OR029208 | - | - | - | SITS46 |
|  |  | CNU15323-4 | - / - / OR029209 | - | - | - | SITS10 |
|  |  | CNU15323-5 | - / - / OR029210 | - | - | - | SITS47 |
|  |  | CNU15324 | OR029008 / OR029082 / - | SCOI32 | S16S01 | SMT36 | - |
|  |  | CNU15324-1 | - / - / OR029211 | - | - | - | SITS10 |
|  |  | CNU15324-2 | - / - / OR029212 | - | - | - | SITS48 |
|  |  | CNU15324-3 | - / - / OR029213 | - | - | - | SITS49 |
|  |  | CNU15324-4 | - / - / OR029214 | - | - | - | SITS10 |
|  |  | CNU15324-5 | - / - / OR029215 | - | - | - | SITS10 |
|  |  | CNU15325 | OR029009 / OR029083 / OR029216 | SCOI32 | S16S01 | SMT36 | SITS10 |
|  |  |  |  |  |  |  |  |
|  | Unknown (10) | RF3614 | LC365774 / LC365764 / - | SCOI32 | S16S01 | SMT36 | - |
|  |  | RF3615 | LC365775 / LC365765 / LC365755 | SCOI32 | S16S01 | SMT36 | SITS26 |
|  |  | RF3616 | LC365776 / LC365766 / LC365756 | SCOI32 | S16S01 | SMT36 | SITS10 |
|  |  | RF3617 | LC365777 / LC365767 / - | SCOI32 | S16S01 | SMT36 | - |
|  |  | RF3618 | LC365778 / LC365768 / - | SCOI32 | S16S01 | SMT36 | - |
|  |  | RF3619 | LC365779 / LC365769 / - | SCOI32 | S16S01 | SMT36 | - |
|  |  | RF3620 | LC365780 / LC365770 / LC365760 | SCOI32 | S16S01 | SMT36 | SITS10 |
|  |  | RF3621 | LC365781 / LC365771 / LC365761 | SCOI32 | S16S01 | SMT36 | SITS10 |
|  |  | RF3622 | LC365782 / LC365772 / LC365762 | SCOI32 | S16S01 | SMT36 | SITS10 |
|  |  | RF3623 | LC365783 / LC365773 / LC365763 | SCOI32 | S16S01 | SMT36 | SITS37 |

-, not available. Sample names that start with “CNU” were collected in this study. GenBank numbers are presented in the order of *COI*, *16S rRNA*, and the ITS region.
