# Supplementary material for: Identification of Sympetrum depressiusculum Sélys, 1841 in South Korea (Odonata: Libellulidae) According to Morphology and Genetic Markers
Source: Insects. 2023 Aug 30;14(9):733. doi: 10.3390/insects14090733 (PMC10531817; doi:10.3390/insects14090733)
Supplement: Supplementary file 1 [file insects-14-00733-s001.zip › Table S4. HF-Our COI+GB COI.docx]

**Table S4.** Relative frequencies of *COI* haplotypes of *Sympetrum* species sequenced in this study and collected from public data.

| Haplotype | Locality | | | | | | | | Total  (113) |
| --- | --- | --- | --- | --- | --- | --- | --- | --- | --- |
|  | K IJ  (10) | KPJ  (10) | KIC  (4) | KBE  (10) | KJS  (13) | JP  (16) | RU  (28) | ND  (22) |  |
| SCOI01 |  |  |  |  |  | 0.06 (1) |  |  | 0.009 (1) |
| SCOI02 |  |  |  |  |  | 0.06 (1) |  |  | 0.009 (1) |
| SCOI03 |  |  |  |  |  | 0.06 (1) | 0.04 (1) |  | 0.018 (2) |
| SCOI04 |  |  |  |  |  | 0.06 (1) |  |  | 0.009 (1) |
| SCOI05 | 0.10 (1) | 0.10 (1) |  | 0.10 (1) |  | 0.06 (1) | 0.04 (1) |  | 0.044 (5) |
| SCOI06 |  |  |  |  |  | 0.06 (1) |  |  | 0.009 (1) |
| SCOI07 |  |  |  |  |  | 0.13 (2) |  |  | 0.018 (2) |
| SCOI08 |  |  |  |  |  | 0.06 (1) |  |  | 0.009 (1) |
| SCOI09 |  |  |  |  |  | 0.06 (1) |  |  | 0.009 (1) |
| SCOI10 | 0.10 (1) |  |  |  |  |  |  |  | 0.009 (1) |
| SCOI11 | 0.40 (4) | 0.10 (1) |  | 0.20 (2) | 0.08 (1) |  | 0.07 (2) |  | 0.088 (10) |
| SCOI12 | 0.10 (1) |  |  |  |  |  |  |  | 0.009 (1) |
| SCOI13 | 0.10 (1) |  |  |  |  |  |  |  | 0.009 (1) |
| SCOI14 | 0.10 (1) |  |  |  |  |  |  |  | 0.009 (1) |
| SCOI15 | 0.10 (1) |  |  |  |  |  |  |  | 0.009 (1) |
| SCOI16 |  | 0.10 (1) |  |  |  |  |  |  | 0.009 (1) |
| SCOI17 |  | 0.10 (1) |  |  |  |  |  |  | 0.009 (1) |
| SCOI18 |  | 0.10 (1) |  |  |  |  |  |  | 0.009 (1) |
| SCOI19 |  | 0.10 (1) |  |  |  |  |  |  | 0.009 (1) |
| SCOI20 |  | 0.10 (1) |  |  |  |  |  |  | 0.009 (1) |
| SCOI21 |  | 0.10 (1) |  |  |  |  |  |  | 0.009 (1) |
| SCOI22 |  | 0.10 (1) |  |  |  |  |  |  | 0.009 (1) |
| SCOI23 |  | 0.10 (1) |  |  |  |  |  |  | 0.009 (1) |
| SCOI24 |  |  |  | 0.10 (1) |  |  |  |  | 0.009 (1) |
| SCOI25 |  |  |  | 0.10 (1) |  |  |  |  | 0.009 (1) |
| SCOI26 |  |  |  | 0.10 (1) |  |  | 0.04 (1) |  | 0.018 (2) |
| SCOI27 |  |  |  | 0.10 (1) |  |  |  |  | 0.009 (1) |
| SCOI28 |  |  |  | 0.10 (1) |  |  | 0.04 (1) |  | 0.018 (2) |
| SCOI29 |  |  |  | 0.10 (1) |  |  |  |  | 0.009 (1) |
| SCOI30 |  |  |  | 0.10 (1) |  |  | 0.04 (1) |  | 0.018 (2) |
| SCOI31 |  |  |  |  |  |  | 0.18 (5) |  | 0.044 (5) |
| SCOI32 |  |  |  |  |  |  |  | 1.00 (22) | 0.195 (22) |
| SCOI33 |  |  | 0.25 (1) |  |  |  | 0.04 (1) |  | 0.018 (2) |
| SCOI34 |  |  | 0.25 (1) |  | 0.08 (1) |  |  |  | 0.018 (2) |
| SCOI35 |  |  | 0.25 (1) |  |  |  |  |  | 0.009 (1) |
| SCOI36 |  |  | 0.25 (1) |  |  |  |  |  | 0.009 (1) |
| SCOI37 |  |  |  |  | 0.08 (1) |  |  |  | 0.009 (1) |
| SCOI38 |  |  |  |  | 0.08 (1) |  |  |  | 0.009 (1) |
| SCOI39 |  |  |  |  | 0.08 (1) |  |  |  | 0.009 (1) |
| SCOI40 |  |  |  |  | 0.08 (1) |  |  |  | 0.009 (1) |
| SCOI41 |  |  |  |  | 0.08 (1) |  |  |  | 0.009 (1) |
| SCOI42 |  |  |  |  | 0.15 (2) |  |  |  | 0.018 (2) |
| SCOI43 |  |  |  |  | 0.08 (1) |  |  |  | 0.009 (1) |
| SCOI44 |  |  |  |  | 0.08 (1) |  |  |  | 0.009 (1) |
| SCOI45 |  |  |  |  | 0.08 (1) |  |  |  | 0.009 (1) |
| SCOI46 |  |  |  |  | 0.08 (1) |  | 0.04 (1) |  | 0.018 (2) |
| SCOI47 |  |  |  |  |  | 0.06 (1) |  |  | 0.009 (1) |
| SCOI48 |  |  |  |  |  | 0.06 (1) |  |  | 0.009 (1) |
| SCOI49 |  |  |  |  |  | 0.06 (1) |  |  | 0.009 (1) |
| SCOI50 |  |  |  |  |  |  | 0.04 (1) |  | 0.009 (1) |
| SCOI51 |  |  |  |  |  |  | 0.04 (1) |  | 0.009 (1) |
| SCOI52 |  |  |  |  |  |  | 0.04 (1) |  | 0.009 (1) |
| SCOI53 |  |  |  |  |  |  | 0.07 (2) |  | 0.018 (2) |
| SCOI54 |  |  |  |  |  |  | 0.04 (1) |  | 0.009 (1) |
| SCOI55 |  |  |  |  |  |  | 0.04 (1) |  | 0.009 (1) |
| SCOI56 |  |  |  |  |  |  | 0.04 (1) |  | 0.009 (1) |
| SCOI57 |  |  |  |  |  |  | 0.04 (1) |  | 0.009 (1) |
| SCOI58 |  |  |  |  |  |  | 0.04 (1) |  | 0.009 (1) |
| SCOI59 |  |  |  |  |  | 0.06 (1) |  |  | 0.009 (1) |
| SCOI60 |  |  |  |  |  | 0.06 (1) |  |  | 0.009 (1) |
| SCOI61 |  |  |  |  |  | 0.06 (1) |  |  | 0.009 (1) |
| SCOI62 |  |  |  |  |  |  | 0.04 (1) |  | 0.009 (1) |
| SCOI63 |  |  |  |  |  |  | 0.04 (1) |  | 0.009 (1) |
| SCOI64 |  |  |  |  |  |  | 0.04 (1) |  | 0.009 (1) |
| SCOI65 |  |  |  |  |  |  | 0.04 (1) |  | 0.009 (1) |

Numbers in parentheses indicate the number of individuals. Full locality and country names are as follows: KIJ, South Korean Inje; KPJ, Paju; KBE, Boeun; KIC, Incheon; KJS, Jeongseon; JP, Japan; RU, Russia; and ND, The Netherlands.
