# Supplementary material for: Identification of Sympetrum depressiusculum Sélys, 1841 in South Korea (Odonata: Libellulidae) According to Morphology and Genetic Markers
Source: Insects. 2023 Aug 30;14(9):733. doi: 10.3390/insects14090733 (PMC10531817; doi:10.3390/insects14090733)
Supplement: Supplementary file 1 [file insects-14-00733-s001.zip › Table S5. HF-Our 16S+GB 16S.docx]

**Table S5.** Relative frequencies of *16S rRNA* haplotypes of *Sympetrum* species sequenced in this study and collected from public data.

| Haplotype | Locality | | | | | | | | Total  (113) |
| --- | --- | --- | --- | --- | --- | --- | --- | --- | --- |
|  | KIJ  (10) | KPJ  (10) | KIC  (4) | KBE  (10) | KJS  (13) | JP  (16) | RU  (28) | ND  (22) |  |
| S16S01 | 0.70 (7) | 0.80 (8) | 1.00 (4) | 0.70 (7) | 0.62 (8) | 0.67 (11) | 0.75 (21) | 1.00 (22) | 0.779 (88) |
| S16S02 |  |  |  |  |  | 0.06 (1) |  |  | 0.009 (1) |
| S16S03 |  |  |  |  |  | 0.06 (1) |  |  | 0.009 (1) |
| S16S04 | 0.20 (2) |  |  |  |  | 0.06 (1) |  |  | 0.027 (3) |
| S16S05 |  |  |  |  |  | 0.11 (2) |  |  | 0.018 (2) |
| S16S06 | 0.10 (1) |  |  |  |  |  | 0.04 (1) |  | 0.018 (2) |
| S16S07 |  | 0.10 (1) |  | 0.10 (1) |  |  | 0.04 (1) |  | 0.027 (3) |
| S16S08 |  | 0.10 (1) |  |  |  |  |  |  | 0.009 (1) |
| S16S09 |  |  |  | 0.10 (1) |  |  |  |  | 0.009 (1) |
| S16S10 |  |  |  | 0.10 (1) |  |  |  |  | 0.009 (1) |
| S16S11 |  |  |  |  |  |  | 0.04 (1) |  | 0.009 (1) |
| S16S12 |  |  |  |  | 0.08 (1) |  |  |  | 0.009 (1) |
| S16S13 |  |  |  |  | 0.15 (2) |  |  |  | 0.018 (2) |
| S16S14 |  |  |  |  | 0.08 (1) |  |  |  | 0.009 (1) |
| S16S15 |  |  |  |  | 0.08 (1) |  |  |  | 0.009 (1) |
| S16S16 |  |  |  |  |  |  | 0.04 (1) |  | 0.009 (1) |
| S16S17 |  |  |  |  |  |  | 0.04 (1) |  | 0.009 (1) |
| S16S18 |  |  |  |  |  |  | 0.04 (1) |  | 0.009 (1) |
| S16S20 |  |  |  |  |  |  | 0.04 (1) |  | 0.009 (1) |

Numbers in parentheses indicate the number of individuals. Full locality and country names are as follows: KIJ, South Korean Inje; KPJ, Paju; KBE, Boeun; KIC, Incheon; KJS, Jeongseon; JP, Japan; RU, Russia; and ND, The Netherlands.
