# Supplementary material for: Identification of Sympetrum depressiusculum Sélys, 1841 in South Korea (Odonata: Libellulidae) According to Morphology and Genetic Markers
Source: Insects. 2023 Aug 30;14(9):733. doi: 10.3390/insects14090733 (PMC10531817; doi:10.3390/insects14090733)
Supplement: Supplementary file 1 [file insects-14-00733-s001.zip › Table S6. HF-Our COI+16S+GB COI+16S.docx]

**Table S6.** Relative frequencies of *COI* + *16S rRNA* haplotypes of *Sympetrum* species sequenced in this study and collected from public data.

| Haplotype | Locality | | | | | | | | Total  (113) |
| --- | --- | --- | --- | --- | --- | --- | --- | --- | --- |
|  | K IJ  (10) | KPJ  (10) | KIC  (4) | KBE  (10) | KJS  (13) | JP  (16) | RU  (28) | ND  (22) |  |
| SMT01 |  |  |  |  |  | 0.06 (1) |  |  | 0.009 (1) |
| SMT02 |  |  |  |  |  | 0.06 (1) |  |  | 0.009 (1) |
| SMT03 |  |  |  |  |  | 0.06 (1) |  |  | 0.009 (1) |
| SMT04 |  |  |  |  |  | 0.06 (1) |  |  | 0.009 (1) |
| SMT05 |  |  |  |  |  | 0.06 (1) |  |  | 0.009 (1) |
| SMT06 |  |  |  |  |  | 0.06 (1) |  |  | 0.009 (1) |
| SMT07 |  |  |  |  |  | 0.13 (2) |  |  | 0.018 (2) |
| SMT08 |  |  |  |  |  | 0.06 (1) |  |  | 0.009 (1) |
| SMT09 |  |  |  |  |  | 0.06 (1) |  |  | 0.009 (1) |
| SMT10 |  | 0.10 (1) |  |  |  |  |  |  | 0.009 (1) |
| SMT11 |  | 0.10 (1) |  |  |  |  |  |  | 0.009 (1) |
| SMT12 | 0.30 (3) | 0.10 (1) |  | 0.20 (2) | 0.08 (1) |  | 0.07 (2) |  | 0.080 (9) |
| SMT13 |  | 0.10 (1) |  |  |  |  |  |  | 0.009 (1) |
| SMT14 |  | 0.10 (1) |  |  |  |  |  |  | 0.009 (1) |
| SMT15 |  | 0.10 (1) |  |  |  |  |  |  | 0.009 (1) |
| SMT16 |  | 0.10 (1) |  |  |  |  |  |  | 0.009 (1) |
| SMT17 |  | 0.10 (1) |  |  |  |  |  |  | 0.009 (1) |
| SMT18 |  | 0.10 (1) |  |  |  |  |  |  | 0.009 (1) |
| SMT19 | 0.10 (1) | 0.10 (1) |  |  |  |  | 0.04 (1) |  | 0.027 (3) |
| SMT20 | 0.10 (1) |  |  |  |  |  |  |  | 0.009 (1) |
| SMT21 | 0.10 (1) |  |  |  |  |  |  |  | 0.009 (1) |
| SMT22 | 0.10 (1) |  |  |  |  |  |  |  | 0.009 (1) |
| SMT23 | 0.10 (1) |  |  |  |  |  |  |  | 0.009 (1) |
| SMT24 | 0.10 (1) |  |  |  |  |  |  |  | 0.009 (1) |
| SMT25 | 0.10 (1) |  |  |  |  |  |  |  | 0.009 (1) |
| SMT26 |  |  |  | 0.10 (1) |  |  |  |  | 0.009 (1) |
| SMT27 |  |  |  | 0.10 (1) |  |  |  |  | 0.009 (1) |
| SMT28 |  |  |  | 0.10 (1) |  |  |  |  | 0.009 (1) |
| SMT29 |  |  |  | 0.10 (1) |  |  |  |  | 0.009 (1) |
| SMT30 |  |  |  | 0.10 (1) |  |  |  |  | 0.009 (1) |
| SMT31 |  |  |  | 0.10 (1) |  |  |  |  | 0.009 (1) |
| SMT32 |  |  |  | 0.10 (1) |  |  |  |  | 0.009 (1) |
| SMT33 |  |  |  | 0.10 (1) |  |  | 0.04 (1) |  | 0.018 (2) |
| SMT34 |  |  |  |  |  |  | 0.04 (1) |  | 0.009 (1) |
| SMT35 |  |  |  |  |  |  | 0.14 (4) |  | 0.035 (4) |
| SMT36 |  |  |  |  |  |  |  | 1.00 (22) | 0.195 (22) |
| SMT37 |  |  | 0.25 (1) |  |  |  | 0.04 (1) |  | 0.018 (2) |
| SMT38 |  |  | 0.25 (1) |  | 0.08 (1) |  |  |  | 0.018 (2) |
| SMT39 |  |  | 0.25 (1) |  |  |  |  |  | 0.009 (1) |
| SMT40 |  |  | 0.25 (1) |  |  |  |  |  | 0.009 (1) |
| SMT41 |  |  |  |  | 0.08 (1) |  |  |  | 0.009 (1) |
| SMT42 |  |  |  |  | 0.08 (1) |  |  |  | 0.009 (1) |
| SMT43 |  |  |  |  | 0.08 (1) |  |  |  | 0.009 (1) |
| SMT44 |  |  |  |  | 0.08 (1) |  |  |  | 0.009 (1) |
| SMT45 |  |  |  |  | 0.08 (1) |  |  |  | 0.009 (1) |
| SMT46 |  |  |  |  | 0.15 (2) |  |  |  | 0.018 (2) |
| SMT47 |  |  |  |  | 0.08 (1) |  |  |  | 0.009 (1) |
| SMT48 |  |  |  |  | 0.08 (1) |  |  |  | 0.009 (1) |
| SMT49 |  |  |  |  | 0.08 (1) |  |  |  | 0.009 (1) |
| SMT50 |  |  |  |  | 0.08 (1) |  |  |  | 0.009 (1) |
| SMT51 |  |  |  |  |  |  | 0.04 (1) |  | 0.009 (1) |
| SMT52 |  |  |  |  |  | 0.06 (1) |  |  | 0.009 (1) |
| SMT53 |  |  |  |  |  | 0.06 (1) |  |  | 0.009 (1) |
| SMT54 |  |  |  |  |  | 0.06 (1) |  |  | 0.009 (1) |
| SMT55 |  |  |  |  |  |  | 0.04 (1) |  | 0.009 (1) |
| SMT56 |  |  |  |  |  |  | 0.04 (1) |  | 0.009 (1) |
| SMT57 |  |  |  |  |  |  | 0.04 (1) |  | 0.009 (1) |
| SMT58 |  |  |  |  |  |  | 0.04 (1) |  | 0.009 (1) |
| SMT59 |  |  |  |  |  |  | 0.04 (1) |  | 0.009 (1) |
| SMT60 |  |  |  |  |  |  | 0.04 (1) |  | 0.009 (1) |
| SMT61 |  |  |  |  |  |  | 0.04 (1) |  | 0.009 (1) |
| SMT62 |  |  |  |  |  |  | 0.04 (1) |  | 0.009 (1) |
| SMT63 |  |  |  |  |  |  | 0.07 (2) |  | 0.018 (2) |
| SMT64 |  |  |  |  |  |  | 0.04 (1) |  | 0.009 (1) |
| SMT65 |  |  |  |  |  |  | 0.04 (1) |  | 0.009 (1) |
| SMT66 |  |  |  |  |  |  | 0.04 (1) |  | 0.009 (1) |
| SMT67 |  |  |  |  |  |  | 0.04 (1) |  | 0.009 (1) |
| SMT68 |  |  |  |  |  |  | 0.04 (1) |  | 0.009 (1) |
| SMT69 |  |  |  |  |  |  | 0.04 (1) |  | 0.009 (1) |
| SMT70 |  |  |  |  |  |  | 0.04 (1) |  | 0.009 (1) |
| SMT71 |  |  |  |  |  | 0.06 (1) |  |  | 0.009 (1) |
| SMT72 |  |  |  |  |  | 0.06 (1) |  |  | 0.009 (1) |
| SMT73 |  |  |  |  |  | 0.06 (1) |  |  | 0.009 (1) |

Numbers in parentheses indicate the number of individuals. Full locality and country names are as follows: KIJ, South Korean Inje; KPJ, Paju; KBE, Boeun; KIC, Incheon; KJS, Jeongseon; JP, Japan; RU, Russia; and ND, The Netherlands.
