# Supplementary material for: Identification of Sympetrum depressiusculum Sélys, 1841 in South Korea (Odonata: Libellulidae) According to Morphology and Genetic Markers
Source: Insects. 2023 Aug 30;14(9):733. doi: 10.3390/insects14090733 (PMC10531817; doi:10.3390/insects14090733)
Supplement: Supplementary file 1 [file insects-14-00733-s001.zip › Table S7. HF-Our ITS+GB ITS.docx]

**Table S7.** Relative frequencies of ITS haplotypes of *Sympetrum* species sequenced in this study and collected from public data.

| Haplotype |  | Locality | | | | | | | | Total  (169) |
| --- | --- | --- | --- | --- | --- | --- | --- | --- | --- | --- |
|  | KIJ  (18) | | KPJ  (14) | KIC  (12) | KBE  (22) | KJS  (17) | JP  (20) | RU  (28) | ND  (38) |  |
| SITS01 | 0.67 (12) | | 0.71 (10) | 0.50 (6) | 0.32 (7) | 0.71 (12) | 0.80 (16) | 0.18 (5) |  | 0.402 (68) |
| SITS02 |  | |  |  |  |  | 0.05 (1) |  |  | 0.006 (1) |
| SITS04 | 0.06 (1) | |  |  |  |  |  |  |  | 0.006 (1) |
| SITS05 | 0.11 (2) | |  |  |  |  |  |  |  | 0.012 (2) |
| SITS06 | 0.06 (1) | |  |  |  |  |  |  |  | 0.006 (1) |
| SITS07 | 0.06 (1) | |  |  |  |  |  |  |  | 0.006 (1) |
| SITS08 | 0.06 (1) | |  |  |  |  |  |  |  | 0.006 (1) |
| SITS09 |  | | 0.07 (1) |  |  |  |  |  |  | 0.006 (1) |
| SITS10 |  | | 0.07 (1) |  |  |  | 0.15 (3) | 0.43 (12) | 0.37 (14) | 0.178 (30) |
| SITS11 |  | | 0.07 (1) |  |  |  |  |  |  | 0.006 (1) |
| SITS13 |  | | 0.07 (1) |  |  |  |  |  |  | 0.006 (1) |
| SITS14 |  | |  |  | 0.05 (1) |  |  |  |  | 0.006 (1) |
| SITS15 |  | |  |  | 0.05 (1) |  |  |  |  | 0.006 (1) |
| SITS16 |  | |  |  | 0.05 (1) |  |  |  |  | 0.006 (1) |
| SITS17 |  | |  |  | 0.05 (1) |  |  |  |  | 0.006 (1) |
| SITS18 |  | |  |  | 0.05 (1) |  |  |  |  | 0.006 (1) |
| SITS19 |  | |  |  | 0.09 (2) |  |  |  |  | 0.012 (2) |
| SITS20 |  | |  |  | 0.09 (2) |  |  |  |  | 0.012 (2) |
| SITS21 |  | |  |  | 0.05 (1) |  |  |  |  | 0.006 (1) |
| SITS22 |  | |  |  | 0.05 (1) |  |  |  |  | 0.006 (1) |
| SITS23 |  | |  |  | 0.05 (1) |  |  |  |  | 0.006 (1) |
| SITS24 |  | |  |  | 0.09 (2) |  |  |  |  | 0.012 (2) |
| SITS25 |  | |  |  | 0.05 (1) |  |  |  |  | 0.006 (1) |
| SITS26 |  | |  |  |  |  |  | 0.11 (3) | 0.03 (1) | 0.024 (4) |
| SITS28 |  | |  |  |  |  |  | 0.04 (1) |  | 0.006 (1) |
| SITS29 |  | |  |  |  |  |  | 0.04 (1) |  | 0.006 (1) |
| SITS30 |  | |  |  |  |  |  | 0.04 (1) |  | 0.006 (1) |
| SITS31 |  | |  |  |  |  |  | 0.04 (1) |  | 0.006 (1) |
| SITS32 |  | |  |  |  |  |  | 0.04 (1) |  | 0.006 (1) |
| SITS33 |  | |  |  |  |  |  |  | 0.03 (1) | 0.006 (1) |
| SITS34 |  | |  |  |  |  |  |  | 0.03 (1) | 0.006 (1) |
| SITS35 |  | |  |  |  |  |  |  | 0.03 (1) | 0.006 (1) |
| SITS36 |  | |  |  |  |  |  |  | 0.03 (1) | 0.006 (1) |
| SITS37 |  | |  |  |  |  |  |  | 0.16 (6) | 0.036 (6) |
| SITS38 |  | |  |  |  |  |  |  | 0.03 (1) | 0.006 (1) |
| SITS39 |  | |  |  |  |  |  |  | 0.03 (1) | 0.006 (1) |
| SITS40 |  | |  |  |  |  |  |  | 0.03 (1) | 0.006 (1) |
| SITS41 |  | |  |  |  |  |  |  | 0.03 (1) | 0.006 (1) |
| SITS42 |  | |  |  |  |  |  |  | 0.03 (1) | 0.006 (1) |
| SITS43 |  | |  |  |  |  |  |  | 0.03 (1) | 0.006 (1) |
| SITS44 |  | |  |  |  |  |  |  | 0.03 (1) | 0.006 (1) |
| SITS45 |  | |  |  |  |  |  |  | 0.05 (2) | 0.012 (2) |
| SITS46 |  | |  |  |  |  |  |  | 0.03 (1) | 0.006 (1) |
| SITS47 |  | |  |  |  |  |  |  | 0.03 (1) | 0.006 (1) |
| SITS48 |  | |  |  |  |  |  |  | 0.03 (1) | 0.006 (1) |
| SITS49 |  | |  |  |  |  |  |  | 0.03 (1) | 0.006 (1) |
| SITS50 |  | |  | 0.08 (1) |  |  |  |  |  | 0.006 (1) |
| SITS51 |  | |  | 0.08 (1) |  |  |  |  |  | 0.006 (1) |
| SITS52 |  | |  | 0.08 (1) |  |  |  |  |  | 0.006 (1) |
| SITS53 |  | |  | 0.25 (3) |  |  |  |  |  | 0.018 (3) |
| SITS55 |  | |  |  |  | 0.06 (1) |  |  |  | 0.006 (1) |
| SITS56 |  | |  |  |  | 0.18 (3) |  |  |  | 0.018 (3) |
| SITS57 |  | |  |  |  | 0.06 (1) |  |  |  | 0.006 (1) |
| SITS58 |  | |  |  |  |  |  | 0.04 (1) |  | 0.006 (1) |
| SITS59 |  | |  |  |  |  |  | 0.04 (1) |  | 0.006 (1) |
| SITS60 |  | |  |  |  |  |  | 0.04 (1) |  | 0.006 (1) |

Numbers in parentheses indicate the number of individuals or clones sequenced. Full locality and country names are as follows: KIJ, South Korean Inje; KPJ, Paju; KBE, Boeun; KIC, Incheon; KJS, Jeongseon; JP, Japan; RU, Russia; and ND, The Netherlands.
