# Supplementary material for: Identification of Sympetrum depressiusculum Sélys, 1841 in South Korea (Odonata: Libellulidae) According to Morphology and Genetic Markers
Source: Insects. 2023 Aug 30;14(9):733. doi: 10.3390/insects14090733 (PMC10531817; doi:10.3390/insects14090733)
Supplement: Supplementary file 1 [file insects-14-00733-s001.zip › Table S8. PC-Our COI+GB COI.docx]

**Table S8.** Pairwise comparisons of *COI* haplotypes of *Sympetrum* species sequenced in this study and collected from public data.

| Haplotype | 1 | 2 | 3 | 4 | 5 | 6 | 7 | 8 | 9 | 10 | 11 | 12 | 13 | 14 | 15 | 16 |
| --- | --- | --- | --- | --- | --- | --- | --- | --- | --- | --- | --- | --- | --- | --- | --- | --- |
| 1. SCOI01 | - | 0.67 | 0.67 | 0.44 | 0.44 | 0.67 | 1.11 | 1.33 | 1.33 | 1.11 | 0.22 | 0.67 | 0.89 | 0.67 | 1.11 | 1.11 |
| 2. SCOI02 | 3 | - | 0.44 | 0.67 | 0.22 | 0.44 | 1.33 | 1.11 | 1.11 | 0.44 | 0.44 | 0.89 | 1.11 | 0.44 | 0.89 | 0.89 |
| 3. SCOI03 | 3 | 2 | - | 0.67 | 0.22 | 0.44 | 1.33 | 1.55 | 1.55 | 0.89 | 0.44 | 0.89 | 1.11 | 0.44 | 1.33 | 0.89 |
| 4. SCOI04 | 2 | 3 | 3 | - | 0.44 | 0.67 | 1.11 | 1.33 | 1.33 | 1.11 | 0.22 | 0.67 | 0.89 | 0.67 | 1.11 | 1.11 |
| 5. SCOI05 | 2 | 1 | 1 | 2 | - | 0.22 | 1.11 | 1.33 | 1.33 | 0.67 | 0.22 | 0.67 | 0.89 | 0.22 | 1.11 | 0.67 |
| 6. SCOI06 | 3 | 2 | 2 | 3 | 1 | - | 1.33 | 1.55 | 1.55 | 0.89 | 0.44 | 0.89 | 1.11 | 0.44 | 1.33 | 0.89 |
| 7. SCOI07 | 5 | 6 | 6 | 5 | 5 | 6 | - | 2.00 | 2.00 | 1.77 | 0.89 | 1.33 | 1.11 | 1.33 | 1.33 | 1.77 |
| 8. SCOI08 | 6 | 5 | 7 | 6 | 6 | 7 | 9 | - | 1.77 | 1.55 | 1.11 | 1.55 | 1.77 | 1.55 | 1.55 | 2.00 |
| 9. SCOI09 | 6 | 5 | 7 | 6 | 6 | 7 | 9 | 8 | - | 1.55 | 1.11 | 1.11 | 1.33 | 1.55 | 1.55 | 2.00 |
| 10. SCOI10 | 5 | 2 | 4 | 5 | 3 | 4 | 8 | 7 | 7 | - | 0.89 | 1.33 | 1.55 | 0.89 | 1.33 | 1.33 |
| 11. SCOI11 | 1 | 2 | 2 | 1 | 1 | 2 | 4 | 5 | 5 | 4 | - | 0.44 | 0.67 | 0.44 | 0.89 | 0.89 |
| 12. SCOI12 | 3 | 4 | 4 | 3 | 3 | 4 | 6 | 7 | 5 | 6 | 2 | - | 0.67 | 0.89 | 1.33 | 1.33 |
| 13. SCOI13 | 4 | 5 | 5 | 4 | 4 | 5 | 5 | 8 | 6 | 7 | 3 | 3 | - | 1.11 | 1.11 | 1.55 |
| 14. SCOI14 | 3 | 2 | 2 | 3 | 1 | 2 | 6 | 7 | 7 | 4 | 2 | 4 | 5 | - | 1.33 | 0.89 |
| 15. SCOI15 | 5 | 4 | 6 | 5 | 5 | 6 | 6 | 7 | 7 | 6 | 4 | 6 | 5 | 6 | - | 1.33 |
| 16. SCOI16 | 5 | 4 | 4 | 5 | 3 | 4 | 8 | 9 | 9 | 6 | 4 | 6 | 7 | 4 | 6 | - |
| 17. SCOI17 | 4 | 3 | 5 | 4 | 4 | 5 | 7 | 6 | 6 | 4 | 3 | 5 | 6 | 5 | 5 | 7 |
| 18. SCOI18 | 2 | 3 | 3 | 2 | 2 | 3 | 5 | 6 | 6 | 5 | 1 | 3 | 4 | 3 | 5 | 5 |
| 19. SCOI19 | 2 | 3 | 3 | 2 | 2 | 3 | 5 | 6 | 6 | 5 | 1 | 3 | 4 | 3 | 5 | 5 |
| 20. SCOI20 | 2 | 3 | 3 | 2 | 2 | 3 | 5 | 6 | 6 | 5 | 1 | 3 | 4 | 3 | 5 | 5 |
| 21. SCOI21 | 3 | 4 | 4 | 3 | 3 | 4 | 4 | 7 | 7 | 6 | 2 | 4 | 5 | 4 | 6 | 6 |
| 22. SCOI22 | 2 | 3 | 3 | 2 | 2 | 3 | 5 | 6 | 6 | 5 | 1 | 3 | 4 | 3 | 5 | 5 |
| 23. SCOI23 | 3 | 4 | 2 | 3 | 3 | 4 | 6 | 7 | 7 | 6 | 2 | 4 | 5 | 4 | 6 | 6 |
| 24. SCOI24 | 4 | 5 | 5 | 4 | 4 | 5 | 5 | 8 | 8 | 7 | 3 | 5 | 6 | 3 | 7 | 7 |
| 25. SCOI25 | 4 | 3 | 3 | 4 | 2 | 3 | 5 | 8 | 8 | 5 | 3 | 5 | 6 | 3 | 7 | 5 |
| 26. SCOI26 | 2 | 3 | 3 | 2 | 2 | 3 | 3 | 6 | 6 | 5 | 1 | 3 | 4 | 3 | 5 | 5 |
| 27. SCOI27 | 2 | 3 | 3 | 2 | 2 | 3 | 5 | 6 | 6 | 5 | 1 | 3 | 4 | 3 | 5 | 5 |
| 28. SCOI28 | 3 | 2 | 4 | 3 | 3 | 4 | 6 | 5 | 5 | 3 | 2 | 4 | 5 | 4 | 4 | 6 |
| 29. SCOI29 | 4 | 5 | 5 | 4 | 4 | 5 | 7 | 6 | 8 | 7 | 3 | 5 | 6 | 5 | 5 | 5 |
| 30. SCOI30 | 2 | 3 | 3 | 2 | 2 | 3 | 3 | 6 | 6 | 5 | 1 | 3 | 2 | 3 | 3 | 5 |
| 31. SCOI31 | 3 | 4 | 4 | 3 | 3 | 4 | 6 | 7 | 7 | 5 | 2 | 4 | 5 | 4 | 6 | 6 |
| 32. SCOI32 | 2 | 3 | 3 | 2 | 2 | 3 | 5 | 6 | 6 | 4 | 1 | 3 | 4 | 3 | 5 | 5 |
| 33. SCOI33 | 4 | 5 | 5 | 4 | 4 | 4 | 7 | 8 | 8 | 7 | 3 | 5 | 6 | 5 | 7 | 7 |
| 34. SCOI34 | 2 | 3 | 3 | 2 | 2 | 3 | 5 | 6 | 6 | 5 | 1 | 3 | 4 | 3 | 5 | 5 |
| 35. SCOI35 | 3 | 2 | 4 | 3 | 3 | 4 | 6 | 3 | 5 | 4 | 2 | 4 | 5 | 4 | 4 | 6 |
| 36. SCOI36 | 3 | 4 | 4 | 3 | 3 | 4 | 6 | 7 | 7 | 6 | 2 | 4 | 5 | 2 | 6 | 6 |
| 37. SCOI37 | 3 | 2 | 4 | 3 | 3 | 4 | 6 | 5 | 5 | 4 | 2 | 4 | 5 | 4 | 4 | 6 |
| 38. SCOI38 | 4 | 5 | 5 | 4 | 4 | 5 | 7 | 8 | 4 | 7 | 3 | 5 | 6 | 5 | 7 | 7 |
| 39. SCOI39 | 3 | 4 | 4 | 3 | 3 | 4 | 6 | 7 | 7 | 6 | 2 | 4 | 5 | 4 | 6 | 6 |
| 40. SCOI40 | 3 | 4 | 4 | 3 | 3 | 4 | 6 | 5 | 7 | 6 | 2 | 4 | 5 | 4 | 6 | 6 |
| 41. SCOI41 | 3 | 4 | 4 | 3 | 3 | 4 | 6 | 7 | 7 | 6 | 2 | 4 | 5 | 4 | 6 | 6 |
| 42. SCOI42 | 2 | 3 | 3 | 2 | 2 | 3 | 3 | 6 | 6 | 5 | 1 | 3 | 4 | 3 | 5 | 5 |
| 43. SCOI43 | 2 | 3 | 3 | 2 | 2 | 3 | 5 | 6 | 6 | 5 | 1 | 3 | 4 | 3 | 5 | 5 |
| 44. SCOI44 | 4 | 3 | 5 | 4 | 4 | 5 | 7 | 6 | 6 | 5 | 3 | 5 | 6 | 5 | 3 | 5 |
| 45. SCOI45 | 3 | 4 | 4 | 3 | 3 | 4 | 6 | 7 | 7 | 4 | 2 | 4 | 5 | 4 | 6 | 6 |
| 46. SCOI46 | 2 | 3 | 3 | 2 | 2 | 3 | 5 | 6 | 6 | 5 | 1 | 3 | 4 | 3 | 5 | 5 |
| 47. SCOI47 | 3 | 4 | 4 | 3 | 3 | 4 | 6 | 7 | 7 | 6 | 2 | 4 | 5 | 4 | 6 | 6 |
| 48. SCOI48 | 2 | 3 | 3 | 2 | 2 | 3 | 5 | 6 | 6 | 5 | 1 | 3 | 4 | 3 | 5 | 5 |
| 49. SCOI49 | 2 | 3 | 3 | 2 | 2 | 3 | 5 | 6 | 6 | 5 | 1 | 3 | 4 | 3 | 5 | 5 |
| 50. SCOI50 | 2 | 3 | 3 | 2 | 2 | 3 | 5 | 6 | 6 | 5 | 1 | 3 | 4 | 3 | 5 | 5 |
| 51. SCOI51 | 3 | 4 | 4 | 3 | 3 | 4 | 6 | 7 | 7 | 6 | 2 | 4 | 5 | 4 | 6 | 6 |
| 52. SCOI52 | 3 | 2 | 4 | 3 | 3 | 4 | 6 | 5 | 5 | 4 | 2 | 4 | 5 | 4 | 2 | 6 |
| 53. SCOI53 | 2 | 3 | 3 | 2 | 2 | 3 | 5 | 6 | 6 | 5 | 1 | 3 | 4 | 3 | 5 | 5 |
| 54. SCOI54 | 2 | 3 | 3 | 2 | 2 | 3 | 5 | 6 | 6 | 5 | 1 | 3 | 4 | 3 | 5 | 5 |
| 55. SCOI55 | 2 | 3 | 3 | 2 | 2 | 3 | 5 | 6 | 6 | 5 | 1 | 3 | 4 | 3 | 5 | 5 |
| 56. SCOI56 | 4 | 3 | 5 | 4 | 4 | 5 | 7 | 6 | 6 | 4 | 3 | 5 | 6 | 5 | 5 | 7 |
| 57. SCOI57 | 4 | 5 | 5 | 4 | 4 | 5 | 7 | 8 | 8 | 7 | 3 | 5 | 6 | 5 | 5 | 7 |
| 58. SCOI58 | 4 | 5 | 5 | 4 | 4 | 5 | 7 | 8 | 8 | 7 | 3 | 5 | 6 | 5 | 7 | 7 |
| 59. SCOI59 | 2 | 3 | 3 | 2 | 2 | 3 | 5 | 6 | 6 | 5 | 1 | 3 | 4 | 3 | 5 | 5 |
| 60. SCOI60 | 4 | 3 | 3 | 4 | 2 | 3 | 7 | 8 | 8 | 5 | 3 | 5 | 6 | 3 | 7 | 5 |
| 61. SCOI61 | 2 | 1 | 3 | 2 | 2 | 3 | 5 | 4 | 4 | 3 | 1 | 3 | 4 | 3 | 3 | 5 |
| 62. SCOI62 | 4 | 5 | 5 | 4 | 4 | 5 | 7 | 6 | 8 | 7 | 3 | 5 | 6 | 5 | 7 | 7 |
| 63. SCOI63 | 4 | 3 | 5 | 4 | 4 | 5 | 5 | 6 | 6 | 4 | 3 | 5 | 4 | 5 | 3 | 7 |
| 64. SCOI64 | 3 | 4 | 4 | 3 | 3 | 4 | 6 | 7 | 7 | 6 | 2 | 4 | 5 | 4 | 6 | 6 |
| 65. SCOI65 | 3 | 4 | 4 | 3 | 3 | 4 | 6 | 7 | 7 | 6 | 2 | 4 | 5 | 4 | 6 | 6 |

| Haplotype | 17 | 18 | 19 | 20 | 21 | 22 | 23 | 24 | 25 | 26 | 27 | 28 | 29 | 30 | 31 | 32 |
| --- | --- | --- | --- | --- | --- | --- | --- | --- | --- | --- | --- | --- | --- | --- | --- | --- |
| 1. SCOI01 | 0.89 | 0.44 | 0.44 | 0.44 | 0.67 | 0.44 | 0.67 | 0.89 | 0.89 | 0.44 | 0.44 | 0.67 | 0.89 | 0.44 | 0.67 | 0.44 |
| 2. SCOI02 | 0.67 | 0.67 | 0.67 | 0.67 | 0.89 | 0.67 | 0.89 | 1.11 | 0.67 | 0.67 | 0.67 | 0.44 | 1.11 | 0.67 | 0.89 | 0.67 |
| 3. SCOI03 | 1.11 | 0.67 | 0.67 | 0.67 | 0.89 | 0.67 | 0.44 | 1.11 | 0.67 | 0.67 | 0.67 | 0.89 | 1.11 | 0.67 | 0.89 | 0.67 |
| 4. SCOI04 | 0.89 | 0.44 | 0.44 | 0.44 | 0.67 | 0.44 | 0.67 | 0.89 | 0.89 | 0.44 | 0.44 | 0.67 | 0.89 | 0.44 | 0.67 | 0.44 |
| 5. SCOI05 | 0.89 | 0.44 | 0.44 | 0.44 | 0.67 | 0.44 | 0.67 | 0.89 | 0.44 | 0.44 | 0.44 | 0.67 | 0.89 | 0.44 | 0.67 | 0.44 |
| 6. SCOI06 | 1.11 | 0.67 | 0.67 | 0.67 | 0.89 | 0.67 | 0.89 | 1.11 | 0.67 | 0.67 | 0.67 | 0.89 | 1.11 | 0.67 | 0.89 | 0.67 |
| 7. SCOI07 | 1.55 | 1.11 | 1.11 | 1.11 | 0.89 | 1.11 | 1.33 | 1.11 | 1.11 | 0.67 | 1.11 | 1.33 | 1.55 | 0.67 | 1.33 | 1.11 |
| 8. SCOI08 | 1.33 | 1.33 | 1.33 | 1.33 | 1.55 | 1.33 | 1.55 | 1.77 | 1.77 | 1.33 | 1.33 | 1.11 | 1.33 | 1.33 | 1.55 | 1.33 |
| 9. SCOI09 | 1.33 | 1.33 | 1.33 | 1.33 | 1.55 | 1.33 | 1.55 | 1.77 | 1.77 | 1.33 | 1.33 | 1.11 | 1.77 | 1.33 | 1.55 | 1.33 |
| 10. SCOI10 | 0.89 | 1.11 | 1.11 | 1.11 | 1.33 | 1.11 | 1.33 | 1.55 | 1.11 | 1.11 | 1.11 | 0.67 | 1.55 | 1.11 | 1.11 | 0.89 |
| 11. SCOI11 | 0.67 | 0.22 | 0.22 | 0.22 | 0.44 | 0.22 | 0.44 | 0.67 | 0.67 | 0.22 | 0.22 | 0.44 | 0.67 | 0.22 | 0.44 | 0.22 |
| 12. SCOI12 | 1.11 | 0.67 | 0.67 | 0.67 | 0.89 | 0.67 | 0.89 | 1.11 | 1.11 | 0.67 | 0.67 | 0.89 | 1.11 | 0.67 | 0.89 | 0.67 |
| 13. SCOI13 | 1.33 | 0.89 | 0.89 | 0.89 | 1.11 | 0.89 | 1.11 | 1.33 | 1.33 | 0.89 | 0.89 | 1.11 | 1.33 | 0.44 | 1.11 | 0.89 |
| 14. SCOI14 | 1.11 | 0.67 | 0.67 | 0.67 | 0.89 | 0.67 | 0.89 | 0.67 | 0.67 | 0.67 | 0.67 | 0.89 | 1.11 | 0.67 | 0.89 | 0.67 |
| 15. SCOI15 | 1.11 | 1.11 | 1.11 | 1.11 | 1.33 | 1.11 | 1.33 | 1.55 | 1.55 | 1.11 | 1.11 | 0.89 | 1.11 | 0.67 | 1.33 | 1.11 |
| 16. SCOI16 | 1.55 | 1.11 | 1.11 | 1.11 | 1.33 | 1.11 | 1.33 | 1.55 | 1.11 | 1.11 | 1.11 | 1.33 | 1.11 | 1.11 | 1.33 | 1.11 |
| 17. SCOI17 | - | 0.89 | 0.89 | 0.89 | 1.11 | 0.89 | 1.11 | 1.33 | 1.33 | 0.89 | 0.44 | 0.22 | 1.33 | 0.89 | 0.67 | 0.44 |
| 18. SCOI18 | 4 | - | 0.44 | 0.44 | 0.67 | 0.44 | 0.67 | 0.89 | 0.89 | 0.44 | 0.44 | 0.67 | 0.89 | 0.44 | 0.67 | 0.44 |
| 19. SCOI19 | 4 | 2 | - | 0.44 | 0.67 | 0.44 | 0.67 | 0.89 | 0.89 | 0.44 | 0.44 | 0.67 | 0.89 | 0.44 | 0.67 | 0.44 |
| 20. SCOI20 | 4 | 2 | 2 | - | 0.67 | 0.44 | 0.67 | 0.89 | 0.89 | 0.44 | 0.44 | 0.67 | 0.89 | 0.44 | 0.67 | 0.44 |
| 21. SCOI21 | 5 | 3 | 3 | 3 | - | 0.67 | 0.89 | 0.67 | 0.67 | 0.22 | 0.67 | 0.89 | 1.11 | 0.67 | 0.89 | 0.67 |
| 22. SCOI22 | 4 | 2 | 2 | 2 | 3 | - | 0.67 | 0.89 | 0.89 | 0.44 | 0.44 | 0.67 | 0.89 | 0.44 | 0.67 | 0.44 |
| 23. SCOI23 | 5 | 3 | 3 | 3 | 4 | 3 | - | 1.11 | 1.11 | 0.67 | 0.67 | 0.89 | 1.11 | 0.67 | 0.89 | 0.67 |
| 24. SCOI24 | 6 | 4 | 4 | 4 | 3 | 4 | 5 | - | 0.89 | 0.44 | 0.89 | 1.11 | 1.33 | 0.89 | 1.11 | 0.89 |
| 25. SCOI25 | 6 | 4 | 4 | 4 | 3 | 4 | 5 | 4 | - | 0.44 | 0.89 | 1.11 | 1.33 | 0.89 | 1.11 | 0.89 |
| 26. SCOI26 | 4 | 2 | 2 | 2 | 1 | 2 | 3 | 2 | 2 | - | 0.44 | 0.67 | 0.89 | 0.44 | 0.67 | 0.44 |
| 27. SCOI27 | 2 | 2 | 2 | 2 | 3 | 2 | 3 | 4 | 4 | 2 | - | 0.67 | 0.89 | 0.44 | 0.67 | 0.44 |
| 28. SCOI28 | 1 | 3 | 3 | 3 | 4 | 3 | 4 | 5 | 5 | 3 | 3 | - | 1.11 | 0.67 | 0.44 | 0.22 |
| 29. SCOI29 | 6 | 4 | 4 | 4 | 5 | 4 | 5 | 6 | 6 | 4 | 4 | 5 | - | 0.89 | 1.11 | 0.89 |
| 30. SCOI30 | 4 | 2 | 2 | 2 | 3 | 2 | 3 | 4 | 4 | 2 | 2 | 3 | 4 | - | 0.67 | 0.44 |
| 31. SCOI31 | 3 | 3 | 3 | 3 | 4 | 3 | 4 | 5 | 5 | 3 | 3 | 2 | 5 | 3 | - | 0.22 |
| 32. SCOI32 | 2 | 2 | 2 | 2 | 3 | 2 | 3 | 4 | 4 | 2 | 2 | 1 | 4 | 2 | 1 | - |
| 33. SCOI33 | 6 | 4 | 4 | 4 | 5 | 4 | 5 | 6 | 6 | 4 | 4 | 5 | 6 | 4 | 5 | 4 |
| 34. SCOI34 | 4 | 2 | 2 | 2 | 3 | 2 | 3 | 4 | 4 | 2 | 2 | 3 | 4 | 2 | 3 | 2 |
| 35. SCOI35 | 3 | 3 | 3 | 3 | 4 | 3 | 4 | 5 | 5 | 3 | 3 | 2 | 5 | 3 | 4 | 3 |
| 36. SCOI36 | 5 | 3 | 3 | 3 | 4 | 3 | 4 | 3 | 5 | 3 | 3 | 4 | 5 | 3 | 4 | 3 |
| 37. SCOI37 | 3 | 3 | 3 | 3 | 4 | 3 | 4 | 5 | 5 | 3 | 3 | 2 | 5 | 3 | 4 | 3 |
| 38. SCOI38 | 6 | 4 | 4 | 4 | 5 | 4 | 5 | 6 | 6 | 4 | 4 | 5 | 6 | 4 | 5 | 4 |
| 39. SCOI39 | 5 | 3 | 3 | 3 | 4 | 3 | 4 | 3 | 5 | 3 | 3 | 4 | 5 | 3 | 4 | 3 |
| 40. SCOI40 | 5 | 3 | 3 | 3 | 4 | 3 | 4 | 5 | 5 | 3 | 3 | 4 | 5 | 3 | 4 | 3 |
| 41. SCOI41 | 5 | 3 | 3 | 3 | 4 | 3 | 4 | 5 | 5 | 3 | 3 | 4 | 5 | 3 | 4 | 3 |
| 42. SCOI42 | 4 | 2 | 2 | 2 | 3 | 2 | 3 | 4 | 4 | 2 | 2 | 3 | 4 | 2 | 3 | 2 |
| 43. SCOI43 | 4 | 2 | 2 | 2 | 1 | 2 | 3 | 4 | 4 | 2 | 2 | 3 | 4 | 2 | 3 | 2 |
| 44. SCOI44 | 4 | 4 | 4 | 4 | 5 | 4 | 5 | 6 | 6 | 4 | 4 | 3 | 4 | 4 | 5 | 4 |
| 45. SCOI45 | 5 | 3 | 3 | 3 | 4 | 3 | 4 | 5 | 5 | 3 | 3 | 4 | 5 | 3 | 4 | 3 |
| 46. SCOI46 | 4 | 2 | 2 | 2 | 3 | 2 | 3 | 4 | 4 | 2 | 2 | 3 | 4 | 2 | 3 | 2 |
| 47. SCOI47 | 5 | 3 | 3 | 3 | 4 | 3 | 4 | 5 | 5 | 3 | 3 | 4 | 5 | 3 | 4 | 3 |
| 48. SCOI48 | 4 | 2 | 2 | 2 | 3 | 2 | 3 | 4 | 2 | 2 | 2 | 3 | 4 | 2 | 3 | 2 |
| 49. SCOI49 | 4 | 2 | 2 | 2 | 2 | 2 | 3 | 4 | 4 | 2 | 2 | 3 | 4 | 2 | 3 | 2 |
| 50. SCOI50 | 4 | 2 | 2 | 2 | 3 | 2 | 3 | 4 | 4 | 2 | 2 | 3 | 4 | 2 | 3 | 2 |
| 51. SCOI51 | 5 | 3 | 3 | 3 | 4 | 3 | 4 | 5 | 5 | 3 | 3 | 4 | 5 | 3 | 4 | 3 |
| 52. SCOI52 | 3 | 3 | 3 | 3 | 4 | 3 | 4 | 5 | 5 | 3 | 3 | 2 | 5 | 3 | 4 | 3 |
| 53. SCOI53 | 4 | 2 | 2 | 2 | 3 | 2 | 3 | 4 | 4 | 2 | 2 | 3 | 4 | 2 | 3 | 2 |
| 54. SCOI54 | 4 | 2 | 2 | 2 | 3 | 2 | 3 | 4 | 4 | 2 | 2 | 3 | 4 | 2 | 3 | 2 |
| 55. SCOI55 | 4 | 2 | 2 | 2 | 3 | 2 | 3 | 4 | 4 | 2 | 2 | 3 | 4 | 2 | 3 | 2 |
| 56. SCOI56 | 2 | 4 | 4 | 4 | 5 | 4 | 5 | 6 | 6 | 4 | 4 | 1 | 6 | 4 | 3 | 2 |
| 57. SCOI57 | 6 | 4 | 4 | 4 | 5 | 4 | 5 | 6 | 6 | 4 | 4 | 5 | 6 | 4 | 4 | 4 |
| 58. SCOI58 | 6 | 4 | 4 | 4 | 5 | 4 | 5 | 6 | 6 | 4 | 4 | 5 | 6 | 4 | 5 | 4 |
| 59. SCOI59 | 4 | 2 | 2 | 2 | 3 | 2 | 3 | 4 | 4 | 2 | 2 | 3 | 4 | 2 | 3 | 2 |
| 60. SCOI60 | 6 | 4 | 4 | 4 | 5 | 4 | 5 | 6 | 4 | 4 | 4 | 5 | 6 | 4 | 5 | 4 |
| 61. SCOI61 | 2 | 2 | 2 | 2 | 3 | 2 | 3 | 4 | 4 | 2 | 2 | 1 | 4 | 2 | 3 | 2 |
| 62. SCOI62 | 6 | 4 | 4 | 4 | 5 | 4 | 5 | 6 | 6 | 4 | 4 | 5 | 4 | 4 | 5 | 4 |
| 63. SCOI63 | 2 | 4 | 4 | 4 | 5 | 4 | 5 | 6 | 6 | 4 | 4 | 1 | 6 | 2 | 3 | 2 |
| 64. SCOI64 | 5 | 3 | 3 | 3 | 4 | 3 | 4 | 5 | 5 | 3 | 3 | 4 | 5 | 3 | 4 | 3 |
| 65. SCOI65 | 5 | 3 | 3 | 3 | 4 | 3 | 4 | 5 | 5 | 3 | 3 | 4 | 5 | 3 | 4 | 3 |

| Haplotype | 33 | 34 | 35 | 36 | 37 | 38 | 39 | 40 | 41 | 42 | 43 | 44 | 45 | 46 | 47 | 48 |
| --- | --- | --- | --- | --- | --- | --- | --- | --- | --- | --- | --- | --- | --- | --- | --- | --- |
| 1. SCOI01 | 0.89 | 0.44 | 0.67 | 0.67 | 0.67 | 0.89 | 0.67 | 0.67 | 0.67 | 0.44 | 0.44 | 0.89 | 0.67 | 0.44 | 0.67 | 0.44 |
| 2. SCOI02 | 1.11 | 0.67 | 0.44 | 0.89 | 0.44 | 1.11 | 0.89 | 0.89 | 0.89 | 0.67 | 0.67 | 0.67 | 0.89 | 0.67 | 0.89 | 0.67 |
| 3. SCOI03 | 1.11 | 0.67 | 0.89 | 0.89 | 0.89 | 1.11 | 0.89 | 0.89 | 0.89 | 0.67 | 0.67 | 1.11 | 0.89 | 0.67 | 0.89 | 0.67 |
| 4. SCOI04 | 0.89 | 0.44 | 0.67 | 0.67 | 0.67 | 0.89 | 0.67 | 0.67 | 0.67 | 0.44 | 0.44 | 0.89 | 0.67 | 0.44 | 0.67 | 0.44 |
| 5. SCOI05 | 0.89 | 0.44 | 0.67 | 0.67 | 0.67 | 0.89 | 0.67 | 0.67 | 0.67 | 0.44 | 0.44 | 0.89 | 0.67 | 0.44 | 0.67 | 0.44 |
| 6. SCOI06 | 0.89 | 0.67 | 0.89 | 0.89 | 0.89 | 1.11 | 0.89 | 0.89 | 0.89 | 0.67 | 0.67 | 1.11 | 0.89 | 0.67 | 0.89 | 0.67 |
| 7. SCOI07 | 1.55 | 1.11 | 1.33 | 1.33 | 1.33 | 1.55 | 1.33 | 1.33 | 1.33 | 0.67 | 1.11 | 1.55 | 1.33 | 1.11 | 1.33 | 1.11 |
| 8. SCOI08 | 1.77 | 1.33 | 0.67 | 1.55 | 1.11 | 1.77 | 1.55 | 1.11 | 1.55 | 1.33 | 1.33 | 1.33 | 1.55 | 1.33 | 1.55 | 1.33 |
| 9. SCOI09 | 1.77 | 1.33 | 1.11 | 1.55 | 1.11 | 0.89 | 1.55 | 1.55 | 1.55 | 1.33 | 1.33 | 1.33 | 1.55 | 1.33 | 1.55 | 1.33 |
| 10. SCOI10 | 1.55 | 1.11 | 0.89 | 1.33 | 0.89 | 1.55 | 1.33 | 1.33 | 1.33 | 1.11 | 1.11 | 1.11 | 0.89 | 1.11 | 1.33 | 1.11 |
| 11. SCOI11 | 0.67 | 0.22 | 0.44 | 0.44 | 0.44 | 0.67 | 0.44 | 0.44 | 0.44 | 0.22 | 0.22 | 0.67 | 0.44 | 0.22 | 0.44 | 0.22 |
| 12. SCOI12 | 1.11 | 0.67 | 0.89 | 0.89 | 0.89 | 1.11 | 0.89 | 0.89 | 0.89 | 0.67 | 0.67 | 1.11 | 0.89 | 0.67 | 0.89 | 0.67 |
| 13. SCOI13 | 1.33 | 0.89 | 1.11 | 1.11 | 1.11 | 1.33 | 1.11 | 1.11 | 1.11 | 0.89 | 0.89 | 1.33 | 1.11 | 0.89 | 1.11 | 0.89 |
| 14. SCOI14 | 1.11 | 0.67 | 0.89 | 0.44 | 0.89 | 1.11 | 0.89 | 0.89 | 0.89 | 0.67 | 0.67 | 1.11 | 0.89 | 0.67 | 0.89 | 0.67 |
| 15. SCOI15 | 1.55 | 1.11 | 0.89 | 1.33 | 0.89 | 1.55 | 1.33 | 1.33 | 1.33 | 1.11 | 1.11 | 0.67 | 1.33 | 1.11 | 1.33 | 1.11 |
| 16. SCOI16 | 1.55 | 1.11 | 1.33 | 1.33 | 1.33 | 1.55 | 1.33 | 1.33 | 1.33 | 1.11 | 1.11 | 1.11 | 1.33 | 1.11 | 1.33 | 1.11 |
| 17. SCOI17 | 1.33 | 0.89 | 0.67 | 1.11 | 0.67 | 1.33 | 1.11 | 1.11 | 1.11 | 0.89 | 0.89 | 0.89 | 1.11 | 0.89 | 1.11 | 0.89 |
| 18. SCOI18 | 0.89 | 0.44 | 0.67 | 0.67 | 0.67 | 0.89 | 0.67 | 0.67 | 0.67 | 0.44 | 0.44 | 0.89 | 0.67 | 0.44 | 0.67 | 0.44 |
| 19. SCOI19 | 0.89 | 0.44 | 0.67 | 0.67 | 0.67 | 0.89 | 0.67 | 0.67 | 0.67 | 0.44 | 0.44 | 0.89 | 0.67 | 0.44 | 0.67 | 0.44 |
| 20. SCOI20 | 0.89 | 0.44 | 0.67 | 0.67 | 0.67 | 0.89 | 0.67 | 0.67 | 0.67 | 0.44 | 0.44 | 0.89 | 0.67 | 0.44 | 0.67 | 0.44 |
| 21. SCOI21 | 1.11 | 0.67 | 0.89 | 0.89 | 0.89 | 1.11 | 0.89 | 0.89 | 0.89 | 0.67 | 0.22 | 1.11 | 0.89 | 0.67 | 0.89 | 0.67 |
| 22. SCOI22 | 0.89 | 0.44 | 0.67 | 0.67 | 0.67 | 0.89 | 0.67 | 0.67 | 0.67 | 0.44 | 0.44 | 0.89 | 0.67 | 0.44 | 0.67 | 0.44 |
| 23. SCOI23 | 1.11 | 0.67 | 0.89 | 0.89 | 0.89 | 1.11 | 0.89 | 0.89 | 0.89 | 0.67 | 0.67 | 1.11 | 0.89 | 0.67 | 0.89 | 0.67 |
| 24. SCOI24 | 1.33 | 0.89 | 1.11 | 0.67 | 1.11 | 1.33 | 0.67 | 1.11 | 1.11 | 0.89 | 0.89 | 1.33 | 1.11 | 0.89 | 1.11 | 0.89 |
| 25. SCOI25 | 1.33 | 0.89 | 1.11 | 1.11 | 1.11 | 1.33 | 1.11 | 1.11 | 1.11 | 0.89 | 0.89 | 1.33 | 1.11 | 0.89 | 1.11 | 0.44 |
| 26. SCOI26 | 0.89 | 0.44 | 0.67 | 0.67 | 0.67 | 0.89 | 0.67 | 0.67 | 0.67 | 0.44 | 0.44 | 0.89 | 0.67 | 0.44 | 0.67 | 0.44 |
| 27. SCOI27 | 0.89 | 0.44 | 0.67 | 0.67 | 0.67 | 0.89 | 0.67 | 0.67 | 0.67 | 0.44 | 0.44 | 0.89 | 0.67 | 0.44 | 0.67 | 0.44 |
| 28. SCOI28 | 1.11 | 0.67 | 0.44 | 0.89 | 0.44 | 1.11 | 0.89 | 0.89 | 0.89 | 0.67 | 0.67 | 0.67 | 0.89 | 0.67 | 0.89 | 0.67 |
| 29. SCOI29 | 1.33 | 0.89 | 1.11 | 1.11 | 1.11 | 1.33 | 1.11 | 1.11 | 1.11 | 0.89 | 0.89 | 0.89 | 1.11 | 0.89 | 1.11 | 0.89 |
| 30. SCOI30 | 0.89 | 0.44 | 0.67 | 0.67 | 0.67 | 0.89 | 0.67 | 0.67 | 0.67 | 0.44 | 0.44 | 0.89 | 0.67 | 0.44 | 0.67 | 0.44 |
| 31. SCOI31 | 1.11 | 0.67 | 0.89 | 0.89 | 0.89 | 1.11 | 0.89 | 0.89 | 0.89 | 0.67 | 0.67 | 1.11 | 0.89 | 0.67 | 0.89 | 0.67 |
| 32. SCOI32 | 0.89 | 0.44 | 0.67 | 0.67 | 0.67 | 0.89 | 0.67 | 0.67 | 0.67 | 0.44 | 0.44 | 0.89 | 0.67 | 0.44 | 0.67 | 0.44 |
| 33. SCOI33 | - | 0.89 | 1.11 | 1.11 | 1.11 | 1.33 | 1.11 | 1.11 | 1.11 | 0.89 | 0.89 | 1.33 | 1.11 | 0.89 | 1.11 | 0.89 |
| 34. SCOI34 | 4 | - | 0.67 | 0.67 | 0.67 | 0.89 | 0.67 | 0.67 | 0.67 | 0.44 | 0.44 | 0.89 | 0.67 | 0.44 | 0.67 | 0.44 |
| 35. SCOI35 | 5 | 3 | - | 0.89 | 0.44 | 1.11 | 0.89 | 0.44 | 0.89 | 0.67 | 0.67 | 0.67 | 0.89 | 0.67 | 0.89 | 0.67 |
| 36. SCOI36 | 5 | 3 | 4 | - | 0.89 | 1.11 | 0.89 | 0.89 | 0.89 | 0.67 | 0.67 | 1.11 | 0.89 | 0.67 | 0.89 | 0.67 |
| 37. SCOI37 | 5 | 3 | 2 | 4 | - | 1.11 | 0.89 | 0.89 | 0.89 | 0.67 | 0.67 | 0.67 | 0.89 | 0.67 | 0.89 | 0.67 |
| 38. SCOI38 | 6 | 4 | 5 | 5 | 5 | - | 1.11 | 1.11 | 1.11 | 0.89 | 0.89 | 1.33 | 1.11 | 0.89 | 1.11 | 0.89 |
| 39. SCOI39 | 5 | 3 | 4 | 4 | 4 | 5 | - | 0.89 | 0.89 | 0.67 | 0.67 | 1.11 | 0.89 | 0.67 | 0.44 | 0.67 |
| 40. SCOI40 | 5 | 3 | 2 | 4 | 4 | 5 | 4 | - | 0.89 | 0.67 | 0.67 | 1.11 | 0.89 | 0.67 | 0.89 | 0.67 |
| 41. SCOI41 | 5 | 3 | 4 | 4 | 4 | 5 | 4 | 4 | - | 0.67 | 0.67 | 1.11 | 0.89 | 0.67 | 0.89 | 0.67 |
| 42. SCOI42 | 4 | 2 | 3 | 3 | 3 | 4 | 3 | 3 | 3 | - | 0.44 | 0.89 | 0.67 | 0.44 | 0.67 | 0.44 |
| 43. SCOI43 | 4 | 2 | 3 | 3 | 3 | 4 | 3 | 3 | 3 | 2 | - | 0.89 | 0.67 | 0.44 | 0.67 | 0.44 |
| 44. SCOI44 | 6 | 4 | 3 | 5 | 3 | 6 | 5 | 5 | 5 | 4 | 4 | - | 1.11 | 0.89 | 1.11 | 0.89 |
| 45. SCOI45 | 5 | 3 | 4 | 4 | 4 | 5 | 4 | 4 | 4 | 3 | 3 | 5 | - | 0.67 | 0.89 | 0.67 |
| 46. SCOI46 | 4 | 2 | 3 | 3 | 3 | 4 | 3 | 3 | 3 | 2 | 2 | 4 | 3 | - | 0.67 | 0.44 |
| 47. SCOI47 | 5 | 3 | 4 | 4 | 4 | 5 | 2 | 4 | 4 | 3 | 3 | 5 | 4 | 3 | - | 0.67 |
| 48. SCOI48 | 4 | 2 | 3 | 3 | 3 | 4 | 3 | 3 | 3 | 2 | 2 | 4 | 3 | 2 | 3 | - |
| 49. SCOI49 | 4 | 2 | 3 | 3 | 3 | 4 | 3 | 3 | 3 | 2 | 1 | 4 | 3 | 2 | 3 | 2 |
| 50. SCOI50 | 4 | 2 | 3 | 3 | 3 | 4 | 3 | 3 | 3 | 2 | 2 | 4 | 3 | 2 | 3 | 2 |
| 51. SCOI51 | 3 | 3 | 4 | 4 | 4 | 5 | 4 | 4 | 2 | 3 | 3 | 5 | 4 | 3 | 4 | 3 |
| 52. SCOI52 | 5 | 3 | 2 | 4 | 2 | 5 | 4 | 4 | 4 | 3 | 3 | 3 | 4 | 3 | 4 | 3 |
| 53. SCOI53 | 4 | 2 | 3 | 3 | 3 | 4 | 3 | 1 | 3 | 2 | 2 | 4 | 3 | 2 | 3 | 2 |
| 54. SCOI54 | 4 | 2 | 3 | 3 | 3 | 4 | 3 | 3 | 3 | 2 | 2 | 4 | 3 | 2 | 3 | 2 |
| 55. SCOI55 | 4 | 2 | 3 | 3 | 3 | 4 | 3 | 3 | 3 | 2 | 2 | 4 | 3 | 2 | 3 | 2 |
| 56. SCOI56 | 6 | 4 | 3 | 5 | 3 | 6 | 5 | 5 | 5 | 4 | 4 | 4 | 5 | 4 | 5 | 4 |
| 57. SCOI57 | 6 | 2 | 5 | 5 | 5 | 6 | 5 | 5 | 5 | 4 | 4 | 6 | 5 | 4 | 5 | 4 |
| 58. SCOI58 | 6 | 4 | 5 | 5 | 5 | 4 | 5 | 5 | 5 | 4 | 4 | 6 | 5 | 4 | 5 | 4 |
| 59. SCOI59 | 4 | 2 | 3 | 3 | 3 | 4 | 3 | 3 | 3 | 2 | 2 | 4 | 3 | 2 | 3 | 2 |
| 60. SCOI60 | 6 | 4 | 5 | 5 | 5 | 6 | 3 | 5 | 5 | 4 | 4 | 6 | 5 | 4 | 3 | 4 |
| 61. SCOI61 | 4 | 2 | 1 | 3 | 1 | 4 | 3 | 3 | 3 | 2 | 2 | 2 | 3 | 2 | 3 | 2 |
| 62. SCOI62 | 6 | 2 | 5 | 5 | 5 | 6 | 5 | 5 | 5 | 4 | 4 | 6 | 5 | 4 | 5 | 4 |
| 63. SCOI63 | 6 | 4 | 3 | 5 | 3 | 6 | 5 | 5 | 5 | 4 | 4 | 4 | 5 | 4 | 5 | 4 |
| 64. SCOI64 | 5 | 3 | 4 | 4 | 4 | 5 | 4 | 4 | 4 | 3 | 3 | 5 | 4 | 3 | 4 | 3 |
| 65. SCOI65 | 5 | 3 | 4 | 4 | 4 | 5 | 4 | 4 | 4 | 3 | 3 | 5 | 4 | 3 | 4 | 3 |

| Haplotype | 49 | 50 | 51 | 52 | 53 | 54 | 55 | 56 | 57 | 58 | 59 | 60 | 61 | 62 | 63 | 64 | 65 |
| --- | --- | --- | --- | --- | --- | --- | --- | --- | --- | --- | --- | --- | --- | --- | --- | --- | --- |
| 1. SCOI01 | 0.44 | 0.44 | 0.67 | 0.67 | 0.44 | 0.44 | 0.44 | 0.89 | 0.89 | 0.89 | 0.44 | 0.89 | 0.44 | 0.89 | 0.89 | 0.67 | 0.67 |
| 2. SCOI02 | 0.67 | 0.67 | 0.89 | 0.44 | 0.67 | 0.67 | 0.67 | 0.67 | 1.11 | 1.11 | 0.67 | 0.67 | 0.22 | 1.11 | 0.67 | 0.89 | 0.89 |
| 3. SCOI03 | 0.67 | 0.67 | 0.89 | 0.89 | 0.67 | 0.67 | 0.67 | 1.11 | 1.11 | 1.11 | 0.67 | 0.67 | 0.67 | 1.11 | 1.11 | 0.89 | 0.89 |
| 4. SCOI04 | 0.44 | 0.44 | 0.67 | 0.67 | 0.44 | 0.44 | 0.44 | 0.89 | 0.89 | 0.89 | 0.44 | 0.89 | 0.44 | 0.89 | 0.89 | 0.67 | 0.67 |
| 5. SCOI05 | 0.44 | 0.44 | 0.67 | 0.67 | 0.44 | 0.44 | 0.44 | 0.89 | 0.89 | 0.89 | 0.44 | 0.44 | 0.44 | 0.89 | 0.89 | 0.67 | 0.67 |
| 6. SCOI06 | 0.67 | 0.67 | 0.89 | 0.89 | 0.67 | 0.67 | 0.67 | 1.11 | 1.11 | 1.11 | 0.67 | 0.67 | 0.67 | 1.11 | 1.11 | 0.89 | 0.89 |
| 7. SCOI07 | 1.11 | 1.11 | 1.33 | 1.33 | 1.11 | 1.11 | 1.11 | 1.55 | 1.55 | 1.55 | 1.11 | 1.55 | 1.11 | 1.55 | 1.11 | 1.33 | 1.33 |
| 8. SCOI08 | 1.33 | 1.33 | 1.55 | 1.11 | 1.33 | 1.33 | 1.33 | 1.33 | 1.77 | 1.77 | 1.33 | 1.77 | 0.89 | 1.33 | 1.33 | 1.55 | 1.55 |
| 9. SCOI09 | 1.33 | 1.33 | 1.55 | 1.11 | 1.33 | 1.33 | 1.33 | 1.33 | 1.77 | 1.77 | 1.33 | 1.77 | 0.89 | 1.77 | 1.33 | 1.55 | 1.55 |
| 10. SCOI10 | 1.11 | 1.11 | 1.33 | 0.89 | 1.11 | 1.11 | 1.11 | 0.89 | 1.55 | 1.55 | 1.11 | 1.11 | 0.67 | 1.55 | 0.89 | 1.33 | 1.33 |
| 11. SCOI11 | 0.22 | 0.22 | 0.44 | 0.44 | 0.22 | 0.22 | 0.22 | 0.67 | 0.67 | 0.67 | 0.22 | 0.67 | 0.22 | 0.67 | 0.67 | 0.44 | 0.44 |
| 12. SCOI12 | 0.67 | 0.67 | 0.89 | 0.89 | 0.67 | 0.67 | 0.67 | 1.11 | 1.11 | 1.11 | 0.67 | 1.11 | 0.67 | 1.11 | 1.11 | 0.89 | 0.89 |
| 13. SCOI13 | 0.89 | 0.89 | 1.11 | 1.11 | 0.89 | 0.89 | 0.89 | 1.33 | 1.33 | 1.33 | 0.89 | 1.33 | 0.89 | 1.33 | 0.89 | 1.11 | 1.11 |
| 14. SCOI14 | 0.67 | 0.67 | 0.89 | 0.89 | 0.67 | 0.67 | 0.67 | 1.11 | 1.11 | 1.11 | 0.67 | 0.67 | 0.67 | 1.11 | 1.11 | 0.89 | 0.89 |
| 15. SCOI15 | 1.11 | 1.11 | 1.33 | 0.44 | 1.11 | 1.11 | 1.11 | 1.11 | 1.11 | 1.55 | 1.11 | 1.55 | 0.67 | 1.55 | 0.67 | 1.33 | 1.33 |
| 16. SCOI16 | 1.11 | 1.11 | 1.33 | 1.33 | 1.11 | 1.11 | 1.11 | 1.55 | 1.55 | 1.55 | 1.11 | 1.11 | 1.11 | 1.55 | 1.55 | 1.33 | 1.33 |
| 17. SCOI17 | 0.89 | 0.89 | 1.11 | 0.67 | 0.89 | 0.89 | 0.89 | 0.44 | 1.33 | 1.33 | 0.89 | 1.33 | 0.44 | 1.33 | 0.44 | 1.11 | 1.11 |
| 18. SCOI18 | 0.44 | 0.44 | 0.67 | 0.67 | 0.44 | 0.44 | 0.44 | 0.89 | 0.89 | 0.89 | 0.44 | 0.89 | 0.44 | 0.89 | 0.89 | 0.67 | 0.67 |
| 19. SCOI19 | 0.44 | 0.44 | 0.67 | 0.67 | 0.44 | 0.44 | 0.44 | 0.89 | 0.89 | 0.89 | 0.44 | 0.89 | 0.44 | 0.89 | 0.89 | 0.67 | 0.67 |
| 20. SCOI20 | 0.44 | 0.44 | 0.67 | 0.67 | 0.44 | 0.44 | 0.44 | 0.89 | 0.89 | 0.89 | 0.44 | 0.89 | 0.44 | 0.89 | 0.89 | 0.67 | 0.67 |
| 21. SCOI21 | 0.44 | 0.67 | 0.89 | 0.89 | 0.67 | 0.67 | 0.67 | 1.11 | 1.11 | 1.11 | 0.67 | 1.11 | 0.67 | 1.11 | 1.11 | 0.89 | 0.89 |
| 22. SCOI22 | 0.44 | 0.44 | 0.67 | 0.67 | 0.44 | 0.44 | 0.44 | 0.89 | 0.89 | 0.89 | 0.44 | 0.89 | 0.44 | 0.89 | 0.89 | 0.67 | 0.67 |
| 23. SCOI23 | 0.67 | 0.67 | 0.89 | 0.89 | 0.67 | 0.67 | 0.67 | 1.11 | 1.11 | 1.11 | 0.67 | 1.11 | 0.67 | 1.11 | 1.11 | 0.89 | 0.89 |
| 24. SCOI24 | 0.89 | 0.89 | 1.11 | 1.11 | 0.89 | 0.89 | 0.89 | 1.33 | 1.33 | 1.33 | 0.89 | 1.33 | 0.89 | 1.33 | 1.33 | 1.11 | 1.11 |
| 25. SCOI25 | 0.89 | 0.89 | 1.11 | 1.11 | 0.89 | 0.89 | 0.89 | 1.33 | 1.33 | 1.33 | 0.89 | 0.89 | 0.89 | 1.33 | 1.33 | 1.11 | 1.11 |
| 26. SCOI26 | 0.44 | 0.44 | 0.67 | 0.67 | 0.44 | 0.44 | 0.44 | 0.89 | 0.89 | 0.89 | 0.44 | 0.89 | 0.44 | 0.89 | 0.89 | 0.67 | 0.67 |
| 27. SCOI27 | 0.44 | 0.44 | 0.67 | 0.67 | 0.44 | 0.44 | 0.44 | 0.89 | 0.89 | 0.89 | 0.44 | 0.89 | 0.44 | 0.89 | 0.89 | 0.67 | 0.67 |
| 28. SCOI28 | 0.67 | 0.67 | 0.89 | 0.44 | 0.67 | 0.67 | 0.67 | 0.22 | 1.11 | 1.11 | 0.67 | 1.11 | 0.22 | 1.11 | 0.22 | 0.89 | 0.89 |
| 29. SCOI29 | 0.89 | 0.89 | 1.11 | 1.11 | 0.89 | 0.89 | 0.89 | 1.33 | 1.33 | 1.33 | 0.89 | 1.33 | 0.89 | 0.89 | 1.33 | 1.11 | 1.11 |
| 30. SCOI30 | 0.44 | 0.44 | 0.67 | 0.67 | 0.44 | 0.44 | 0.44 | 0.89 | 0.89 | 0.89 | 0.44 | 0.89 | 0.44 | 0.89 | 0.44 | 0.67 | 0.67 |
| 31. SCOI31 | 0.67 | 0.67 | 0.89 | 0.89 | 0.67 | 0.67 | 0.67 | 0.67 | 0.89 | 1.11 | 0.67 | 1.11 | 0.67 | 1.11 | 0.67 | 0.89 | 0.89 |
| 32. SCOI32 | 0.44 | 0.44 | 0.67 | 0.67 | 0.44 | 0.44 | 0.44 | 0.44 | 0.89 | 0.89 | 0.44 | 0.89 | 0.44 | 0.89 | 0.44 | 0.67 | 0.67 |
| 33. SCOI33 | 0.89 | 0.89 | 0.67 | 1.11 | 0.89 | 0.89 | 0.89 | 1.33 | 1.33 | 1.33 | 0.89 | 1.33 | 0.89 | 1.33 | 1.33 | 1.11 | 1.11 |
| 34. SCOI34 | 0.44 | 0.44 | 0.67 | 0.67 | 0.44 | 0.44 | 0.44 | 0.89 | 0.44 | 0.89 | 0.44 | 0.89 | 0.44 | 0.44 | 0.89 | 0.67 | 0.67 |
| 35. SCOI35 | 0.67 | 0.67 | 0.89 | 0.44 | 0.67 | 0.67 | 0.67 | 0.67 | 1.11 | 1.11 | 0.67 | 1.11 | 0.22 | 1.11 | 0.67 | 0.89 | 0.89 |
| 36. SCOI36 | 0.67 | 0.67 | 0.89 | 0.89 | 0.67 | 0.67 | 0.67 | 1.11 | 1.11 | 1.11 | 0.67 | 1.11 | 0.67 | 1.11 | 1.11 | 0.89 | 0.89 |
| 37. SCOI37 | 0.67 | 0.67 | 0.89 | 0.44 | 0.67 | 0.67 | 0.67 | 0.67 | 1.11 | 1.11 | 0.67 | 1.11 | 0.22 | 1.11 | 0.67 | 0.89 | 0.89 |
| 38. SCOI38 | 0.89 | 0.89 | 1.11 | 1.11 | 0.89 | 0.89 | 0.89 | 1.33 | 1.33 | 0.89 | 0.89 | 1.33 | 0.89 | 1.33 | 1.33 | 1.11 | 1.11 |
| 39. SCOI39 | 0.67 | 0.67 | 0.89 | 0.89 | 0.67 | 0.67 | 0.67 | 1.11 | 1.11 | 1.11 | 0.67 | 0.67 | 0.67 | 1.11 | 1.11 | 0.89 | 0.89 |
| 40. SCOI40 | 0.67 | 0.67 | 0.89 | 0.89 | 0.22 | 0.67 | 0.67 | 1.11 | 1.11 | 1.11 | 0.67 | 1.11 | 0.67 | 1.11 | 1.11 | 0.89 | 0.89 |
| 41. SCOI41 | 0.67 | 0.67 | 0.44 | 0.89 | 0.67 | 0.67 | 0.67 | 1.11 | 1.11 | 1.11 | 0.67 | 1.11 | 0.67 | 1.11 | 1.11 | 0.89 | 0.89 |
| 42. SCOI42 | 0.44 | 0.44 | 0.67 | 0.67 | 0.44 | 0.44 | 0.44 | 0.89 | 0.89 | 0.89 | 0.44 | 0.89 | 0.44 | 0.89 | 0.89 | 0.67 | 0.67 |
| 43. SCOI43 | 0.22 | 0.44 | 0.67 | 0.67 | 0.44 | 0.44 | 0.44 | 0.89 | 0.89 | 0.89 | 0.44 | 0.89 | 0.44 | 0.89 | 0.89 | 0.67 | 0.67 |
| 44. SCOI44 | 0.89 | 0.89 | 1.11 | 0.67 | 0.89 | 0.89 | 0.89 | 0.89 | 1.33 | 1.33 | 0.89 | 1.33 | 0.44 | 1.33 | 0.89 | 1.11 | 1.11 |
| 45. SCOI45 | 0.67 | 0.67 | 0.89 | 0.89 | 0.67 | 0.67 | 0.67 | 1.11 | 1.11 | 1.11 | 0.67 | 1.11 | 0.67 | 1.11 | 1.11 | 0.89 | 0.89 |
| 46. SCOI46 | 0.44 | 0.44 | 0.67 | 0.67 | 0.44 | 0.44 | 0.44 | 0.89 | 0.89 | 0.89 | 0.44 | 0.89 | 0.44 | 0.89 | 0.89 | 0.67 | 0.67 |
| 47. SCOI47 | 0.67 | 0.67 | 0.89 | 0.89 | 0.67 | 0.67 | 0.67 | 1.11 | 1.11 | 1.11 | 0.67 | 0.67 | 0.67 | 1.11 | 1.11 | 0.89 | 0.89 |
| 48. SCOI48 | 0.44 | 0.44 | 0.67 | 0.67 | 0.44 | 0.44 | 0.44 | 0.89 | 0.89 | 0.89 | 0.44 | 0.89 | 0.44 | 0.89 | 0.89 | 0.67 | 0.67 |
| 49. SCOI49 | - | 0.44 | 0.67 | 0.67 | 0.44 | 0.44 | 0.44 | 0.89 | 0.89 | 0.89 | 0.44 | 0.89 | 0.44 | 0.89 | 0.89 | 0.67 | 0.67 |
| 50. SCOI50 | 2 | - | 0.67 | 0.67 | 0.44 | 0.44 | 0.44 | 0.89 | 0.89 | 0.89 | 0.44 | 0.89 | 0.44 | 0.89 | 0.89 | 0.67 | 0.67 |
| 51. SCOI51 | 3 | 3 | - | 0.89 | 0.67 | 0.67 | 0.67 | 1.11 | 1.11 | 1.11 | 0.67 | 1.11 | 0.67 | 1.11 | 1.11 | 0.89 | 0.89 |
| 52. SCOI52 | 3 | 3 | 4 | - | 0.67 | 0.67 | 0.67 | 0.67 | 0.67 | 1.11 | 0.67 | 1.11 | 0.22 | 1.11 | 0.67 | 0.89 | 0.89 |
| 53. SCOI53 | 2 | 2 | 3 | 3 | - | 0.44 | 0.44 | 0.89 | 0.89 | 0.89 | 0.44 | 0.89 | 0.44 | 0.89 | 0.89 | 0.67 | 0.67 |
| 54. SCOI54 | 2 | 2 | 3 | 3 | 2 | - | 0.44 | 0.89 | 0.89 | 0.89 | 0.44 | 0.89 | 0.44 | 0.89 | 0.89 | 0.67 | 0.67 |
| 55. SCOI55 | 2 | 2 | 3 | 3 | 2 | 2 | - | 0.89 | 0.89 | 0.89 | 0.44 | 0.89 | 0.44 | 0.89 | 0.89 | 0.67 | 0.67 |
| 56. SCOI56 | 4 | 4 | 5 | 3 | 4 | 4 | 4 | - | 1.33 | 1.33 | 0.89 | 1.33 | 0.44 | 1.33 | 0.44 | 0.67 | 1.11 |
| 57. SCOI57 | 4 | 4 | 5 | 3 | 4 | 4 | 4 | 6 | - | 1.33 | 0.89 | 1.33 | 0.89 | 0.89 | 1.33 | 1.11 | 1.11 |
| 58. SCOI58 | 4 | 4 | 5 | 5 | 4 | 4 | 4 | 6 | 6 | - | 0.89 | 1.33 | 0.89 | 1.33 | 1.33 | 1.11 | 1.11 |
| 59. SCOI59 | 2 | 2 | 3 | 3 | 2 | 2 | 2 | 4 | 4 | 4 | - | 0.89 | 0.44 | 0.89 | 0.89 | 0.67 | 0.67 |
| 60. SCOI60 | 4 | 4 | 5 | 5 | 4 | 4 | 4 | 6 | 6 | 6 | 4 | - | 0.89 | 1.33 | 1.33 | 1.11 | 1.11 |
| 61. SCOI61 | 2 | 2 | 3 | 1 | 2 | 2 | 2 | 2 | 4 | 4 | 2 | 4 | - | 0.89 | 0.44 | 0.67 | 0.67 |
| 62. SCOI62 | 4 | 4 | 5 | 5 | 4 | 4 | 4 | 6 | 4 | 6 | 4 | 6 | 4 | - | 1.33 | 1.11 | 1.11 |
| 63. SCOI63 | 4 | 4 | 5 | 3 | 4 | 4 | 4 | 2 | 6 | 6 | 4 | 6 | 2 | 6 | - | 1.11 | 1.11 |
| 64. SCOI64 | 3 | 3 | 4 | 4 | 3 | 3 | 3 | 3 | 5 | 5 | 3 | 5 | 3 | 5 | 5 | - | 0.89 |
| 65. SCOI65 | 3 | 3 | 4 | 4 | 3 | 3 | 3 | 5 | 5 | 5 | 3 | 5 | 3 | 5 | 5 | 4 | - |

Numbers above the diagonal are percent distance values; numbers below the diagonal are absolute distance values.
