# Supplementary material for: Identification of Sympetrum depressiusculum Sélys, 1841 in South Korea (Odonata: Libellulidae) According to Morphology and Genetic Markers
Source: Insects. 2023 Aug 30;14(9):733. doi: 10.3390/insects14090733 (PMC10531817; doi:10.3390/insects14090733)
Supplement: Supplementary file 1 [file insects-14-00733-s001.zip › Table S9. PC-Our 16S+GB 16S.docx]

**Table S9.** Pairwise comparisons of *16S rRNA* haplotypes of *Sympetrum* species sequenced in this study and collected from public data.

| Haplotype | 1 | 2 | 3 | 4 | 5 | 6 | 7 | 8 | 9 | 10 | 11 | 12 | 13 | 14 | 15 | 16 | 17 | 18 | 19 |
| --- | --- | --- | --- | --- | --- | --- | --- | --- | --- | --- | --- | --- | --- | --- | --- | --- | --- | --- | --- |
| 1. S16S01 | - | 0.36 | 0.36 | 0.36 | 0.36 | 0.36 | 0.36 | 0.36 | 0.73 | 0.73 | 0.36 | 0.36 | 0.73 | 0.36 | 0.36 | 0.36 | 0.36 | 0.36 | 0.36 |
| 2. S16S02 | 1 | - | 0.36 | 0.73 | 0.73 | 0.73 | 0.73 | 0.73 | 1.09 | 1.09 | 0.73 | 0.73 | 1.09 | 0.73 | 0.73 | 0.73 | 0.73 | 0.73 | 0.73 |
| 3. S16S03 | 1 | 1 | - | 0.73 | 0.73 | 0.73 | 0.73 | 0.73 | 1.09 | 1.09 | 0.73 | 0.73 | 1.09 | 0.73 | 0.73 | 0.73 | 0.73 | 0.73 | 0.73 |
| 4. S16S04 | 1 | 2 | 2 | - | 0.73 | 0.73 | 0.73 | 0.73 | 1.09 | 1.09 | 0.73 | 0.73 | 0.36 | 0.73 | 0.73 | 0.73 | 0.73 | 0.73 | 0.73 |
| 5. S16S05 | 1 | 2 | 2 | 2 | - | 0.73 | 0.73 | 0.73 | 1.09 | 1.09 | 0.73 | 0.73 | 1.09 | 0.73 | 0.73 | 0.73 | 0.73 | 0.73 | 0.73 |
| 6. S16S06 | 1 | 2 | 2 | 2 | 2 | - | 0.73 | 0.73 | 1.09 | 1.09 | 0.73 | 0.73 | 1.09 | 0.73 | 0.73 | 0.73 | 0.73 | 0.73 | 0.73 |
| 7. S16S07 | 1 | 2 | 2 | 2 | 2 | 2 | - | 0.73 | 1.09 | 1.09 | 0.73 | 0.73 | 1.09 | 0.73 | 0.73 | 0.73 | 0.73 | 0.73 | 0.73 |
| 8. S16S08 | 1 | 2 | 2 | 2 | 2 | 2 | 2 | - | 0.73 | 1.09 | 0.73 | 0.73 | 1.09 | 0.73 | 0.73 | 0.73 | 0.73 | 0.73 | 0.73 |
| 9. S16S09 | 2 | 3 | 3 | 3 | 3 | 3 | 3 | 2 | - | 1.45 | 1.09 | 1.09 | 1.45 | 0.36 | 1.09 | 1.09 | 1.09 | 1.09 | 1.09 |
| 10. S16S10 | 2 | 3 | 3 | 3 | 3 | 3 | 3 | 3 | 4 | - | 1.09 | 1.09 | 1.45 | 1.09 | 1.09 | 1.09 | 1.09 | 1.09 | 1.09 |
| 11. S16S11 | 1 | 2 | 2 | 2 | 2 | 2 | 2 | 2 | 3 | 3 | - | 0.73 | 1.09 | 0.73 | 0.73 | 0.73 | 0.73 | 0.73 | 0.73 |
| 12. S16S12 | 1 | 2 | 2 | 2 | 2 | 2 | 2 | 2 | 3 | 3 | 2 | - | 1.09 | 0.73 | 0.73 | 0.73 | 0.73 | 0.73 | 0.73 |
| 13. S16S13 | 2 | 3 | 3 | 1 | 3 | 3 | 3 | 3 | 4 | 4 | 3 | 3 | - | 1.09 | 1.09 | 1.09 | 1.09 | 1.09 | 1.09 |
| 14. S16S14 | 1 | 2 | 2 | 2 | 2 | 2 | 2 | 2 | 1 | 3 | 2 | 2 | 3 | - | 0.73 | 0.73 | 0.73 | 0.73 | 0.73 |
| 15. S16S15 | 1 | 2 | 2 | 2 | 2 | 2 | 2 | 2 | 3 | 3 | 2 | 2 | 3 | 2 | - | 0.73 | 0.73 | 0.73 | 0.73 |
| 16. S16S16 | 1 | 2 | 2 | 2 | 2 | 2 | 2 | 2 | 3 | 3 | 2 | 2 | 3 | 2 | 2 | - | 0.73 | 0.73 | 0.73 |
| 17. S16S17 | 1 | 2 | 2 | 2 | 2 | 2 | 2 | 2 | 3 | 3 | 2 | 2 | 3 | 2 | 2 | 2 | - | 0.73 | 0.73 |
| 18. S16S18 | 1 | 2 | 2 | 2 | 2 | 2 | 2 | 2 | 3 | 3 | 2 | 2 | 3 | 2 | 2 | 2 | 2 | - | 0.73 |
| 19. S16S20 | 1 | 2 | 2 | 2 | 2 | 2 | 2 | 2 | 3 | 3 | 2 | 2 | 3 | 2 | 2 | 2 | 2 | 2 | - |

Numbers above the diagonal are percent distance values; numbers below the diagonal are absolute distance values.
